# Genomics-assisted prediction of salt and alkali tolerances and functional marker development in apple rootstocks

**DOI:** 10.1186/s12864-020-06961-9

**Published:** 2020-08-10

**Authors:** Jing Liu, Fei Shen, Yao Xiao, Hongcheng Fang, Changpeng Qiu, Wei Li, Ting Wu, Xuefeng Xu, Yi Wang, Xinzhong Zhang, Zhenhai Han

**Affiliations:** College of Horticulture, China Agricultural University, Beijing, China

**Keywords:** Saline tolerance; alkaline tolerance, QTL mapping, *Malus* ssp., Genetic variation

## Abstract

**Background:**

Saline, alkaline, and saline-alkaline stress severely affect plant growth and development. The tolerance of plants to these stressors has long been important breeding objectives, especially for woody perennials like apple. The aims of this study were to identify quantitative trait loci (QTLs) and to develop genomics-assisted prediction models for salt, alkali, and salt-alkali tolerance in apple rootstock.

**Results:**

A total of 3258 hybrids derived from the apple rootstock cultivars ‘Baleng Crab’ (*Malus robusta* Rehd., tolerant) × ‘M9’ (*M. pumila* Mill., sensitive) were used to identify 17, 13, and two QTLs for injury indices of salt, alkali, and salt–alkali stress via bulked segregant analysis. The genotype effects of single nucleotide polymorphism (SNP) markers designed on candidate genes in each QTL interval were estimated. The genomic predicted value of an individual hybrid was calculated by adding the sum of all marker genotype effects to the mean phenotype value of the population. The prediction accuracy was 0.6569, 0.6695, and 0.5834 for injury indices of salt, alkali, and salt–alkali stress, respectively. SNP182G on *MdRGLG3*, which changes a leucine to an arginine at the vWFA-domain, conferred tolerance to salt, alkali, and salt-alkali stress. SNP761A on *MdKCAB*, affecting the Kv_beta domain that cooperated with the linked allelic variation SNP11, contributed to salt, alkali, and salt–alkali tolerance in apple rootstock.

**Conclusions:**

The genomics-assisted prediction models can potentially be used in breeding saline, alkaline, and saline-alkaline tolerant apple rootstocks. The QTLs and the functional markers may provide insight for future studies into the genetic variation of plant abiotic stress tolerance.

## Background

Saline and alkali stress seriously affect growth, development, and plant survival. Excessive salt disturbs the ion balance in soil, causing ion toxicity and osmotic stress, thus affecting plant growth [1–3]. Alkaline stress, such as Na_2_CO_3_, can cause more damage to plants than neutral salts [4–6] because under alkaline stress, plants have to withstand the same osmotic stress and ion toxicity as salt stress, as well as high pH stress [7–10]. Natural salt stress often occurs as mixed salt stress with both neutral and alkaline salts. Soil salinization and alkalization frequently co-occur, causing much severe problems [11]. The effects of salt–alkali stress may be more severe than any other abiotic stressor [7, 12–14].

Compared to the comprehensive soil control and optimized regionalization of farming, the most cost-efficient strategy for coping with the stress is breeding and utilizing high saline and alkali tolerant cultivars [15, 16]. Hence understanding the inheritance and genetics of the saline and alkali tolerances is a prerequisite of modern breeding practices [17].

Salt and alkali tolerance are partially independent traits, salt-tolerant varieties are not necessarily highly alkali-tolerant. Tolerances to salt and alkali stress are quantitative traits controlled by a few major genes and several minor genes [18–20]. The heritability of salt stress tolerance depends on the species and cultivar, ranging from 38.18% ~ 66.64% in lentil (*Lens culinaris* M.) to 75% ~ 83.1% in tomato (*Solanum lycopersicum* L.) and upland cotton (*Gossypium hirsutum* L.) [21–23]. Numerous quantitative trait loci (QTLs), including some major QTLs, associated with salinity tolerance trait have been identified in rice (*Oryza sativa* L.), tomato, *Arabidopsis thaliana* L., and cotton [24–27]. Similarly, numerous minor and a few major QTLs for alkali tolerance were mapped in crops [28, 29]. However, there are only a few QTLs overlapping between salt and alkali stress tolerance, 83.3% of which were independent of each other. This indicates that the genetic control of the tolerance to these two stressors might be partially independent.

Diagnostic or functional markers, designed on the functional gene body or the promoter region, can be accurately used in marker assisted selection (MAS). Therefore, many studies have been conducted to predict candidate genes from QTL confident intervals and to test the molecular functions of the genetic variations [30–34]. For example, nonsynonymous single nucleotide polymorphisms (SNPs) of *GhGPAT16* and *GhGPAT26* in cotton were significantly correlated with cotton oil content under salt stress [35]. A T/C SNP at TaCRT-D plays an important role in the salt tolerance of wheat (*Triticum aestivum* L.) [36].

When a trait is controlled by a few loci with major effects and is less affected by environmental factors, MAS is a cost-efficient strategy for selecting appropriate genotype combinations [37, 38]. In contrast, many agronomic traits, such as salt and alkali tolerance, are often affected by polygenes with minor effects. In this scenario, genomic selection (GS) should be an effective alternative [39–41]. GS explores the contribution of whole genome markers for a certain trait, but the cost of GS is high because it requires a larger population size and higher marker density [42]. Fully utilizing QTL-based markers in GS modeling might be a solution for addressing the cost-efficiency of GS [38].

The aims of this study were to identify genome-wide QTLs associated with tolerance to salt, alkali, and combined salt–alkali stress, and to develop QTL-based genomics-assisted prediction (GAP) models in apple rootstocks. A bulked segregant analysis via next generation resequencing (BSA-seq) was used for QTL detection. Then, the effects of each QTL-based marker were estimated and GAP models were developed and validated. The GAP models obtained in this study can assist breeding for salt and alkali stress tolerance in asexually propagated perennial crops such as apple.

## Results

### Phenotype segregation and analysis of inheritance

**Fig. 1:**
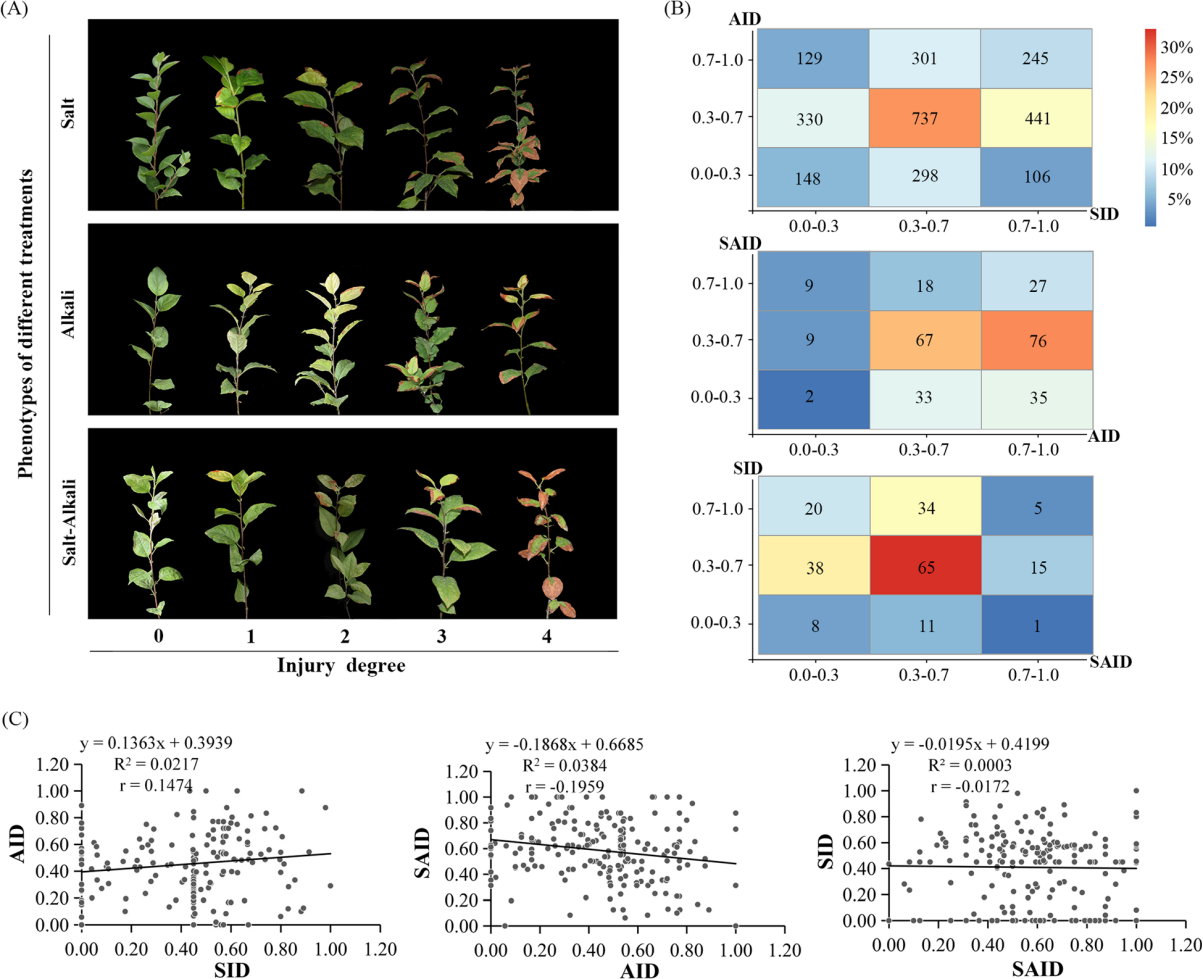
Categorical criteria of injury indices and pairwise relationship between injury indices of salt (SID), alkali (AID), and salt-alkali (SAID) stress in apple rootstock hybrids of *Malus robusta* Rehd. ‘Baleng Crab’ × *M. pumila* Mill. ‘M9’. **a** Photograph showing scoring criteria of injury degrees of salt, alkali, and salt–alkali stress. **b** Heatmap showing the pairwise relationship between numbers of hybrids tolerant or sensitive to salt, alkali, and salt–alkali stress. The number of hybrids are presented in the blocks and the percentage of hybrids by column is illustrated by color gradients. **c** Linear regressions between SID, AID, and SAID

By using leafy cutting propagated plants of F1 hybrids from *Malus robusta* Rehd. ‘Baleng Crab (BC)’ × *M. pumila* Mill. ‘M9’, salt injury index (SID), alkali injury index (AID), and salt-alkali injury index (SAID) were phenotyped in 2015–2017. The phenotype exhibited broad segregation ranging with averages of 0.46, 0.48, and 0.57 for SID, AID, and SAID, respectively (Fig. 1a; Fig. S1; Table 1; Table S1, S2, S3). The overall mean phenotype value of the population for SID, AID, and SAID was approximately equal to that of to their mid-parent value, respectively (Table 1). The segregation spectrums were nearly Gaussian distributed (Fig. S1). The broad sense heritability of SID, AID, and SAID was 63.28, 62.26 and 60.69%, respectively (Table 1).

**Table 1:** Phenotype segregation and inheritance of salt, alkali, salt–alkali injury indices in apple rootstock hybrids of *Malus robusta* Rehd. ‘Baleng Crab’ × *M. pumila* Mill. ‘M9’

Trait	Year	Phenotype mean	Standard deviation	Coefficient of variance	Broad sense heritability
Salt injury index	2015	0.53	0.28	52.08%	63.28%
	2016	0.40	0.28	69.05%	
	2017	0.43	0.28	63.79%	
Alkali injury index	2015	0.51	0.25	49.17%	62.26%
	2016	0.46	0.26	56.41%	
	2017	0.46	0.24	52.98%	
Salt-alkali injury index	2016	0.60	0.26	42.67%	60.69%
	2017	0.55	0.26	46.91%	

The Pearson’s correlation coefficient was 0.1474 (*p* < 0.05) between SID and AID, indicating a weak positive correlation. The Pearson’s correlation coefficient between SID and SAID was − 0.0172 (*p* > 0.1). For AID and SAID, the Pearson’s correlation coefficient was − 0.1959 (*p* < 0.01) (Fig. 1c), indicating a negative correlation between AID and SAID. We found that not all of the salt-tolerant plants were alkali-tolerant, while salt-sensitive plants were not necessarily all alkali-sensitive (Fig. 1b).

### Identification of QTLs via BSA-seq

**Fig. 2:**
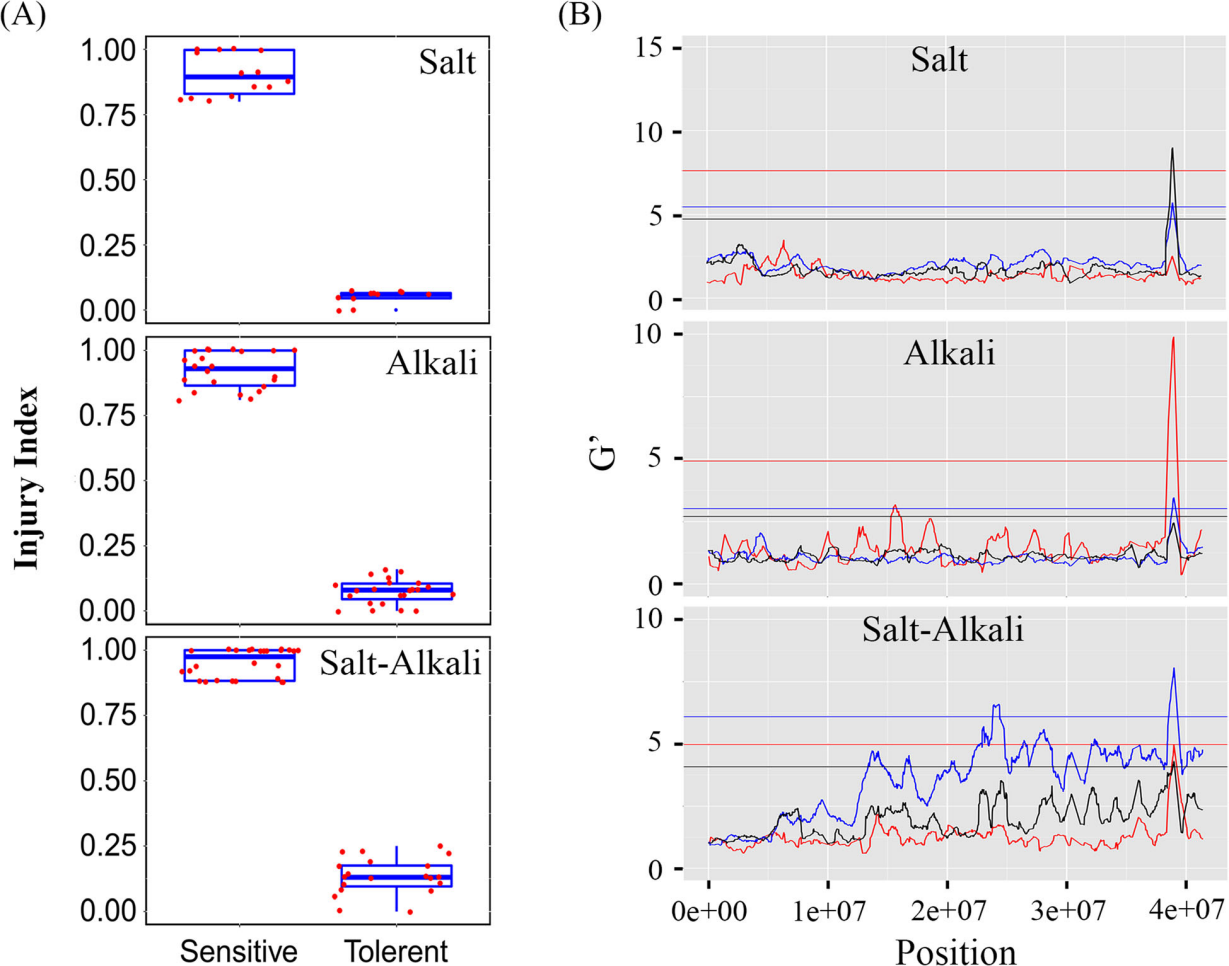
Diagrams showing phenotypes of the six segregant bulks with phenotype extremes for tolerant or sensitive hybrids (**a**) and common QTLs on chromosome 16 (**b**) for salt, alkali, and salt–alkali injury indices identified using bulked segregant analysis by sequencing F1 hybrids of *Malus robusta* Rehd. ‘Baleng Crab (BC)’ × *M. pumila* Mill. ‘M9’ rootstocks. In panel B, the Y-axis represents the G’ value and the X-axis represents the physical position on the chromosome. The red curved lines: ‘M9’; blue curved lines: ‘BC’; black curved lines: ‘BC’ & ‘M9’. The colored horizontal lines indicate the corresponding statistically significant threshold of the G’ value

Based on the phenotyping data from 2015 to 2017, six segregant bulks were developed, each including 11–26 hybrids with extreme phenotypes for tolerance or sensitivity to salt, alkali, or salt–alkali stress (Fig. 2a; Table S4). Sequencing of the six bulk DNA samples generated a total of 611,037,786 clean reads, of these reads, 97.77% were mapped to the apple ‘Golden Delicious’ dihaploid GDDH genome and 74.54% were uniquely mapped (Table S5). Seventeen QTLs for SID were mapped on chromosomes 2, 7, 11, 14, 15, and 16 using BSATOS software [43]. This included three QTLs with large G’ values, S-M02.1, S-BC15.1, and S-H16.1 (Fig. S2). For AID, 13 QTLs were identified, and the G’ value of A-BC09.1, A-M06.1, and A-M16.1 were greater than the other 10 QTLs (Fig. S3). Only two QTLs were detected to be associated with SAID, and the G’ value of SA-BC16.2 was relatively large (Fig. S4). Among these QTLs, S-H16.1, A-M16.1, and SA-BC16.2 on chromosome 16 overlapped each other (Fig. 2b); QTLs A-M02.1, S-M02.3, and S-H02.1 on chromosome 2 were partially overlapping (Table S6).

### Candidate gene prediction via parental resequencing data

DNA sequences of 2657 genes within the 32 QTL intervals were downloaded from the apple ‘Golden Delicious’ dihaploid GDDH genome database, and then the gene expression was analyzed using RNA-seq data. Of the total number of genes, 199 were excluded because they did not show expression. Another 410 genes were culled because the genetic variations were inconsistent with the parent from which the QTL was mapped. We also excluded 883 genes for which functional annotation was not related to the targeted trait. In addition, 475 genes with variations on the promoter region but absent from the list of differentially expressed genes (DEGs) were excluded. Finally a total of 425, 248, and 17 candidate genes were predicted for SID, AID, and SAID, respectively (Table S7, S8).

### RNA-seq analysis for salt, alkali, and salt–alkali tolerances

To show the differences in gene expression between hybrids that were tolerant or sensitive to salt, alkali, and salt-alkali stress, RNA-seq was performed. RNA-seq generated 289.94 Gb clean data with a Q30 value of 92.85. For each sample, over 3.04 Gb clean reads were generated, with an average GC content of 46.93%. The average proportion of total reads mapped to the reference genome was 86.05% (Table S9, S10, S11). A total of 875, 658, and 5359 DEGs were identified between hybrids which were tolerant/sensitive to salt, alkali, and salt-alkali, respectively (Table S12, S13, S14).

**Fig. 3:**
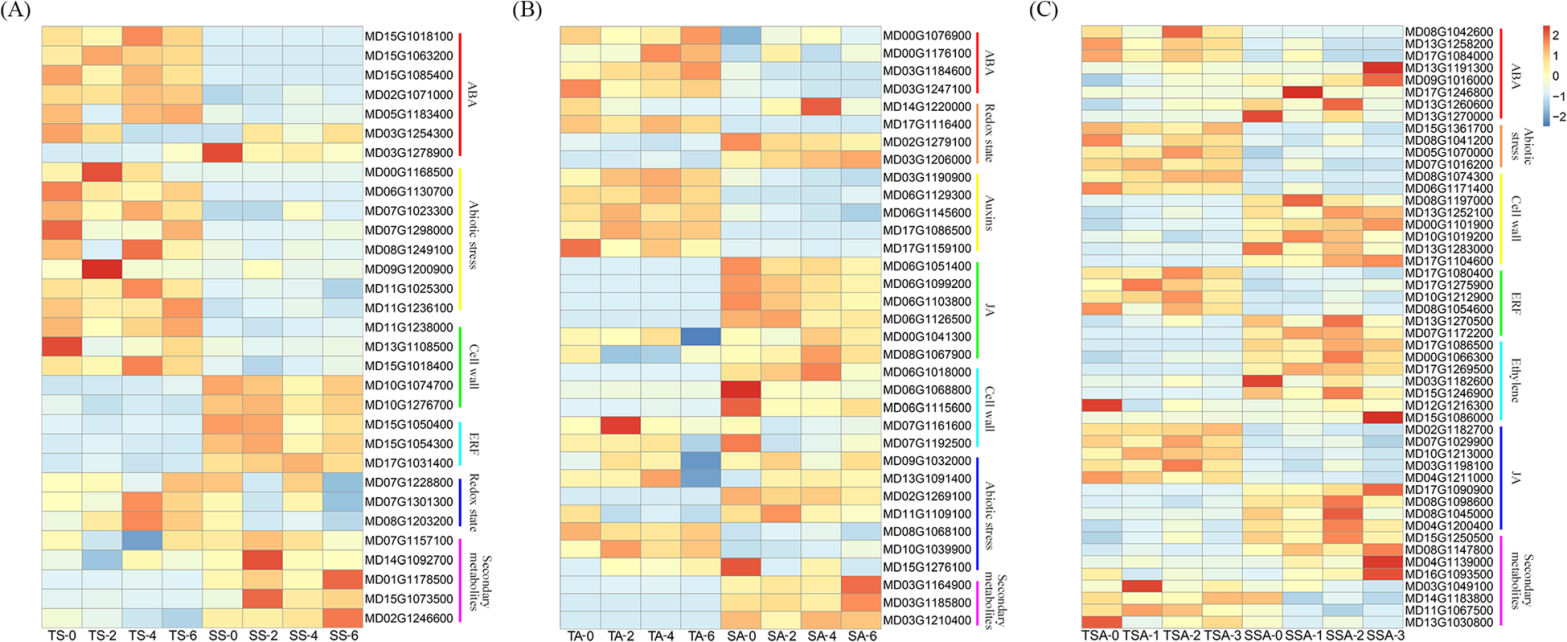
Heatmaps showing differentially expressed genes between individuals that were tolerant or sensitive to salt (**a**), alkali (**b**), and salt–alkali stress (**c**) in F1 hybrids of *Malus robusta* ‘Baleng Crab’ × *M. pumila* ‘M9’ rootstocks

The expression profiles differed among salt, alkali, and salt–alkali stress tolerant hybrids compared to sensitive hybrids. The expression of most abscisic acid (ABA) signaling related genes were lower throughout the experiment in saline or alkaline sensitive hybrids; some ABA related genes were considerably higher in salt–alkali sensitive hybrids compared to the other hybrids (Fig. 3a-c). Conversely, the transcription of genes related to secondary metabolites were upregulated in saline and alkaline sensitive hybrids (Fig. 3a and c). In salt or salt–alkali tolerant hybrids, the expression of nearly all the abiotic stress response genes were higher, but most ethylene biosynthesis or signaling genes were expressed at lower levels (Fig. 3a and c). A large proportion of cell wall associated genes had a greater level of expression in alkali and salt–alkali sensitive hybrids (Fig. 3b and c). Most jasmonate (JA) related genes were highly expressed in alkali sensitive hybrids, while exhibited upregulation in salt–alkali sensitive hybrids (Fig. 3b and c).

**Fig. 4:**
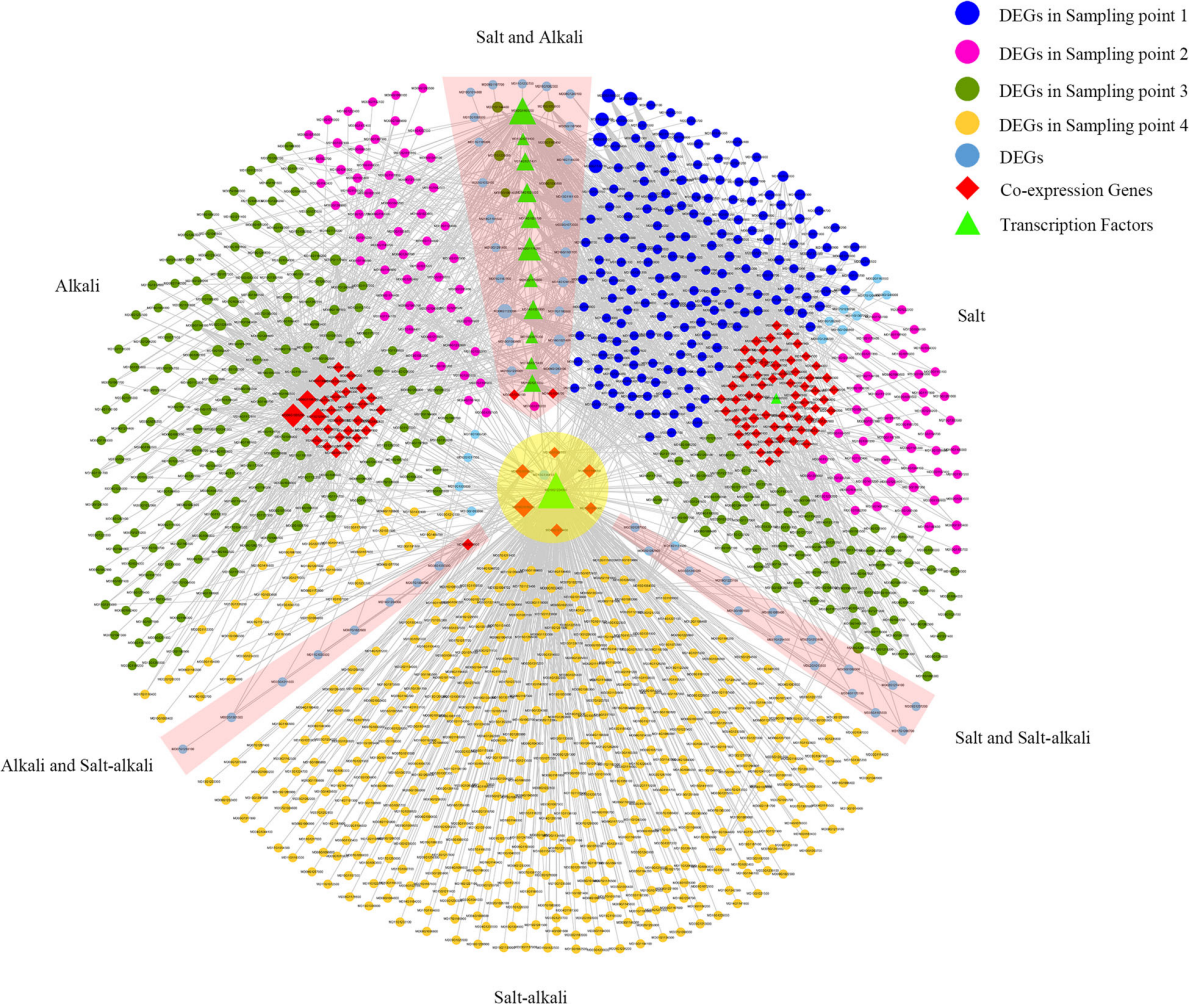
Co-expressions network of differentially expressed genes (DEGs) between individuals that were tolerant or sensitive to of salt, alkali, and salt–alkali stress conditions in F1 hybrids of *Malus robusta* ‘Baleng Crab’ × *M. pumila* ‘M9’ rootstocks

To test the interaction among DEGs, the co-expression network was analyzed. The majority of DEGs responded at early stage (day 0–4) to salt stress than to alkali (at day 4) or salt–alkali stress (at day 3) (Fig. 4). A GATA transcription factor (TF) gene, MD16G1234900, and several ubiquitin related genes co-expressed with DEGs in tolerant to salt, alkali, and salt–alkali hybrids compared to sensitive hybrids (Fig. 4; Table S15). MD16G1234900 was located in the confident interval of QTL SA-BC16.1 (Table S8). Similarly, 13 transcription factors co-expressed with a group of DEGs between hybrids that were tolerant to saline or alkaline stress (Fig. 4; Table S15). In response to salt stress, a specific co-expression network was found to include a GATA12 gene (MD15G1092500) and several heat shock protein genes. Another co-expression network of 29 DEGs responded to alkaline stress (Fig. 4; Table S15). MD16G1235900, a voltage-gated potassium channel subunit beta gene *MdKCAB,* also located within QTL SA-BC16.1 interval, co-expressed with DEGs of saline–alkaline tolerant hybrids (Table S8).

### Development of GAP models

To develop the GAP models, a SNP was chosen from every candidate gene to design markers for kompetitive allele-specific PCR (KASP). A total of 17, 13, and two KASP markers were designed for the traits SID, AID, and SAID, respectively (Table S16).

The marker SH14275 from QTL S-H14.1 exhibited the largest marker effect (0.4235); the marker effect (0.0354) of SM1111 from QTL S-M11.1 had the least effect on SID (Table S17). For alkali tolerance, the largest marker effect (0.2134) on AID was estimated in AB1614 derived from QTL A-BC16.1, whereas marker nn174 from QTL A-M06.1 (0.0119) had the lowest marker effect (Table S17). For SAID, the effects of both the markers SAB161 and SAB162 designed on QTLs SA-BC16.1 and SA-BC16.2, respectively, were as large as 0.2391 and 0.2947 (Table S17).

**Fig. 5:**
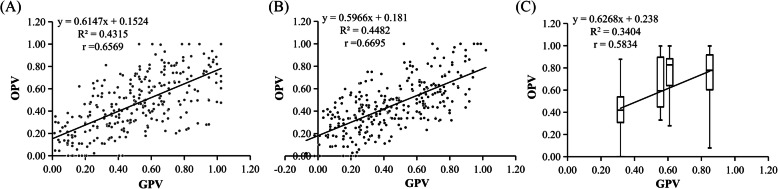
Linear regression between genomics predicted value (GPV) and observe phenotype value (OPV) of injury indices of salt (**a**), alkali (**b**), and salt–alkali stress (**c**) in F1 hybrids of *Malus robusta* ‘Baleng Crab’ × *M. pumila* ‘M9’ rootstocks. Because there were only four genotype combinations in (**c**), the data dots should appeared to form four vertical lines, by which it is difficult to see how much the data are segregating, so, box plot was used to show the deviation, the mean value, and the standard variance

To calculate genomics predicted value (GPV), the genotype effects of all markers for SID, AID, and SAID were added to the overall value of the mean phenotype for an individual hybrid (Table S18, S19, S20). The prediction accuracy, represented by Pearson’s correlation coefficients between GPV and the observed phenotype value (OPV) of individual hybrids, was 0.6569, 0.6695, and 0.5834 for SID, AID, and SAID, respectively (Fig. 5). Five-fold cross validation confirmed the accuracy of the predictability of GPV by 0.6587, 0.6573, and 0.6534 for SID, AID, and SAID, respectively (Table S21).

In the simulative selection, when GPV criteria were set as 0.2, the selection rates were 16.84 and 18.06% for SID and AID, respectively (Table 2). The selection efficiencies, represented by the percentage of individuals for which OPV is consistent with the corresponding GPV, were 58.33 and 40.74% for SID and AID, respectively (Table 2). For SAID, if the GPV criterion was 0.32, the selection rate was as high as 42.78%, and the selection efficiency was as relatively low as 31.33% (Table 2).

**Table 2:** Simulative selection of salt, alkali, and salt–alkali stress tolerant individuals of apple rootstocks by genomics-assisted prediction in an F1 population derived from *Malus robusta* Rehd. ‘Baleng Crab’ × *M. pumila* Mill. ‘M9’

Trait	GPV criterion	Number of selections	Selection rate	GPV	Number of selections (OPV ≤ GPV)	Efficiency
Salt injury index	≤0.10	24	8.42%	0.06	13	54.17%
	≤0.20	48	16.84%	0.11	28	58.33%
	≤0.30	74	25.96%	0.16	35	47.30%
	≤0.40	106	37.19%	0.21	41	38.68%
	≤0.50	139	48.77%	0.27	45	32.37%
Alkali injury index	≤0.10	23	7.69%	0.05	13	56.52%
	≤0.20	54	18.06%	0.1	22	40.74%
	≤0.30	102	34.11%	0.17	33	32.35%
	≤0.40	138	46.15%	0.22	39	28.26%
	≤0.50	174	58.19%	0.27	42	24.14%
Salt-alkali injury index	≤0.12	0	0.00%	0	0	0.00%
	≤0.22	0	0.00%	0	0	0.00%
	≤0.32	83	42.78%	0.4142	26	31.33%
	≤0.42	83	42.78%	0.4142	26	31.33%
	≤0.52	83	42.78%	0.4142	26	31.33%

### Validation of functional variations in the candidate gene *MdRGLG3*

**Fig. 6:**
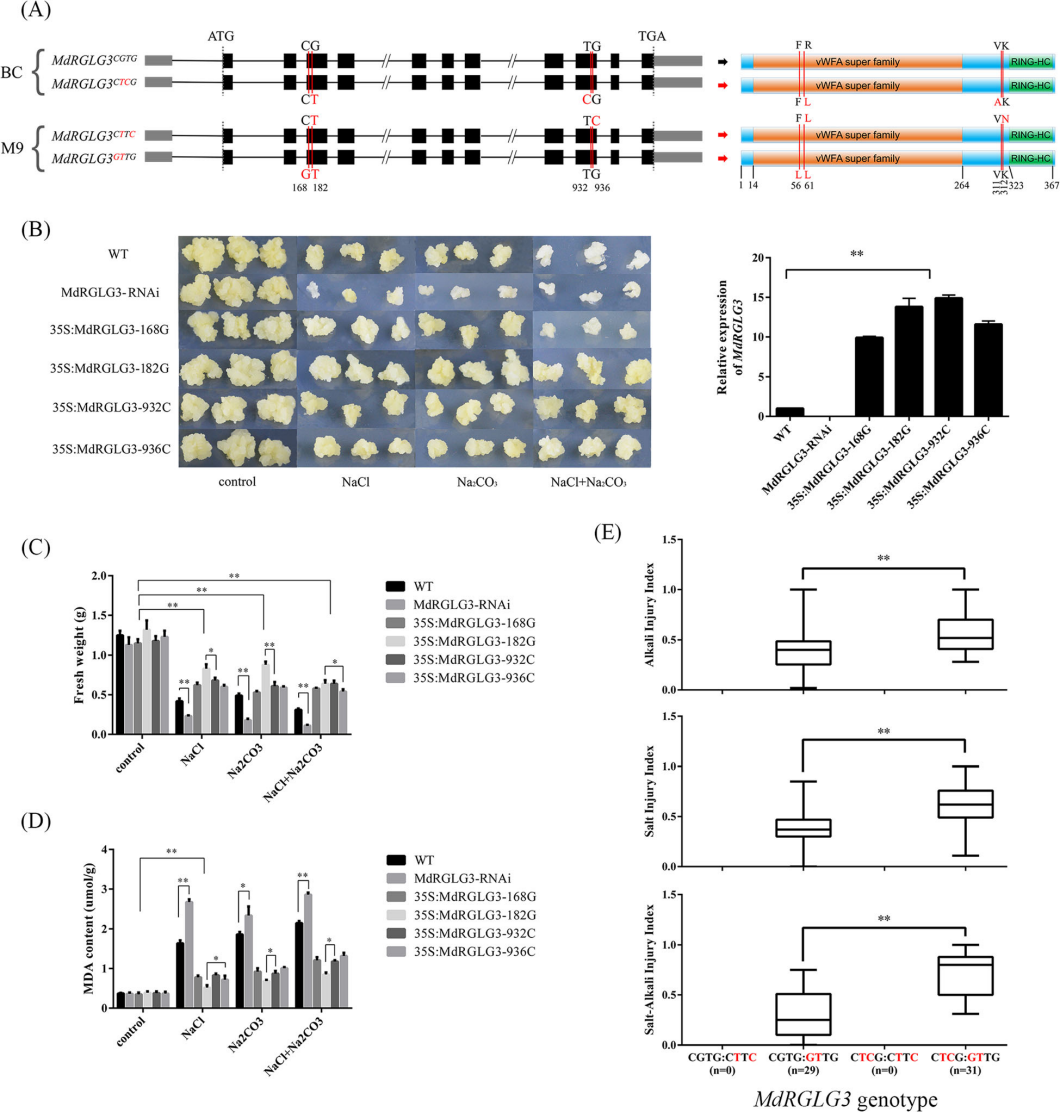
Schematic showing genetic variations on the coding sequence of *MdRGLG3* and functional validation of these variations via callus transgenesis and genotype–phenotype association. **a**: Schematic diagram showing single nucleotide polymorphisms on the coding region and variations in the amino acid sequences of *MdRGLG3*. **b**: Images showing in vitro growth of transformed apple callus overexpressing variants of *MdRGLG3* and the MdRGLG3-RNAi line on media containing high levels of NaCl, Na_2_CO_3_, or NaCl + Na_2_CO_3_. C and D: Column blots showing differences in apple callus fresh weight (**c**) and malondialdehyde (MDA) content (**d**) 14 days after treatment with NaCl, Na_2_CO_3_, or NaCl + Na_2_CO_3_, among transformants overexpressing variants of *MdRGLG3* or the MdRGLG3-RNAi line. E: Box-plot showing injury indices of salt, alkali, and salt–alkali stress in F1 hybrids of ‘Baleng Crab’ × ‘M9’ with different genotype combinations of *MdRGLG3* SNP168, SNP182, SNP932, and SNP936. Data are means ± standard deviation of three biological replicates. *, **, and *** indicate statistically significant differences at *P* < 0.05, *P* < 0.01, and *P* < 0.001, respectively

From the overlapping QTLs, S-H16.1, A-M16.1, and SA-B16.2 on chromosome 16, *MdRGLG3* (MD16G1282700) was predicted as a candidate gene for SAID. The full length coding sequence (CDS) of *MdRG*LG3 was 1107 bp, encoding an E3 ubiquitin-protein ligase with 369 amino acid residues. In the CDS of *MdRGLG3*, two nonsynonymous heterozygous SNPs were found at + 182 bp (SNP182) and + 932 bp (SNP932) from the ATG codon in the maternal parent ‘BC’. SNP182 G/T may change a leucine to an arginine at the vWFA-domain; SNP932 T/C leads to an amino acid substitution of valine with alanine (Fig. 6a). Two nonsynonymous SNPs, SNP168 C/G at + 168 bp and SNP936 G/C at + 936 bp from the ATG codon, were detected in the pollen parent ‘M9’, causing a change from phenylalanine to leucine and from lysine to asparagine, respectively (Fig. 6a). These SNPs were then confirmed by Sanger sequencing (Fig. 6a; Fig. S5). There were 16 SNPs and insertion/deletions (InDels) located in the 1600 bp DNA fragment upstream of the ATG codon of *MdRGLG3* in ‘M9’ but not ‘BC’ (Supplementary File 1). In the root tissue of ‘BC’ and ‘M9,’ no differences in *MdRGLG3* expression were detected in fragments per kilobase per million (FPKM) by RNA-seq or in the relative expression determined by qPCR, indicating that the promoter variations did not alter the gene expression (Supplementary File 1; Fig. S6A).

To further confirm the function of the variations in *MdRGLG3* CDS, the variants were transformed into apple callus. The in vitro growth of both *MdRGLG3* over-expression and MdRGLG3-RNAi apple calli was similar with the untransformed wild type (WT) under normal conditions. However, the MdRGLG3-RNAi line exhibited severely less fresh weight and higher malondialdehyde (MDA) content 14 days after treatments with either salt, alkali or salt–alkali (Fig. 6b and d). After 14 days under salt, alkali, or salt–alkali stress conditions, the fresh weight of 35S:MdRGLG3-182G apple calli was significantly higher, whereas the MDA content was significantly lower, than the other transformants and the WT (Fig. 6c and d). The phenotype values of SID, AID, and SAID in F1 hybrids with the *MdRGLG3* SNP182G allele were significantly lower than that in hybrids without the SNP182G allele (Fig. 6e). SNP182G was closely linked to the tolerance genotype of the KASP marker A-BC16.1 (Table S22). These data indicated that *MdRGLG3* SNP182G allele was a functional variation leading to increased tolerance to salt, alkali, and salt–alkali conditions. One hundred *Malus* accessions were then genotyped for the *MdRGLG3* SNP182 allele; the genotype frequency of the SNP182G:G homozygote was as low as 1%, compared with 27 and 72% for SNP182G:T and SNP182T:T, respectively, indicating a possibly lethal effect of the SNP182G:G genotype (Table S24).

### Validation of functional variations in the candidate gene *MdKCAB*

**Fig. 7:**
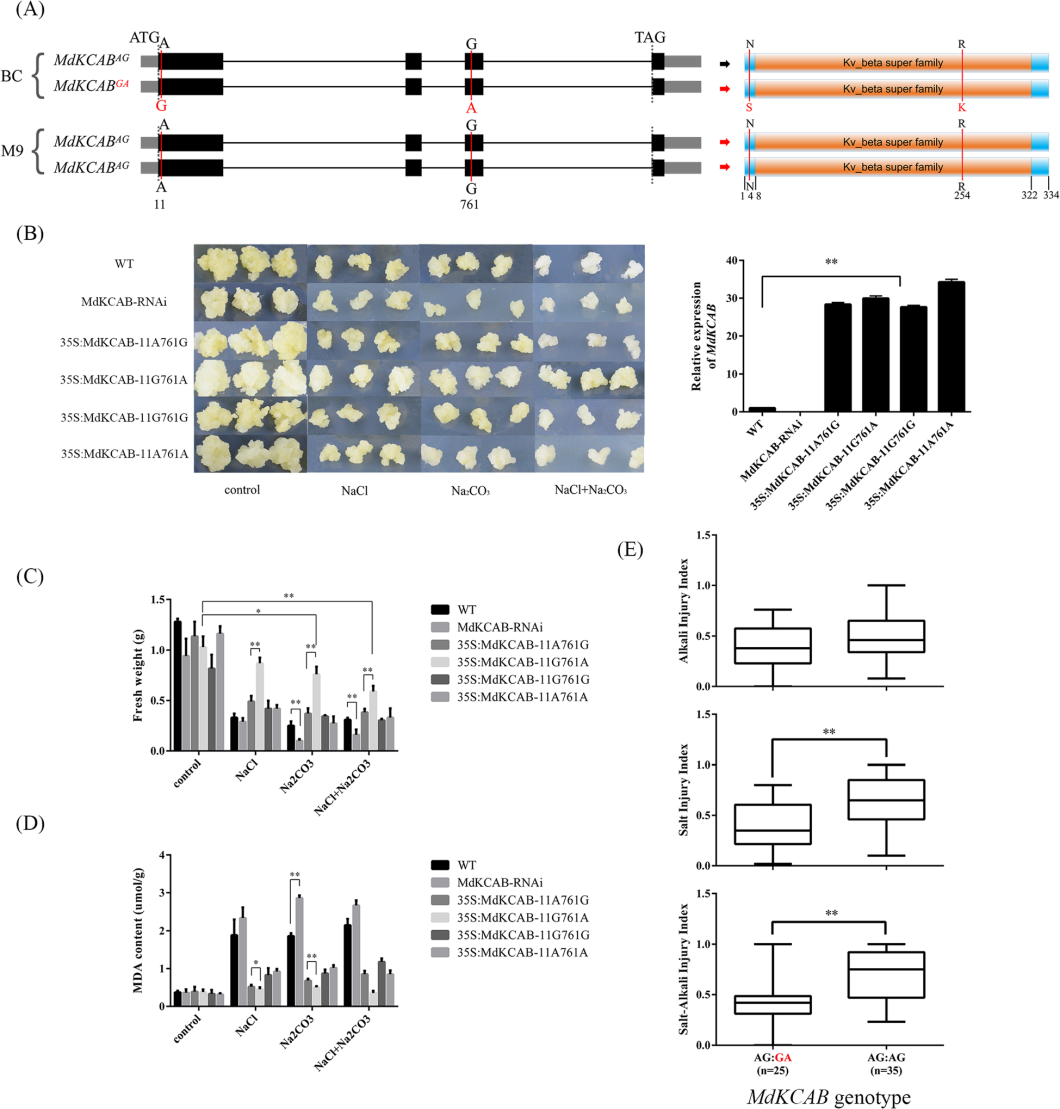
Schematic diagram showing genetic variations of the coding sequence of *MdKCAB* and the functional validation of these variations via callus transgenesis and genotype–phenotype association. **a**: Schematic diagram showing single nucleotide polymorphisms on the coding region and the changes in the encoded amino acids of *MdKCAB*. **b**: Images showing in vitro growth of transformed apple callus overexpressing variants of *MdKCAB* and the MdKCAB-RNAi line 14 days after treatment with NaCl, Na_2_CO_3_, or NaCl + Na_2_CO_3_. **c** and **d**: Column blots showing differences in the fresh weight of the apple callus (**c**) and malondialdehyde (MDA) content (**d**) 14 days after treatment with NaCl, Na_2_CO_3_ or NaCl + Na_2_CO_3_ among transformants overexpressing variants of *MdKCAB* or the MdKCAB-RNAi line. E: Box-plot showing injury indices of salt, alkali, and salt–alkali stress in F1 hybrids of ‘Baleng Crab’ × ‘M9’ with different genotype combinations of *MdKCAB* SNP11 and SNP761. Data are means ± standard deviation of three biological replicates. *, **, and *** indicate statistically significant differences at *P* < 0.05, *P* < 0.01, and *P* < 0.001, respectively

From QTL SA-B16.1, *MdKCAB* (MD16G1235900) on chromosome16 was predicted as a candidate gene for salt–alkali tolerance. The full length CDS of *MdKCAB* was 1002 bp, encoding 334 amino acid peptide. The expression of *MdKCAB* did not differ between the parents, ‘BC’ and ‘M9’ (Fig. S6B). No variations altering the cis-element in the 1.5 kb upstream region were detected in either ‘BC’ or ‘M9’ (Supplementary File 2). However, two nonsynonymous SNPs were detected at + 11 bp and + 761 bp from the ATG codon in ‘BC’ but not in ‘M9’ (Fig. 7a). These SNPs were confirmed by Sanger sequencing (Fig. S7). SNP11 A/G and SNP761 G/A led to a substitution from aspartic acid to serine and from arginine to lysine, respectively (Fig. 7a; Fig. S7).

Under normal conditions, transformed apple calli overexpressing *MdKCAB* or MdKCAB-RNAi showed no difference in in vitro growth compared to the WT, irrespective of genotypes. Under salt, alkali, or salt–alkali treatments, the growth of MdKCAB-RNAi calli was significantly reduced after 14 days of culture (Fig. 7b and c). When grown under salt, alkali, or salt–alkali stress conditions, *MdKCAB* overexpressed calli showed better proliferation and lower levels of MDA than the untransformed WT and MdKCAB-RNAi line (Fig. 7c and d). After 14 days of salt, alkali, or salt–alkali stress conditions, the callus transformed with 35S:MdKCAB-11G761A had a significantly higher fresh weight and lower MDA content, while other transformants had no significant difference (Fig. 7c and d). The phenotype values of SID, AID, and SAID in F1 hybrids with SNP11G/SNP761A alleles of *MdKCAB* were significantly lower than that of hybrids without these alleles. SNP11G761A was closely linked to the tolerance genotype of the SA-BC16.1 KASP marker (Fig. 7e, Table S23). These data indicated that SNP11G and SNP761A of *MdKCAB* both contributed to the improved tolerance of apple rootstock to salt, alkali, and salt–alkali stress conditions.

### Changes in osmolality of Hoagland’s solution caused by the addition of NaCl and/or Na_2_CO_3_

The addition of NaCl and/or Na_2_CO_3_ in the nutrient solutions in alkaline, saline, and alkaline-saline stress treatments led to significant increases in osmolality of the solutions (Fig. S8). The osmolality values in the solutions for alkali (1.75 MPa), salt (2.73 MPa), and alkali-salt (5.12 MPa) treatments were 2, 3, and 5 times that of non-stress control (0.82 MPa) (Fig. S8).

## Discussion

### Tolerance to salt, alkali, and salt–alkali stress overlapped genetically

Salt stress and alkali stress are usually accompanied by or closely associated with each other in environments, and plant tolerances to salt and alkali are often genetically and physiologically linked but distinct [7–10]. We found that the correlation between SID and AID was relatively weak. In addition, the salt-tolerant hybrids that had a low SID (0.0 ~ 0.3) segregated for AID and vice versa. Of the 17, 13, and two QTLs linked to SID, AID, and SAID, S-H16.1/A-M16.1/SA-BC16.2 on chromosome 16 and A-M02.1/S-M02.3/S-H02.1 on chromosome 2 overlapped, explaining the genetic linkage of these traits. The other QTLs that did not overlap highlight the independent segregation of salt, alkali, and salt–alkali tolerances. These results were consistent with previous reports in cereal crops [20, 44, 45]. The allelic variation of *MdRGLG3* due to SNP182G conferred tolerance to either salt, alkali, or salt–alkali stress. RNA-seq data showed that the regulatory pathways and related DEGs under salt or alkali stress were different from those under salt–alkali stress, indicating that the mechanism of genetic diversity of these stress tolerances were not the same.

Recently, genetic network and molecular mechanism of plant stress tolerances have been increasingly explored via multi-omics strategies, such as RNA-seq, QTL mapping, and co-expression tools [43, 46]. In this study, a GATA TF gene, MD16G1234900, was identified as important candidate gene associated with salt, alkali, and salt–alkali tolerance by using co-expression analysis. Consistently, the GATA TF gene was located in the confident interval of QTL SA-BC16.1. GATA TF regulates many ubiquitin related genes that are responsive to saline or alkaline stress [47, 48]. Similarly, 13 TF genes were co-expressed with a group of DEGs related to salt or alkali tolerance (Fig. 4; Table S15). Of these TFs, ABI5 responds to stress-induced ABA and co-expresses with ERF10 and NHL network to regulate salt or alkali tolerance [49–51].

We detected a salt stress specific co-expression network including a series of heat shock protein genes. Plant heat stress TFs regulate responses not only to high temperatures but also to diverse environmental stresses [52–54]. HSFA4A of *Arabidopsis* promotes the expression of a wide range of defense genes and regulates tolerance to salinity [55].

In this study, a unique co-expression network in response to alkaline stress was detected including 29 DEGs involved in ubiquitination signaling, oxidative stress and plant cell wall. Of these genes, RD23D is a shuttle factor for the transport of ubiquitinated substrates to the proteasome in response to salinity and abscisic acid [56]. IQM1 plays a role in the regulation of ubiquitination and ROS signaling [57]. We also found several oxidative stress related genes, like PLDG, ASO, and MJ1384, and cell wall associated genes, such as ABCG29, WAK3 and MCD1, co-expressed in response only to alkali stress.

### Osmotic stress may play an important role in salt, alkali and salt-alkali stress

Osmotic stress is one of the major factor participating in salt and alkali induced stress pathways [58, 59]. In this study, the osmolality of solutions for alkali, salt and salt-alkali treatments increased was significantly higher than the control, osmotic stress may be involved in all of the stressful treatments. Alkaline or saline stress-induced activation of osmotic signaling acts possibly through both ABA-dependent and ABA-independent pathways [60–62]. We found that the expression of several ABA signaling related genes was lower in salt or alkali sensitive hybrids, whereas some other genes were expressed higher in salt–alkali sensitive hybrids (Fig. 3a-c). Osmotic stress can also cause the accumulation of secondary metabolites and reactive oxygen species (ROS) [63]. The transcription of genes related to secondary metabolites were upregulated in salt and alkali sensitive hybrids (Fig. 3a and c). Cell wall biosynthesis and membrane maintaining related protein can limit cellular ROS concentration under salt stress [64]. We also found higher expression of a large proportion of cell wall associated genes in alkali and salt–alkali sensitive hybrids (Fig. 3b and c).

### GAP models can be cost-efficient tools for large-scale breeding

In this study, 17 and 13 QTLs were identified for SID and AID; this is consistent with the results from cereal crops [18, 44]. The effects of markers designed on the QTL intervals varied from 0.0354 to 0.4235 for SID and from 0.0119 to 0.2134 for AID. By estimating the genotype effects of each marker, GAP models were developed for SID, AID, and SAID to calculate GPV and to select potentially elite individuals from a hybrid population. The prediction accuracy was relatively high compared with GS for fruit quality traits in apple [42, 65, 66]. In contrast, GAP models had a low efficiency for selection of salt–alkali tolerant apple hybrids (Table 2) because there were only 29 hybrids, which SAID less than 0.32 in the population and 26 of them were selected accounted for 89.7% (Table S20). The unique advantage of these GAP models was attributed to the integration of significant QTLs with GS strategy by estimation of marker genotype effects [37, 38, 67]. The prediction accuracy for SAID was relatively low because there were only two markers used in the model. Additional QTLs and markers should be identified for SAID in the future research.

To increase the predictability of the GAP models, in this study, candidate genes were predicted within the significant intervals of QTLs assisted by parental resequencing and RNA-seq data [35, 36, 43]. The markers were designed on the CDS, promoter, or the near intergenic regions of candidate genes. By using this strategy, the linkage disequilibrium decay of these QTL-based markers may be effectively eliminated [38, 68]. Thus the prediction accuracy of the GAP models was relatively high. However, the genetic variations on only two of these candidate genes, *MdRGLG3* and *MdKCAB*, were experimentally validated, a lot of work should be carried out onwards.

### The *MdRGLG3* SNP182G conferred tolerance to salt, alkali, and salt–alkali stress

JA and salicylate (SA) are core micro-molecules involved in biotic and abiotic stress responses [69–71]. In *Arabidopsis*, *RGLG3*, a ring-type ubiquitin ligase gene, functions as an essential negative regulator of JA signaling in response to SA, taking a pivotal role in SA-JA antagonism [72, 73]. An allelic variation in *MdRGLG3* due to SNP182G altered an amino acid in the vWFA-domain and led to an increased tolerance to salt, alkali, and salt–alkali stress in F1 hybrids of ‘BC’ × ‘M9’ apple rootstock. This functional variation was confirmed by transgenic apple calli. Similarly in *Arabidopsis*, *rglg3 rglg4* double mutant represses wound-stunted growth and relative gene expressions [72]. We also found that MdRGLG3-RNAi apple calli were sensitive to salt, alkali, and salt–alkali stress conditions. The genotype frequency of *MdRGLG3* SNP182G:G was as low as 1% in 100 *Malus* accessions, suggesting a possibly lethal effect of the SNP182G:G genotype.

### SNP11G and SNP761A of *MdKCAB* contributed to salt, alkali, and salt–alkali stress tolerance

Voltage-gated potassium channels are complexes of membrane-bound, ion-conducting alpha and cytoplasmic ancillary KCAB subunits. KCAB interacts with specific alpha-subunits to modify their expression or kinetics [74]. A limited number of studies have been conducted on plant voltage-gated K+ channels; therefore, knowledge about these potassium channels are lagging far behind similar studies in animals [75]. We found that the linked allelic variations of *MdKCAB* SNP11G and SNP761A could alter an amino acid residue at the Kv_beta domain of the peptide, contributing to an increase in salt, alkali, and salt–alkali tolerance in both apple plants and transgenic calli. These results may encourage future research into this mechanism.

## Conclusions

A total of 17, 13, and two QTLs for SID, AID, and SAID, respectively, were identified. The genotype effects of these QTL-based markers were estimated and GPVs were predicted using additive GAP models. The prediction accuracy was 0.6569, 0.6695, and 0.5834 for SID, AID, and SAID, respectively. Two functional markers were developed, i.e. SNP182G on *MdRGLG3* and SNP11G/SNP761A on *MdRGLG3,* which conferred tolerance to salt, alkali, and salt–alkali stress. The QTLs and the functional variations in candidate genes may facilitate understanding the mechanism of abiotic stress tolerance. The GAP models can potentially assist future molecular breeding in woody perennials. Still more functional markers should be developed in the onward study to improve the prediction accuracy of the GAP models.

## Methods

### Plant materials and treatments

All the plant materials were originally possessed by China Agricultural University. The experimental research on plants including field investigation and sample collection were performed under institutional guidelines in accordance with local legislation. A total of 3258 hybrids were used in this study. These hybrids were derived from a cross between the salt and alkali tolerant apple rootstock ‘Baleng Crab (BC)’(*Malus robusta* Rehd.) and the salt and alkali sensitive ‘M9’ (*M. pumila* Mill.). From 2015 to 2017, at least 36 plants from each hybrid were propagated via leafy cutting and were sand-cultured. The plants were drip irrigated with 1/2 Hoagland’s solution at a flow rate of 2 L·h^− 1^. Salt, alkali, and salt–alkali stress treatments were performed for 30 days with three biological replicates (three plants per hybrid per treatment or control). The salt treatment consisted of the addition of 100 mM NaCl to ½ Hoagland’s solution; the alkali treatment consisted of Na_2_CO_3_ until pH = 9.0. The salt–alkali stress treatment consisted of the addition of both 100 mM NaCl and Na_2_CO_3_ until pH = 9.0 to the nutrient solution [76, 77]. The osmolality of the nutrient solutions was measured with Split type hygrometer (GM1361, Benetech), following the formula described previously by other authors [78]. To examine the genotype frequency of candidate genes, 100 *Malus* accessions were genotyped using a KASP assay. The severity of salt, alkali, and salt–alkali injury was scored as previously described (Fig. 1) [76]. The tolerance was negatively represented by SID, AID, and SAID.

### BSA-seq analysis

Segregant bulks of each phenotype consisted of 11–26 hybrids, the hybrids extremely tolerant or extremely sensitive to salt, alkali, or salt–alkali stress, respectively. Genomic DNA of each hybrid was extracted, equally pooled, and sequenced using a paired-ends 150 strategy (Illumina X10, Illumina, USA). The sequencing reads were obtained and mapped to the apple ‘Golden Delicious’ dihaploid GDDH genome using Burrows-Wheeler Alignment software [79, 80]. A modified G’ value was used for the statistical analysis of allelic variations between the two bulks [43, 81].

### RNA-seq analysis

Three hybrids that were randomly chosen from each of the extremely tolerant/sensitives bulks were used for RNA-seq. In the salt and alkali stress treatments, root samples were collected at 0, 2, 4, and 6 days after treatment. For the combined salt–alkali stress treatment, root samples were collected 0, 1, 2, and 3 days after treatment, because under salt-alkali stress condition, the injury symptom appeared much early. Three plants of non-treated hybrids were also sampled as a control. Total RNA was extracted using a modified CTAB method [82]. An RNA-seq library was constructed using NEBNext Poly(A) mRNA Magnetic Isolation Module and NEBNext Ultra Directional RNA Library Prep Kit for Illumina (New England Biolabs) following the manufacturer’s protocols. The library was sequenced (paired-end 150) with the Illumina HiSeq X Ten platform. The RNA-seq reads were mapped to the apple ‘Golden Delicious’ dihaploid GDDH genome with HISAT2 [83]. StringTie was used to assembly and quantify transcripts [84]. The co-expression network of the DEGs was analyzed using AppleMDO webtools [85].

### Gene relative expression assay

Primers for real-time quantitative PCR (RT-qPCR) were designed and are listed in Table S25. RT-qPCR was performed using SYBR premix Ex Taq (TaKaRa) with an ABI7500 RT PCR system (Applied Biosystems). β-ACTIN was used as an internal reference.

### KASP genotyping, marker effect estimation, and GAP modeling

Three hundred randomly chosen F1 hybrids from ‘BC’ × ‘M9’ were genotyped for each marker using the KASP assay (LGC Genomics, Beverly, MA, USA). The parents, ‘BC’ and ‘M9,’ were used as a control. The KASP primers were designed based on the 200 bp sequence flanking the SNPs. The genotype data were output via ‘SNP VIEWER’ software (LGC). The genotype effect of a marker was estimated by subtracting the mean phenotype value of the population from the average phenotype value of individuals with a certain genotype. The marker effect was calculated by the absolute genotype effect deviation of a marker.

To develop GAP models, the GPV of a certain hybrid was estimated by adding the sum of genotype effects of all markers to the mean phenotype value of the population [86]. The GAP models were then subjected to five-fold cross validation [42, 67]. The prediction accuracy of GAP models was determined by Pearson’s correlation coefficients and linear regressions between GPV and OPV.

### Validation of genetic variations in candidate genes

Genomic DNA was extracted from young leaves of the two parents using an improved CTAB method [87]. PCR was performed with high-fidelity Platinum TaqDNA Polymerase (Thermo Fisher Scientific), using specific primers for cloning promoter regions (Table S25). The PCR amplified fragments were sequenced by Invitrogen Biotechnology Co., Ltd. (Shanghai, http://www.invitrogen.com.cn). The sequences were mapped to the apple ‘Golden Delicious’ dihaploid GDDH genome and the loci showing variation were confirmed by sequence comparison.

### Generation of transgenic apple calli and salt, alkali, and salt–alkali treatments

*Agrobacterium tumefaciens* EHA105 plasmid was transformed into ‘Orin’ apple callus following a previously published protocol [88]. ‘Orin’ apple callus was grown on an Murashige and Skoog medium containing 1.0 mg/L 2,4-dichlorophenoxyacetic acid and 1.0 mg/L 6-benzylamino purine in the dark at 25 °C, and sub-cultured at 15 d intervals. A seven-day-old callus grown in a liquid medium was used transformed by infection with *Agrobacterium* for 10 min with gentle rotation at 25 °C. After 2 days of co-cultivation with *Agrobacterium*, the callus was transferred to a subculture medium supplemented with 200 mg/L cefotaxime and 30 mg/L kanamycin for transgene selection. For the salt stress treatment, NaCl (100 mmol/L) was added to the callus culture medium. Na_2_CO_3_ (pH = 9.0) was added to the culture medium for the alkali stress treatment. For the combined salt–alkali stress treatment NaCl + Na_2_CO_3_ (100 mmol/L, pH = 9.0) were added to the culture medium. The control consisted of the callus grown on culture medium without any additions [89]. Fourteen days after treatment, the fresh weight of the calli was weighed and the MDA content was assayed following a previously described method [90].

### Statistical analysis

Data are shown with values expressed as means plus or minus standard deviations (SD). Statistical differences were determined using Student’s t-tests. Differences were considered significant at *P* < 0.05 (*), *P* < 0.01 (**), and *P* < 0.001(***).

## Supplementary information

**Additional file 1. **Comparison in genomic DNA sequences of the upstream prior to the ATG codon of *MdRGLG3* in apple rootstocks *Malus robusta* Rehd. ‘Baleng Crab (BC)’ × *M. pumila* Mill. ‘M9’.

**Additional file 2. **Comparison in genomic DNA sequences of the upstream prior to the ATG codon of *MdKCAB* in apple rootstocks *Malus robusta* Rehd. ‘Baleng Crab (BC)’ × *M. pumila* Mill. ‘M9’.

**Additional file 3: Supplementary Table S1.** The phenotype data of alkali injury index (AID) of hybrids derived from ‘Baleng Crab’ (*Malus robusta*) × ‘M9’ (*M. pumila*) in 2015–2017. **Supplementary Table S2.** The phenotype data of salt injury index (SID) of hybrids derived from ‘Baleng Crab’ (*Malus robusta*) × ‘M9’ (*M. pumila*) in 2015–2017. **Supplementary Table S3.** The phenotype data of salt-alkali injury index (SAID) of hybrids derived from ‘Baleng Crab’ (*Malus robusta*) × ‘M9’ (*M. pumila*) in 2016–2017. **Supplementary Table S4.** Phenotypes of extremity segregant bulks for injury indices of salt, alkali, and salt-alkali in hybrids derived from ‘Baleng Crab’ (*Malus robusta*) × ‘M9’ (*M. pumila*) **Supplementary Table S5.** Summary of the BSA-seq data in extremity bulks of tolerant and sensitive using hybrids derived from ‘Baleng Crab’ (*Malus robusta*) × ‘M9’ (*M. pumila*). **Supplementary Table S6.** Summary of QTLs for injury indices of salt, alkali, and salt-alkali identified via BSA-seq in hybrid population from ‘Baleng Crab’ (*Malus robusta*) × ‘M9’ (*M. pumila*). **Supplementary Table S7.** Candidate genes for salt, alkali, and salt-alkali tolerance screened by parental re-sequencing, BSA-seq, and RNA-seq in a hybrid population from ‘Baleng Crab’ (*Malus robusta*) × ‘M9’ (*M. pumila*). **Supplementary Table S8.** Summary of candidate genes for salt, alkali, and salt-alkali tolerance screened by parental re-sequencing, BSA-seq, and RNA-seq in a hybrid population from ‘Baleng Crab’ (*Malus robusta*) × ‘M9’ (*M. pumila*). **Supplementary Table S9.** samples of root tissue for RNA-seq taken from salt, alkali, and salt-alkali treated hybrids from ‘Baleng Crab’ (*Malus robusta*) × ‘M9’ (*M. pumila*). **Supplementary Table S10.** Summary of the sequencing reads and reads mapping in RNA-seq.

**Additional file 4: Supplementary Table S11.** Sample correlation analysis of three biological replicates in RNA-seq. **Supplementary Table S12.** Differentially expressed genes (DEGs) detected by DESeq2 methods (FDR < 0.05, log2FC > 1 or log2FC < − 1,verticals represent normal genes. Up and down arrows represent up and down) in RNA-seq of salt. **Supplementary Table S13.** Differentially expressed genes (DEGs) detected by DESeq2 methods (FDR < 0.05, log2FC > 1 or log2FC < − 1, verticals represent normal genes. Up and down arrows represent up and down) in RNA-seq of alkali. **Supplementary Table S14.** Differentially expressed genes (DEGs) detected by DESeq2 methods (FDR < 0.05, log2FC > 1 or log2FC < − 1,verticals represent normal genes. Up and down arrows represent up and down) in RNA-seq of salt-alkali. **Supplementary Table S15.** Co-expression network identified by AppleMDO analysis tools in hybrids tolerant or sensitive to salt, alkali, and salt-alkali treatments in hybrids from *Malus robusta* Rehd. ‘Baleng Crab’ × *M. pumila* Mill. ‘M9’. **Supplementary Table S16.** Data for allelic variations of markers using for KASP assay in hybrids from ‘Baleng Crab’ (*Malus robusta*) × ‘M9’ (*M. pumila*). **Supplementary Table S17.** Estimates of marker genotype effects on salt, alkali, and salt-alkali injury indices in hybrids from ‘Baleng Crab’ (*Malus robusta*) × ‘M9’ (*M. pumila*). **Supplementary Table S18.** The marker genotypes, genomics predicted values (GPV), and observed phenotype values (OPV) of alkali injury index in 299 F1 hybrids from ‘Baleng Crab’ (*Malus robusta*) × ‘M9’ (*M. pumila*) . **Supplementary Table S19.** The marker genotypes, genomics predicted values (GPV), and observed phenotype values (OPV) of salt injury index in 285 F1 hybrids from ‘Baleng Crab’ (*Malus robusta*) × ‘M9’ (*M. pumila*). **Supplementary Table S20.** The marker genotypes, genomics predicted values (GPV), and observed phenotype values (OPV) of saline-alkali injury index in 194 F1 hybrids from ‘Baleng Crab’ (*Malus robusta*) × ‘M9’ (*M. pumila*)

**Additional file 5: Supplementary Table S21.** The five-fold cross validation of genomics assisted prediction accuracy for injury indices of salt, alkali, and salt-alkali in F1 hybrids from ‘Baleng Crab’ (*Malus robusta*) × ‘M9’ (*M. pumila*). **Supplementary Table S22.** Linkage analysis of MdRGLG3 SNP168, SNP182, SNP932, and SNP936 with KASP marker on chromosome 16 of alkali injury index. **Supplementary Table S23.** Linkage analysis of MdKCAB SNP11 and SNP761 with KASP marker on chromosome 16 of salt-alkali injury index. **Supplementary Table S24.** Genotypes of MdRGLG3 SNP168, SNP182, SNP932, and SNP936 in 100 *Malus* germplasm accessions. **Supplementary Table S25.** Primers of experimental validation in this study

**Additional file 6: Fig. S1.** Frequency distributions of salt (A), alkali (B), salt-alkali (C) injury indices in apple rootstock F1 hybrids of *Malus robusta* Rehd. ‘Baleng Crab’ × *M. pumila* Mill. ‘M9’ in 2015–2017.

**Additional file 7: Fig. S2.** Diagrams showing quantitative trait loci (QTLs) for salt injury index identified using bulked segregant analysis by sequencing in F1 hybrids of apple rootstocks *Malus robusta* Rehd. ‘Baleng Crab (BC)’ × *M. pumila* Mill. ‘M9’. Y-axis represents the G’ value, X-axis represents chromosome physical position. The red curved lines: ‘M9’, blue curved lines: ‘BC’, black curved lines: ‘BC’ & ‘M9’. The horizontal lines with colors indicate the corresponding statistic significant threshold of G’ value.

**Additional file 8: Fig. S3.** Diagrams showing quantitative trait loci (QTLs) for alkali injury index identified using bulked segregant analysis by sequencing in F1 hybrids of apple rootstocks *Malus robusta* Rehd. ‘Baleng Crab (BC)’ × *M. pumila* Mill. ‘M9’. Y-axis represents G’ value, X-axis represents chromosome physical position. The red curved lines: ‘M9’, blue curved lines: ‘BC’, black curved lines: ‘BC’ & ‘M9’. The horizontal lines with colors indicate the corresponding statistic significant threshold of G’ value.

**Additional file 9: Fig. S4.** Diagrams showing quantitative trait loci (QTLs) for salt-alkali injury index identified using bulked segregant analysis by sequencing in F1 hybrids of apple rootstocks *Malus robusta* Rehd. ‘Baleng Crab (BC)’ × *M. pumila* Mill. ‘M9’. Y-axis represents G’ value, X-axis represents chromosome physical position. The red curved lines: ‘M9’, blue curved lines: ‘BC’, black curved lines: ‘BC’ & ‘M9’. The horizontal lines with colors indicate the corresponding statistic significant threshold of G’ value.

**Additional file 10: Fig. S5.** Sanger sequencing confirmed allelic variations in the coding region of *MdRGLG3* between *Malus robusta* Rehd. ‘Baleng Crab (BC)’ × *M. pumila* Mill. ‘M9’.

**Additional file 11: Fig. S6.** Dynamic changes in expression of *MdRGLG3* (A) and *MdKCAB* (B) in salt, alkali and salt-alkali tolerant (T) or sensitive (S) hybrids of apple rootstocks *Malus robusta* Rehd. ‘Baleng Crab’ × *M. pumila* Mill. ‘M9’. The gene expressions were shown in fragments per kilobase per million (FPKM) by RNA-seq and relative expression by qPCR.

**Additional file 12: Fig. S7.** Sanger sequencing confirmed allelic variations in the coding region of *MdKCAB* between *Malus robusta* Rehd. ‘Baleng Crab (BC)’ × *M. pumila* Mill. ‘M9’.

**Additional file 13: Fig. S8.** Differences in osmolality in nutrient solutions used for alkaline (Na_2_CO_3_), saline (NaCl), and alkaline-saline (NaCl + Na_2_CO_3_) stress treatments. Data are means of three biological replicates. Error bars indicate standard deviations. ** and **** indicate statistically significant differences at, *P* < 0.01, and *P* < 0.0001, respectively.

## Data Availability

All RNA-seq reads, DNA re-sequencing reads and BSA-seq data are freely available and have been deposited in the NCBI Sequence Read Archive with the accession number PRJNA645374 (https://www.ncbi.nlm.nih.gov/sra/?term=PRJNA645374). The apple ‘Golden Delicious’ dihaploid GDDH genome (ftp://ftp.bioinfo.wsu.edu/species/Malus_x_domestica/Malus_x_domestica-genome_GDDH13_v1.1/assembly/) is also available at Genome Database for Rosaceae.

## Abbreviations

ABA: Abscisic acid
AID: Alkali injury index
BSA-seq: Bulked segregant analysis via next generation resequencing
FPKM: Fragments per kilobase per million
GAP: Genomics-assisted prediction
GPV: Genomics predicted value
GS: Genomic selection
JA: Jasmonate
KASP: Kompetitive allele-specific PCR
MAS: Marker assisted selection
MDA: Malondialdehyde
OPV: Observed phenotype value
qPCR: Real-time quantitative PCR
QTLs: Quantitative trait loci
SA: Salicylate
SAID: Salt-alkali injury index
SID: Salt injury index
SNP: Single nucleotide polymorphism
WT: Wild type
