# Supplementary material for: Genomics-assisted prediction of salt and alkali tolerances and functional marker development in apple rootstocks
Source: BMC Genomics. 2020 Aug 10;21:550. doi: 10.1186/s12864-020-06961-9 (PMC7430842; doi:10.1186/s12864-020-06961-9)
Supplement: Supplementary file 1 — Additional file 1. Comparison in genomic DNA sequences of the upstream prior to the ATG codon of MdRGLG3 in apple rootstocks Malus robusta Rehd. ‘Baleng Crab (BC)’ × M. pumila Mill. ‘M9’. [file 12864_2020_6961_MOESM1_ESM.pdf]

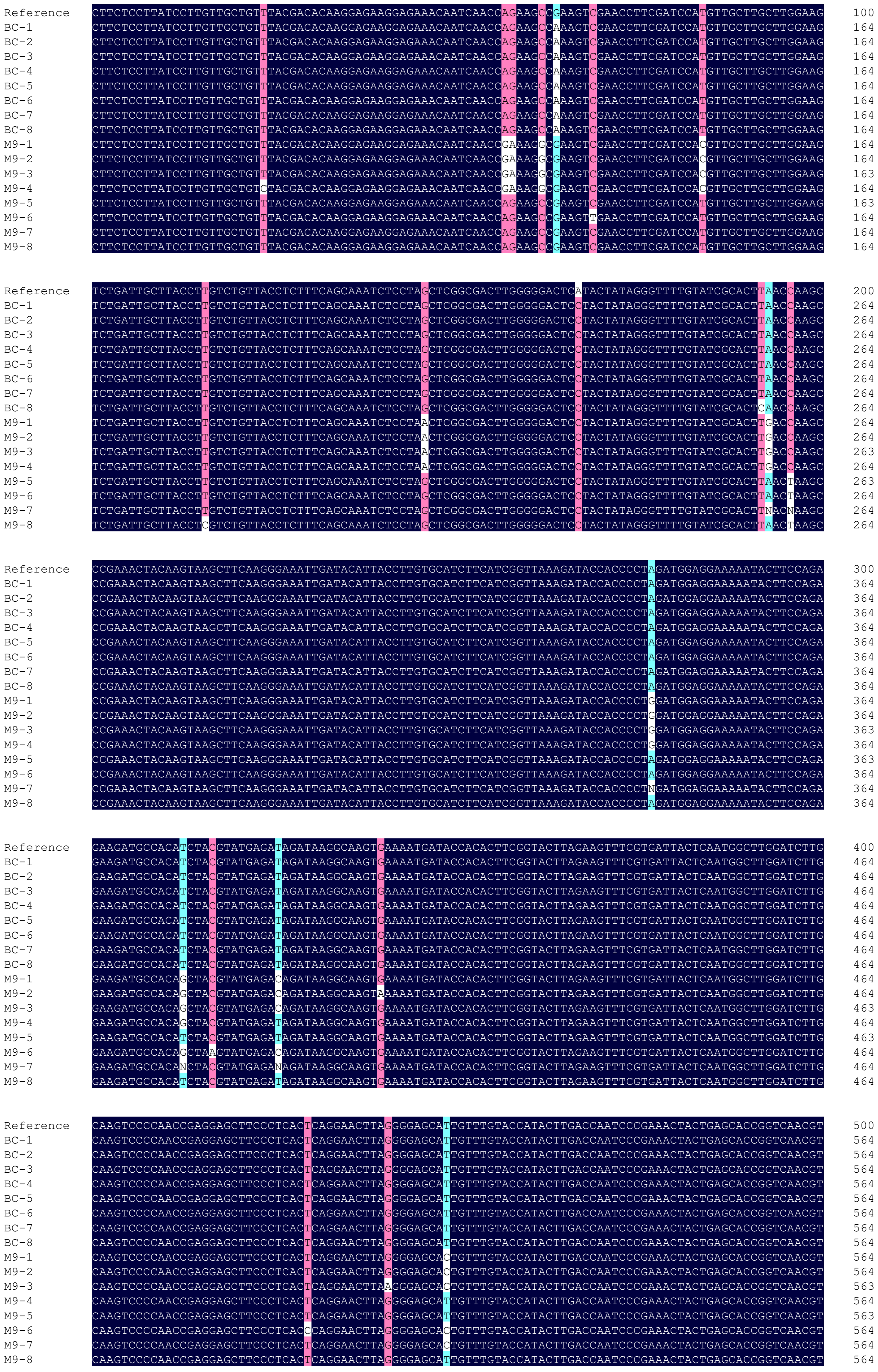
Reference
BC-1
BC-2
BC-3
BC-4
BC-5
BC-6
BC-7
BC-8
M9-1
M9-2
M9-3
M9-4
M9-5
M9-6
M9-7
M9-8

Reference	TATACCGTCAAGGACCCAAAAGAGTTTACCTCCAACCAGGAGGCCAATCACAGCGCGACACGTGTCGATATCAGAAACCAATCATAGCGCGACACGTGTC	600
BC-1	TATACCGTCAAGGACCCAAAAGAGTTTACCTCCAACCAGGAGGCCAATCACAGCGCGACACGTGTCGATATCAGAAACCAATCATAGCGCGACACGTGTC	664
BC-2	TATACCGTCAAGGACCCAAAAGAGTTTACCTCCAACCAGGAGGCCAATCACAGCGCGACACGTGTCGATATCAGAAACCAATCATAGCGCGACACGTGTC	664
BC-3	TATACCGTCAAGGACCCAAAAGAGTTTACCTCCAACCAGGAGGCCAATCACAGCGCGACACGTGTCGATATCAGAAACCAATCATAGCGCGACACGTGTC	664
BC-4	TATACCGTCAAGGACCCAAAAGAGTTTACCTCCAACCAGGAGGCCAATCACAGCGCGACACGTGTCGATATCAGAAACCAATCATAGCGCGACACGTGTC	664
BC-5	TATACCGTCAAGGACCCAAAAGAGTTTACCTCCAACCAGGAGGCCAATCACAGCGCGACACGTGTCGATATCAGAAACCAATCATAGCGCGACACGTGTC	664
BC-6	TATACCGTCAAGGACCCAAAAGAGTTTACCTCCAACCAGGAGGCCAATCACAGCGCGACACGTGTCGATATCAGAAACCAATCATAGCGCGACACGTGTC	664
BC-7	TATACCGTCAAGGACCCAAAAGAGTTTACCTCCAACCAGGAGGCCAATCACAGCGCGACACGTGTCGATATCAGAAACCAATCATAGCGCGACACGTGTC	664
BC-8	TATACCGTCAAGGACCCAAAAGAGTTTACCTCCAACCAGGAGGCCAATCACAGCGCGACACGTGTCGATATCAGAAACCAATCATAGCGCGACACGTGTC	664
M9-1	TATATCGTCAAGGACCCAAAAGAGTTTCCCTCTAACCAGGAGGCCAATCACAGCGCGACACGTGTCGATATCAGAAACCAATCATAGCGCGACACGTGTC	664
M9-2	TATATCGTCAAGGACCCAAAAGAGTTTCCCTCTAACCAGGAGGCCAATCACAGCGCGACACGTGTCGATATCAGAAACCAATCATAGCGCGACACGTGTC	664
M9-3	TATATCGTCAAGGACCCAAAAGAGTTTCCCTCTAACCAGGAGGCCAATCACAGCGCGACACGTGTCGATATCAGAAACCAATCATAGCGCGACACGTGTC	663
M9-4	TATACCGTCAAGGACCCAAAAGAGTTTACCTCCAACCAGGAGGCCAATCACAGCGCGACACGTGTCGATATCAGAAACCAATCATAGCGCGACACGTGTC	664
M9-5	TATACCGTCAAGGACCCAAAAGAGTTTACCTCCAACCAGGAGGCCAATCACAGCGCGACACGTGTCGATATCAGAAACCAATCATAGCGCGACACGTGTC	663
M9-6	TATATCGTCAAGGACCCAAAAGAGTTTCCCTCCAACCAGGAGGCCAATCACAGCGCGACACGTGTCGATATCAGAAACCAATCATAGCGCGACACGTGTC	664
M9-7	TATANCGTCAAGGACCCAAAAGAGTTTNCCTCNANCCAGGAGGCCAANCACAGCGCGACACGTGTCGATATCAGAAACCAATCATAGCGCGACACGTGTC	664
M9-8	TATACCGTCAAGGACCCAAAAGAGTTTACCTCCAACCAGGAGGCCAATCACAGCGCGACACGTGTCGATATCAGAAACCAATCATAGCGCGACACGTGTC	664

Reference	AACATCAGAAGCCAATCACAACACGACACATGTCAATGTCAGAATGAAACTAGAAATTCTCTTCTATAAATAGAGATCATTCTCTTACAATATTTCCTAA	700
BC-1	AACATCAGAAGCCAATCACAACACGACACATGTCAATGTCAGAATGAAACTAGAAATTCTCTTCTATAAATAGAGATCATTCTCTTACAATATTTCCTAA	764
BC-2	AACATCAGAAGCCAATCACAACACGACACATGTCAATGTCAGAATGAAACTAGAAATTCTCTTCTATAAATAGAGATCATTCTCTTACAATATTTCCTAA	764
BC-3	AACATCAGAAGCCAATCACAACACGACACATGTCAATGTCAGAATGAAACTAGAAATTCTCTTCTATAAATAGAGATCATTCTCTTACAATATTTCCTAA	764
BC-4	AACATCAGAAGCCAATCACAACACGACACATGTCAATGTCAGAATGAAACTAGAAATTCTCTTCTATAAATAGAGATCATTCTCTTACAATATTTCCTAA	764
BC-5	AACATCAGAAGCCAATCACAACACGACACATGTCAATGTCAGAATGAAACTAGAAATTCTCTTCTATAAATAGAGATCATTCTCTTACAATATTTCCTAA	764
BC-6	AACATCAGAAGCCAATCACAACACGACACATGTCAATGTCAGAATGAAACTAGAAATTCTCTTCTATAAATAGAGATCATTCTCTTACAATATTTCCTAA	764
BC-7	AACATCAGAAGCCAATCACAACACGACACATGTCAATGTCAGAATGAAACTAGAAATTCTCTTCTATAAATAGAGATCATTCTCTTACAATATTTCCTAA	764
BC-8	AACATCAGAAGCCAATCACAACACGACACATGTCAATGTCAGAATGAAACTAGAAATTCTCTTCTATAAATAGAGATCATTCTCTTACAATATTTCCTAA	764
M9-1	AACATCAGTAGTCAATCACAACACGACACGTGTCAATGTCAGAATGAAACTAGAAACTCTCTTCTATAAATAGAGATCATTCTCTTACAATATTTCCTAA	764
M9-2	AACATCAGTAGTCAATCACAACACGACACGTGTCAATGTCAGAATGAAACTAGAAACTCTCTTCTATAAATAGAGATCATTCTCTTACAATATTTCCTAA	764
M9-3	AACATCAGTAGTCAATCACAACACGACACGTGTCAATGTCAGAATGAAACTAGAAACTCTCTTCTATAAATAGAGATCATTCCCTTACAATATTTCCTAA	763
M9-4	AACATCAGAAGCCAATCACAACACGACACATGTCAATGTCAGAATGAAACTAGAAATTCTCTTCTATAAATAGAGATCATTCTCTTACAATATTTCCTAA	764
M9-5	AACATCAGAAGCCAATCACAACACGACACATGTCAATGTCAGAATGAAACTAGAAATTCTCTTCTATAAATAGAGATCATTCTCTTACAATATTTCCTAA	763
M9-6	AACATCAGTAGTCAATCACAACACGACACGTGTCAATGTCAGAATGAAACTAGAAACTCTCTTCTATAAATAGAGATCATTCTCTTACAATATTTCCTAA	764
M9-7	AACATCAGNAGNCAATCANAACACGACACNTGTCAATGTCAGAATGAAACTAGAAANTCTCTTCTATAAATAGAGATCATTCTCTTACAATATTTCCTAA	764
M9-8	AACATCAGAAGCCAATCACAACACGACACATGTCAATGTCAGAATGAAACTAGAAATTCTCTTCTATAAATAGAGATCATTCTCTTACAATATTTCCTAA	764

Reference	TGCTATTTGTACTAAATCATTCACTAGTACTCACTAAATGAGAGCTTGAACCTATGTACTTGTGTAAACCCTTCACAATTAATGAGAACTCCTCTAATTT	800
BC-1	TGCTATTTGTACTAAATCATTCACTAGTACTCACTAAATGAGAGCTTGAACCTATGTACTTGTGTAAACCCTTCACAATTAATGAGAACTCCTCTAATTT	864
BC-2	TGCTATTTGTACTAAATCATTCACTAGTACTCACTAAATGAGAGCTTGAACCTATGTACTTGTGTAAACCCTTCACAATTAATGAGAACTCCTCTAATTT	864
BC-3	TGCTATTTGTACTAAATCATTCACTAGTACTCACTAAATGAGAGCTTGAACCTATGTACTTGTGTAAACCCTTCACAATTAATGAGAACTCCTCTAATTT	864
BC-4	TGCTATTTGTACTAAATCATTCACTAGTACTCACTAAATGAGAGCTTGAACCTATGTACTTGTGTAAACCCTTCACAATTAATGAGAACTCCTCTAATTT	864
BC-5	TGCTATTTGTACTAAATCACTCACTAGTACTCACTAAATGAGAGCTTGAACCTATGTACTTGTGTAAACCCTTCACAATTAATGAGAACTCCTCTAATTT	864
BC-6	TGCTATTTGTACTAAATCATTCACTAGTACTCACTAAATGAGAGCTTGAACCTATGTACTTGTGTAAACCCTTCACAATTAATGAGAACTCCTCTAATTT	864
BC-7	TGCTATTTGTACTAAATCATTCACTAGTACTCACTAAATGAGAGCTTGAACCTATGTACTTGTGTAAACCCTTCACAATTAATGAGAACTCCTCTAATTT	864
BC-8	TGCTATTTGTACTAAATCATTCACTAGTACTCACTAAATGAGAGCTTGAACCTATGTACTTGTGTAAACCCTTCACAATTAATGAGAACTCCTCTAATTT	864
M9-1	TGTTATTTGTGCTAAATCATTCACTAGTACTCACTAAATGAGAGCTTGAACCTATGTACTTGTGTAAACCCTTCACAATTAATGAGAACTCCTCTAATTT	864
M9-2	TGTTATTTGTGCTAAATCATTCACTAGTACTCACTAAATGAGAGCTTGAACCTATGTACTTGTGTAAACCCTTCACAATTAATGAGAACTCCTCTAATTC	864
M9-3	TGTTATTTGTGCTAAATCATTCACTAGTACTCACTAAATGAGAGCTTGAACCTATGTACTTGTGTAAACCCCTCACAATTAATGAGAACTCCTCTAATTC	863
M9-4	TGCTATTTGTACTAAATCATTCACTAGTACTCACTAAATGAGAGCTTGAACCTATGTACTTGTGTAAACCCTTCACAATTAATGAGAACTCCTCTAATTT	864
M9-5	TGCTATTTGTACTAAATCATTCACTAGTACTCACTAAATGAGAGCTTGAACCTATGTACTTGTGTAAACCCTTCACAATTAATGAGAACTCCTCTAATTT	863
M9-6	TGCTATTTGTACTAAATCATTCACTAGTACTCACTAAATGAGAGCTTGAACCTATGTACTTGTGTAAACCCTTCACAATTAATGAGAACTCCTCTAATTT	864
M9-7	TGTTATTTGTGCTAAATCATTCACTAGTACTCACTAAATGAGAGCTTGAACCTATGTACTTGTGTAAACCCTTCACAATTAATGAGAACTCCTCTAATTC	864
M9-8	TGCTATTTGTACTAAATCATTCACTAGTACTCACTAAATGAGAGCTTGAACCTATGTACTTGTGTAAACCCTTCACAATTAATGAGAACTCCTCTAATTT	864

Reference	GTGGACGTAGCC.AATCTGAGTGAACCACGTACATCTTGTGTTTGCTTCCCTATCTCTATCCACTTACATACTTATCCACACTAGTGACCGGAGCAATCT	899
BC-1	GTGGACGTAGCC.AATCTGAGTGAACCACGTACATCTTGTGTTTGCTTCCCTATTTCTATCCACTTACATACTTATCCACACTAGTGACCGGAGCAATCT	963
BC-2	GTGGACGTAGCC.AATCTGAGTGAACCACGTACATCTTGTGTTTGCTTCCCTATTTCTATCCACTTACATACTTATCCACACTAGTGACCGGAGCAATCT	963
BC-3	GTGGACGTAGCC.AATCTGAGTGAACCACGTACATCTTGTGTTTGCTTCCCTATTTCTATCCACTTACATACTTATCCACACTAGTGACCGGAGCAATCT	963
BC-4	GTGGACGTAGCCCAATCTGAGTGAACCACGTACATCTTGTGTTTGCTTCCCTATTTCTATCCACTTACATACTTATCCACACTAGTGACCGGAGCAATCT	964
BC-5	GTGGACGTAGCCCAATCTGAGTGAACCACGTACATCTTGTGTTTGCTTCCCTATTTCTATCCACTTACATACTTATCCACACTAGTGACCGGAGCAATCT	964
BC-6	GTGGACGTAGCC.AATCTGAGTGAACCACGTACATCTTGTGTTTGCTTCCCTATTTCTATCCACTTACATACTTATCCACACTAGTGACCGGAGCAATCT	963
BC-7	GTGGACGTAGCC.AATCTGAGTGAACCACGTACATCTTGTGTTTGCTTCCCTATTTCTATCCACTTACATACTTATCCACACTAGTGACCGGAGCAATCT	963
BC-8	GTGGACGTAGCC.AATCTGAGTGAACCACGTACATCTTGTGTTTGCTTCCCTATTTCTATCCACTTACATACTTATCCACACTAGTGACCGGAGCAATCT	963
M9-1	GTGGACGTAGCC.AATCTGAGTGAACCACGTACATCTTGTGTTTGCTTCCCTATCTCTATCCACTTACATACTTATCCACACTAGTGACCGGAGCAATCT	963
M9-2	GTGGACGTAGCC.AATCTGGGTGAACCACGTACATCTTGTGTTTGCTTCCCTGTCTCTATCCACTTACATACTTATCCACACTAGTGACTGGAACAATCT	963
M9-3	GTGGACGTAGCC.AATCTGGGTGAACCACGTACATCTTGTGTTTGCTTCCCTGTCTCTATCCACTTACATACTTATCCACACTAGTGACTGGAACAATCT	962
M9-4	GTGGACGTAGCC.AATCTGAGTGAACCACGTACATCTTGTGTTTGCTTCCCTATCTCTATCCACTTACATACTTATCCACACTAGTGACCGGAGCAACCT	963
M9-5	GTGGACGTAGCC.AATCTGAGTGAACCACGTACATCTTGTGTTTGCTTCCCTATCTCTATCCACTTACATACTTATCCACACTAGTGACCGGAGCAATCT	962
M9-6	GTGGACGTAGCC.AATCTGAGTGAACCACGTACATCTTGTGTTTGCTTCCCTATCTCTATCCACTTACATACTTATCCACACTAGTGACTGGAACAATCT	963
M9-7	GTGGACGTAGCC.AATCTGGGTGAACCACGTACATCTTGTGTTTGCTTCCCTGTCTCTATCCACTTACATACTTATCCACACTAGTGACTGGAACAATCT	963
M9-8	GTGGACGTAGCC.AATCTGAGTGAACCACGTACATCTTGTGTTTGCTTCCCTATCTCTATCCACTCACATACTTATCCACACTAGTGACCGGAGCAATCT	963

Reference	AGCAAAGGTCACAAACTTAACACTTTCTGTTGTACCAAAGTCCTCGCTGATTTTGTGCATCAACAATATCAACCGTTGATCTGAGATCAACCGTTGATAT	999
BC-1	AGCAAAGGTCACAAACTTAACACTTTCTGTTGTACCAAAGTCCTCGCTGATTTTGTGCATCAACAATATCAACCGTTGATCTGAGATCAACCGTTGATAT	1063
BC-2	AGCAAAGGTCACAAACTTAACACTTTCTGTTGTACCAAAGTCCTCGCTGATTTTGTGCATCAACAATATCAACCGTTGATCTGAGATCAACCGTTGATAT	1063
BC-3	AGCAAAGGTCACAAACTTAACACTTTCTGTTGTACCAAAGTCCTCGCTGATTTTGTGCATCAACAATATCAACCGTTGATCTGAGATCAACCGTTGATAT	1063
BC-4	AGCAAAGGTCACAAACTTAACACTTTCTGTTGTACCAAAGTCCTCGCTGATTTTGTGCATCAACAATATCAACCGTTGATCTGAGATCAACCGTTGATAT	1064
BC-5	AGCAAAGGTCACAAACTTAACACTTTCTGTTGTACCAAAGTCCTCGCTGATTTTGTGCATCAACAATATCAACCGTTGATCTGAGATCAACCGTTGATAT	1064
BC-6	AGCAAAGGTCACAAACTTAACACTTTCTGTTGTACCAAAGTCCTCGCTGATTTTGTGCATCAACAATATCAACCGTTGATCTGAGATCAACCGTTGATAT	1063
BC-7	AGCAAAGGTCACAAACTTAACACTTTCTGTTGTACCAAAGTCCTCGCTGATTTTGTGCATCAACAATATCAACCGTTGATCTGAGATCAACCGTTGATAT	1063
BC-8	AGCAAAGGTCACAAACTTAACACTTTCTGTTGTACCAAAGTCCTCGCTGATTTTGTGCATCAACAATATCAACCGTTGATCTGAGATCAACCGTTGATAT	1063
M9-1	AGCAAAGGTCACAAACTTAACACTTTCTGTTGTACCAAAGTCCTCGCTGATTTTGTGCATCAACAATATCAACCGTTGATCTGAGATCAACCGTTGATAT	1063
M9-2	AGCAAAGGTTTT...TTGAACACTTTCTGTTGTACCAAAGTCCTCGTTGATTTTGTGCATCAACAATATCAACCGTTGATCTGCGATCAACCGTTGATAT	1060
M9-3	AGCAAAGGTTTT...TTGAACACTTTCTGTTGTACCAAAGTCCTCGTTGATTTTGTGCATCAACAATATCAACCGTTGATCTGCGATCAACCGTTGATAT	1059
M9-4	AGCAAAGGTCACAAACTTAACACTTTCTGTTGTACCAAAGTCCTCGCTGATTTTGTGCATCAACAATATCAACCGTTGATCTGAGATCAACCGTTGATAT	1063
M9-5	AGCAAAGGTCACAAACTTAACACTTTCTGTTGTACCAAAGTCCTCGCTGATTTTGTGCATCAACAATATCAACCGTTGATCTGAGATCAACCGTTGATAT	1062
M9-6	AGCAAAGGTTTT...TTGAACACTTTCTGTTGTGCCAAAGTCCTCGTTGATTTTGTGCATCAACAATATCAACCGTTGATCTGCGATCAACCGTTGATAT	1060
M9-7	AGCAAAGGTTTT...TTGAACACTTTCTGTTGTACCAAAGTCCTCGTTGATTTTGTGCGTCAACAATATCAACCGTTGATCTGCGATCAACCGTTGATAT	1060
M9-8	AGCAAAGGTCACAAACTTAACACTTTCTGTTGTATCAAAGTCCTCGCTGATTTTGTGCATCAACAATATCAACCGTTGATCTGAGATCAACCGTTGATAT	1063

```
Reference   TAAATAAGATTTTTTTTTATCGTTGCAAATTTGACGGTCCACATTTTCTTGCCGTTGAGATCCAACGGTAACAATTTGAAACTGCGCTGCGGGCCTCATT   1099
BC-1        TAAATAAGATTTTTTTTTATCGTTGCAAATTTGACGGTCCACATTTTCTTGCCGTTGAGATCCAACGGTAACAATTTGAAACTGCGCTGCGGGCCTCATT   1163
BC-2        TAAATAAGATTTTTTTTTATCGTTGCAAATTTGACGGTCCACATTTTCTTGCCGTTGAGATCCAACGGTAACAATTTGAAACTGCGCTGCGGGCCTCATT   1163
BC-3        TAAATAAGATTTTTTTTTATCGTTGCAAATTTGACGGTCCACATTTTCTTGCCGTTGAGATCCAACGGTAACAATTTGAAACTGCGCTGCGGGCCTCATT   1163
BC-4        TAAATAAGATTTTTTTTTATCGTTGCAAATTTGACGGTCCACATTTTCTTGCCGTTGAGATCCAACGGTAACAATTTGAAACTGCGCTGCGGGCCTCATT   1164
BC-5        TAAATAAGATTTTTTTTTATCGTTGCAAATTTGACGGTCCACATTTTCTTGCCGTTGAGATCCAACGGTAACAATTTGAAACTGCGCTGCGGGCCTCATT   1164
BC-6        TAAATAAGATTTTTTTTTATCGTTGCAAATTTGACGGTCCACATTTTCTTGCCGTTGAGATCCAACGGTAACAATTTGAAACTGCGCTGCGGGCCTCATT   1163
BC-7        TAAATAAGATTTTTTTTTATCGTTGCAAATTTGACGGTCCACATTTTCTTGCCGTTGAGATCCAACGGTAACAATTTGAAACTGCGCTGCGGGCCTCATT   1163
BC-8        TAAATAAGATTTTTTTTTATCGTTGCAAATTTGACGGTCCACATTTTCTTGCCGTTGAGATCCAACGGTAACAATTTGAAACTGCACTGCGGGCCTCATT   1163
M9-1        TAAATAAGATTTTTTTTTATTGTTGCAAATCTGACGGTCCACATTTTCTTGCCGTTGAGATCCAACGGTAACAATTTGAAACTGCGCTGCGGGCCTCATT   1163
M9-2        TAAATAAGATTTTTTTT.ATTGTTGCAAATCTGACGGTCCACATTTTCTTGCCGTTGAGATCCAACGGTAACAATTTGAAACTGCGCTGCGGGCCTCATT   1159
M9-3        TAAATAAGATTTTTTTT.ATTGTTGCAAATCTGACGGTCCACATTTTCTTGCCGTTGAGATCCAACGGTAACAATTTGAAACTGCGCTGCGGGCCTCATT   1158
M9-4        TAAATAAGATTTTTTTTTATCGTTGCAAATTTGACGGTCCACATTTTCTTGCCGTTGAGATCCAACGGTAACAATTTGAAACTGCGCTGCGGGCCTCATT   1163
M9-5        TAAATAAGATTTTTTTTTATCGTTGCAAATTTGACGGTCCACATTTTCTTGCCGTTGAGATCCAACGGTAACAATTTGAAACTGCGCTGCGGGCCTCATT   1162
M9-6        TAAATAAGATTTTTTTT.ATTGTTGCGAATCTGACGGTCCACATTTTCTTGCCGTTGAGATCCAACGGTAACAATTTGAAACTGCGCTGCGGGCCTCATT   1159
M9-7        TAAATAAGATTTTTTTT.ATTGTTGCAAATCTGACGGTCCACATTTTCTTGCCGTTGAGATCCAACGGTAACAATTTGAAACTGCGCTGCGGGCCTCATT   1159
M9-8        TAAATAAGATTTTTTTTTATCGTTGCAAATTTGACGGTCCACATTTTCTTGCCGTTGAGATCCAACGGTAACAATTTGAAACTGCGCTGCGGGCCTCATT   1163

Reference   CATTGAAAACATGTCGGGTGACGCGCCCCCACAGGCGGGTGCAGTGCACGAGCCTAACAAAAAAAAATTAAAAAAGGGGCTGACGTCAGCCTTGCGTCAG   1199
BC-1        CATTGAAAACATGTCGGGTGACGCGCCCCCACAGGCGGGTGCAGTGCACGAGCCTAACAAAAAAAAATTAAAAAAGGGGCTGACGTCAGCCTTGCGTCAG   1263
BC-2        CATTGAAAACATGTCGGGTGACGCGCCCCCACAGGCGGGTGCAGTGCACGAGCCTAACAAAAAAAAATTAAAAAAGGGGCTGACGTCAGCCTTGCGTCAG   1263
BC-3        CATTGAAAACATGTCGGGTGACGCGCCCCCACAGGCGGGTGCAGTGCACGAGCCTAACAAAAAAAAATTAAAAAAGGGGCTGACGTCAGCCTTGCGTCAG   1263
BC-4        CATTGAAAACATGTCGGGTGACGCGCCCCCACAGGCGGGTGCAGTGCACGAGCCTAACAAAAAAAAATTAAAAAAGGGGCTGACGTCAGCCTTGCGTCAG   1264
BC-5        CATTGAAAACATGTCGGGTGACGCGCCCCCACAGGCGGGTGCAGTGCACGAGCCTAACAAAAAAAAATTAAAAAAGGGGCTGACGTCAGCCTTGCGTCAG   1264
BC-6        CATTGAAAACATGTCGGGTGACGCGCCCCCACAGGCGGGTGCAGTGCACGAGCCTAACAAAAAAAAATTAAAAAAGGGGCTGACGTCAGCCTTGCGTCAG   1263
BC-7        CATTGAAAACATGTCGGGTGACGCGCCCCCACAGGCGGGTGCAGTGCACGAGCCTAACAAAAAAAAATTAAAAAAGGGGCTGACGTCAGCCTTGCGTCAG   1263
BC-8        CATTGAAAACATGTCGGGTGACGCGCCCCCACAGGCGGGTGCAGTGCACGAGCCTAACAAAAAAAAATTAAAAAAGGGGCTGACGTCAGCCTTGCGTCAG   1263
M9-1        CGTTAAAAACATGTCGGGTGACGTGCCCCCACAGGCGAGTGCAGTGCACGCGCCTGACAAAAAAAAATAAAAAAAGGGGCTGACGTCAGCCTTGCGTCAG   1263
M9-2        CGCTAAAAACATGTCGAGTGACGTGCCCCCACAGGCGAGTGCAGTGCACGCGCCTGGCAAAAAAAAATAAAAAAAGGGGCTGACGTCAGCCTTGCGTCAG   1259
M9-3        CGTTAAAAACATGTCGGGTGACGTGCCCCCACAGGCGAGTGCAGTGCACGCGCCTGACAAAAAAAA.TAAAAAAAGGGGCTGACGTCAGCCTTGCGTCAG   1257
M9-4        CATTGAAAACACGTCGGGTGACGCGCCCCCACAGGCGGGTGCAGTGCACGAGCCTAACAAAAAAAAATTAAAAAAGGGGCTGACGTCAGCCTTGCGTCAG   1263
M9-5        CATTGAAAACATGTCGGGTGACGCGCCCCCACAGGCGGGTGCAGTGCACGAGCCTAACAAAAAAAAATTAAAAAAGGGGCTGACGTCAGCCTTGCGTCAG   1262
M9-6        CGTTAAAAACATGTCGGGTGACGTGCCCCCACAGGCGAGTGCAGTGCACGCGCCTGACAAAAAAAAATAAAAAAAGGGGCTGACGTCAGCCTTGCGTCAG   1259
M9-7        CGTTAAAAACATGTCGGGTGACGTGCCCCCACAGGCGAGTGCAGTGCACGCGCCTGACAAAAAAAAATAAAAAAAGGGGCTGACGTCAGCCTTGCGTCAG   1259
M9-8        CATTGAAAACATGTCGGGTGACGCGCCCCCACAGGCGGGTGCAGTGCACGAGCCTAACAAAAAAAAATTAAAAAAGGGGCTGACGTCAGCCTTGCGTCAG   1263

Reference   ATAGCCTTCGGGCTCACGGGCTGGCAATTAATGACATGCCTGGTGCTTGAGCATGTCCAATCCCTCGGGCTCCCACACGTTGGAGTCAATAGGCCGGGCT   1299
BC-1        ATAGCCTTCGGGCTCACGGGCTGGCAATTAATGACATGCCTGGTGCTTGAGCATGTCCAATCCCTCGGGCTCCCACACGTTGGAGTCAATAGGCCGGGCT   1363
BC-2        ATAGCCTTCGGGCTCACGGGCTGGCAATTAATGACATGCCTGGTGCTTGAGCATGTCCAATCCCTCGGGCTCCCACACGTTGGAGTCAATAGGCCGGGCT   1363
BC-3        ATAGCCTTCGGGCTCACGGGCTGGCAATTAATGACATGCCTGGTGCTTGAGCATGTCCAATCCCTCGGGCTCCCACACGTTGGAGTCAATAGGCCGGGCT   1363
BC-4        ATAGCCTTCGGGCTCACGGGCTGGCAATTAATGACATGCCTGGTGCTTGAGCATATCCAATCCCTCGGGCTCCCACACGTTGGAGTCAATAGGCCGGGCT   1364
BC-5        ATAGCCTTCGGGCTCACGGGCTGGCAATTAATGACATGCCTGGTGCTTGAGCATGTCCAATCCCTCGGGCTCCCACACGTTGGAGTCAATAGGCCGGGCT   1364
BC-6        ATAGCCTTCGGGCTCACGGGCTGGCAATTAATGACATGCCTGGTGCTTGAGCATGTCCAATCCCTCGGGCTCCCACACGTTGGAGTCAATAGGCCGGGCT   1363
BC-7        ATAGCCTTCGGGCTCACGGGCTGGCAATTAATGACATGCCTGGTGCTTGAGCATGTCCAATCCCTCGGGCTCCCACACGTTGGAGTCAATAGGCCGGGCT   1363
BC-8        ATAGCCTTCGGGCTCACGGGCTGGCAATTAATGACATGCCTGGTGCTTGAGCATGTCCAATCCCTCGGGCTCCCACACGTTGGAGTCAATAGGCCGGGCT   1363
M9-1        ATAGCCTTCGGGCTCACGGGCTGGCAATTCATGACATGCCTGGTGCTCGAGCATGTCCAATCCCTCGGGCTCCCACAGGTTGGAGTCAATAGGCCGGGCT   1363
M9-2        ATAGCCTTCGGGCTCACGGGCTGGCAATTCATGACATGCCTGGTGCTCGAGCATGTCCAATCCCTCGGGCTCCCACAGGTTGGAGTCAATAGGCCGGGCT   1359
M9-3        ATAGCCTTCGGGCTCACGGGCTGGCAATTCATGACATGCCTGGTGCTCGAGCATGTCCAATCCCTCGGGCTCCCACAGGTTGGAGTCAATAGGCCGGGCT   1357
M9-4        ATAGCCTTCGGGCTCACGGGCTGGCAATTAATGACATGCCTGGTGCTTGAGCATGTCCAATCCCTCGGGCTCCCACACGTTGGAGTCAATAGGCCGGGCT   1363
M9-5        ATAGCCTTCGGGCTCACGGGCTGGCAATTAATGACATGCCTGGTGCTTGAGCATGTCCAATCCCTCGGGCTCCCACACGTTGGAGTCAATAGGCCGGGCT   1362
M9-6        ATAGCCTTCGGGCTCACGGGCTGGCAATTCATGACATGCCTGGTGCTCGAGCATGTCCAATCCCTCGGGCTCCCACAGGTTGGAGTCAATAGGCCGGGCT   1359
M9-7        ATAGCCTTCGGGCTCACGGGCTGGCAATTCATGACATGCCTGGTGCTCGAGCATGTCCAATCCCTCGGGCTCCCACAGGTTGGAGTCAATAGGCCGGGCT   1359
M9-8        ATAGCCTTCGGGCTCACGGGCTGGCAATTAATGACATGCCTGGTGCTTGAGCATGTCCAATCCCTCGGGCTCCCACACGTTGGAGTCAATAGGCCGGGCT   1363

Reference   CTCGGGCCCTGTTTGTTGGCTTGTCCCCCGAGCTCCAGCCAACGCTGGAAGTGCTCTAATTGATTGTGATAGGGATTTATTACTCGATTGAGTTGATTTT   1399
BC-1        CTCGGGCCCTGTTTGTTGGCTTGTCCCCCGAGCTCCAGCCAACGCTGGAAGTGCTCTAATTGATTGTGATAGGGATTTATTACTCGATTCAGTTGATTTT   1463
BC-2        CTCGGGCCCTGTTTGTTGGCTTGTCCCCCGAGCTCCAGCCAACGCTGGAAGTGCTCTAATTGATTGTGATAGGGATTTATTACTCGATTCAGTTGATTTT   1463
BC-3        CTCGGGCCCTGTTTGTTGGCTTGTCCCCCGAGCTCCAGCCAACGCTGGAAGTGCTCTAATTGATTGTGATAGGGATTTATTACTCGATTCAGTTGATTTT   1463
BC-4        CTCGGGCCCTGTTTGTTGGCTTGTCCCCCGAGCTCCAGCCAACGCTGGAAGTGCTCTAATTGATTGTGATAGGGATTTATTACTCGATTCAGTTGATTTT   1464
BC-5        CTCGGGCCCTGTTTGTTGGCTTGTCCCCCGAGCTCCAGCCAACGCTGGAAGTGCTCTAATTGATTGTGATAGGGATTTATTACTCGATTCAGTTGATTTT   1464
BC-6        CTCGGGCCCTGTTTGTTGGCTTGTCCCCCGAGCTCCAGCCAACGCTGGAAGTGCTCTAATTGATTGTGATAGGGATTTATTACTCGATTCAGTTGATTTT   1463
BC-7        CTCGGGCCCTGTTTGTTGGCTTGTCCCCCGAGCTCCAGCCAACGCTGGAAGTGCTCTAATTGATTGTGATAGGGATTTATTACTCGATTCAGTTGATTTT   1463
BC-8        CTCGGGCCCTGCTTGTTGGCTTGTCCCCCGAGCTCCAGCCAACGCTGGAAGTGCTCTAATTGATTGTGATAGGGATTTATTACTCGATTCAGTTGATTTT   1463
M9-1        CTCGGGCCCTGTTTGTTGGCTTGTCCCCCGAGCTCCAGCCAACGCTGGAAGTGCTCTAATTGATTGTGATAGGGATTTATTACTCGATTGAGTTGATTTT   1463
M9-2        CTCGGGCCCTGTTTGCTGGCTTGTCCCCCGAGCTCCAGCCAACGCTGGAAGTGCTCTAATTGATTGTGATAGGGATTCATTACTCGATTGAGTTGATTTT   1459
M9-3        CTCGGGCCCTGTCTGCTGGCTTGTCCCCCGAGCTCCAGCCAACGCTGGAAGTGCTCTAATTGATTGTGATAGGGATTCATTACTCGATTGAGTTGATTTT   1457
M9-4        CTCGGGCCCTGTTTGTTGGCTTGTCCCCCGAGCTCCAGCCAACGCTGGAAGTGCTCTAATTGATTGTGATAGGGATTTATTACTCGATTGAGTTGATTTT   1463
M9-5        CTCGGGCCCTGTTTGTTGGCTTGTCCCCCGAGCTCCAGCCAACGCTGGAAGTGCTCTAATTGATTGTGATAGGGATTTATTACTCGATTGAGTTGATTTT   1462
M9-6        CTCGGGCCCTGTTTGCTGGCTTGTCCCCCGAGCTCCAGCCAACGCTGGAAGTGCTCTAATTGATTGTGATAGGGATTCATTACTCGATTGGGTTGATTTT   1459
M9-7        CTCGGGCCCTGTTTGCTGGCTTGTCCCCCGAGCTCCAGCCAACGCTGGAGGTGCTCTAATTGATTGTGATAGGGATTCATTACTCGATTGAGTTGATTTT   1459
M9-8        CTCGGGCCCTGTTTGTTGGCTTGTCCCCCGAGCTCCAGCCAACGCTGGAAGTGCTCTAATTGATTGTGATAGGGATTTATTACTAGATTGAGTTGATTTT   1463

Reference   CAATTGAGAATGTATTAAAGATAGGATTTAGTAATTTTTATTATCTTTGACTTTTACATGTCACGAACAGGAGAGTTTGAGCTGTCGTTGTCTGCTTGAA   1499
BC-1        CAATTGAGAATGTATTAAAGATAGGATTTAGTAATTTTTATTATCTTTGACTTTTACATGTCACGAACAGGAGAGTTTGAGCTGTCGTTGTCTGCTTGAA   1563
BC-2        CAATTGAGAATGTATTAAAGATAGGATTTAGTAATTTTTATTATCTTTGACTTTTACATGTCACGAACAGGAGAGTTTGAGCTGTCGTTGTCTGCTTGAA   1563
BC-3        CAATTGAGAATGTATTAAAGATAGGATTTAGTAATTTTTATTATCTTTGACTTTTACATGTCACGAACAGGAGAGTTTGAGCTGTCGTTGTCTGCTTGAA   1563
BC-4        CAATTGAGAATGTATTAAAGATAGGATTTAGTAATTTTTATTATCTTTGACTTTTACATGTCACGAACAGGAGAGTTTGAGCTGTCGTTGTCTGCTTGAA   1564
BC-5        CAATTGAGAGTGTATTAAAGATAGGATTTAGTAATTTTTATTATCTTTGACTTTTACATGTCACGAACAGGAGAGTTTGAGCTGTCGTTGTCTGCTTGAA   1564
BC-6        CAATTGAGAATGTATTAAAGATAGGATTTAGTAATTTTTATTATCTTTGACTTTTACATGTCACGAACAGGAGAGTTTGAGCTGTCGTTGTCTGCTTGAA   1563
BC-7        CAATTGAGAATGTATTAAAGATAGGATTTAGTAATTTTTATTATCTTTGACTTTTACATGTCACGAACAGGAGAGTTTGAGCTGTCGTTGTCTGCTTGAA   1563
BC-8        CAATTGAGAATGTATTAAAGATAGGATTTAGTAATTTTTATTATCTTTGACTTTTACATGTCACGAACAGGAGAGTTTGAGCTGTCGTTGTCTGCTTGAA   1563
M9-1        CAATTGAGAATGTATTAAAGATAGGATTTAGTAATTTTTATTATCTTTGACTTTTACATGTCACGAACAGGAGAGTTTGAGCTGTCGTTGTCTGCTTGAA   1563
M9-2        TAATTGAGAATGTATTAAAGATAGGATTTAGTAATTTTTATTATCTTTGACTTTTACATGTCACGAGCAGGAGAGTTTGAGCTGCCGTTGTCTGCTTGAA   1559
M9-3        TAATTGAGAATGTATTAAAGATAGGATTTAGTAATTTTTATTATCTTTGACTTTTACATGTCACGAACAGGAGAGTTTGAGCTGTCGTTGTCTGCTTGAA   1557
M9-4        CAATTGAGAATGTATTAAAGATAGGATTTAGTAATTTTTATTATCTTTGACTTTTACATGTCACGAACAGGAGAGTTTGAGCTGTCGTTGTCTGCTTGAA   1563
M9-5        CAATTGAGAATGTATTAAAGATAGGATTTAGTAATTTTTATTATCTTTGACTTTTACATGTCACGAACAGGAGAGTTTGAGCTGTCGTTGTCTGCTTGAA   1562
M9-6        CAATTGAGAATGTATTAAAGATAGGATTTAGTAATTTTTATTATCTTTGACTTTTACATGTCACGAACAGGAGAGCTTGAGCTGTCGTTGTCTGCTTGAA   1559
M9-7        TAATTGAGAATGTATTAAAGATAGGATTTAGTAATTTTTATTATCTTTGACTTTTACATGTCACGAACAGGAGAGTTTGAGCTGTCGTTGTCTGCTTGAA   1559
M9-8        CAATTGAGAATGTATTAAAGATAGGATTTAGTAATTTTTATTATCTTTGACTTTTACATGTCACGAACAGGAGAGTTTGAGCTGTCGTTGTCTGCTTGAA   1563
```

```
Reference    CCTTCATGATGCTAAGCTGCCCGTGAGAACTATTGAGGGTCCTGTCCATTTAACGAGGGAGACAAAGAAGGAGACAAAGGGTAAACAACAACGGCT.CTC    1598
BC-1         CCTTCAGGATGCTAAGCTGCCCGTGAGAACTATTGAGGGTCCTGTCCATTTAACGAGGGAGACAAAGAAGGAGACAAAGGGTAAACAACAACGGCT.CTC    1662
BC-2         CCTTCAGGATGCTAAGCTGCCCGTGAGAACTATTGAGGGTCCTGTCCATTTAACGAGGGAGACAAAGAAGGAGACAAAGGGTAAACAACAACGGCT.CTC    1662
BC-3         CCTTCAGGATGCTAAGCTGCCCGTGAGAACTATTGAGGGTCCTGTCCATTTAACGAGGGAGACAAAGAAGGAGACAAAGGGTAAACAACAACGGCT.CTC    1662
BC-4         CCTTCAGGATGCTAAGCTGCCCGTGAGAACTATTGAGGGTCCTGTCCATTTAACGAGGGAGACAAAGAAGGAGACAAAGGGTAAACAACAACGGTT.CTC    1663
BC-5         CCTTCAGGATGCTAAGCTGCCCGTGAGAACTATTGAGGGTCCTGTCCATTTAACGAGGGAGACAAAGAAGGAGACAAAGGGTAAACAACAACGGCT.CTC    1663
BC-6         CCTTCAGGATGCTAAGCTGCCCGTGAGAACTATTGAGGGTCCTGTCCATTTAACGAGGGAGACAAAGAAGGAGACAAAGGGTAAACAACAACGGCT.CTC    1662
BC-7         CCTTCAGGATGCTAAGCTGCCCGTGAGAACTATTGAGGGTCCTGTCCATTTAACGAGGGAGACAAAGAAGGAGACAAAGGGTAAACAACAACGGCT.CTC    1662
BC-8         CCTTCAGGATGCTAAGCTGCCCGTGAGAACTATTGAGGGTCCTGTCCATTTAACGAGGGAGACAAAGAAGGAGACAAAGAGTAAACAGCGACGGCT.CTC    1662
M9-1         CCTTCAGGATGCTAAGCTGCCCGTGAGAACTATTGAGGGTCCTGTCCATTTAACGAGGGAGACAAAGAAGGAGACAAAGGGTAAACAACAACGGCT.CTC    1662
M9-2         CCTTCAGGATGCTAAGCTGCCCGTGAGAACTATTGAGGGTCCTGTCCATTTAACGAGGGAGACGAAGAAGGAGACAAAGGGTAAACAACGACGGCT.CTC    1658
M9-3         CCTTCAGGATGCTAAGCTGCCCGTGAGAACTATTGAGGGTCCTGTCCATTTAACGAGGGAGACAAAGAAGGAGACAAAGGGTAAACAACGACGGCTACTC    1657
M9-4         CCTTCAGGATGCTAAGCTGCCCGTGAGAACTATTGAGGGTCCTGTCCATTTAACGAGGGAGACAAAGAAGGAGACAAAGGGTAAACAACGACGGCT.CTC    1662
M9-5         CCTTCAGGATGCTAAGCTGCCCGTGAGAACTATTGAGGGTCCTGTCCATTTAACGAGGGAGACAAAGAAGGAGACAAAGGGTAAACAACAACGGCT.CTC    1661
M9-6         CCTTCAGGATGCTAAGCTGCCCGTGAGAACTATTGAGGGTCCTGTCCATTTAACGAGGGAGACAAAGAAAGAGACAAAGGGTAAACAACAACGGCT.CTC    1658
M9-7         CCTTCAGGATGCTAAGCTGCCCGTGAGAACTATTGAGGGTCCTGTCCATTTAACGAGGGAGACAAAGAAGGAGACAAAGGGTAAACAACGACGGCT.CTC    1658
M9-8         CCTTCAGGATGCTAAGCTGCCCGTGAGAACTATTGAGGGTCCTGTCCATTTAACGAGGGAGACAAAGAAGGAGACAGAGGGTAAACAACAACGGCT.CTC    1662

Reference    AC    1600
BC-1         AC    1664
BC-2         AC    1664
BC-3         AC    1664
BC-4         AC    1665
BC-5         AC    1665
BC-6         AC    1664
BC-7         AC    1664
BC-8         AC    1664
M9-1         AC    1664
M9-2         AC    1660
M9-3         AC    1659
M9-4         AC    1664
M9-5         AC    1663
M9-6         AC    1660
M9-7         AC    1660
M9-8         AC    1664
```
