# Supplementary material for: Genomics-assisted prediction of salt and alkali tolerances and functional marker development in apple rootstocks
Source: BMC Genomics. 2020 Aug 10;21:550. doi: 10.1186/s12864-020-06961-9 (PMC7430842; doi:10.1186/s12864-020-06961-9)
Supplement: Supplementary file 2 — Additional file 2. Comparison in genomic DNA sequences of the upstream prior to the ATG codon of MdKCAB in apple rootstocks Malus robusta Rehd. ‘Baleng Crab (BC)’ × M. pumila Mill. ‘M9’. [file 12864_2020_6961_MOESM2_ESM.pdf]

Reference	TCATACATCCGTTAAAGTTGCTGTTAAATCAGCTGTTAACTGGTGACGTGACATCCACGTGGACAATGATTGGGCACCGCGTGTCAATACGGACCCACGT	100
BC-1	TCATACATCCGTTAAAGTTGCTGTTAAATCAGCTGTTAACTGGTGACGTGACATCCACGTGGACAATGATTGGGCACCGCGTGTCAATACGGACCCACGT	102
BC-2	TCATACATCCGTTAAAGTTGCTGTTAAATCAGCTGTTAACTGGTGACGTGACATCCACGTGGACAATGATTGGGCACCGCGTGTCAATACGGACCCACGT	102
BC-3	TCATACATCCGTTAAAGTTGCTGTTAAATCAGCTGTTAACTGGTGACGTGACATCCACGTGGACAATGATTGGGCACCGCGTGTCAATACGGACCCACGT	128
BC-4	TCATACATCCGTTAAAGTTGCTGTTAAATCAGCTGTTAACTGGTGACGTGACATCCACGTGGACAATGATTGGGCACCGCGTGTCAATACGGACCCACGT	113
BC-5	TCATACATCCGTTAAAGTTGCTGTTAAATCAGCTGTTAACTGGTGACGTGACATCCACGTGGACAATGATTGGGCACCGCGTGTCAATACGGACCCACGT	119
BC-6	TCATACATCCGTTAAAGTTGCTGTTAAATCAGCTGTTAACTGGTGACGTGACATCCACGTGGACAATGATTGGGCACCGCGTGTCAATACGGACCCACGT	119
BC-7	TCATACATCCGTTAAAGTTGCTGTTAATTCAGCTGTTAACTGGTGACGTGACATCCACGTGGACAATGATTGGGCACCGCGTGTCAATACGGACCCACGT	119
BC-8	TCATACATCCGTTAAAGTTGCTGTTAAATCAGCTGTTAACTGGTGACGTGACATCCACGTGGACAATGATTGGGCACCGCGTGTCAATACGGACCCACGT	119
M9-1	TCATACATCCGTTAAAGTTGCTGTTAAATCAGCTGTTAACTGGTGACGTGACATCCACGTGGACAATGATTGGGCACCGCGTGTCAATACGGACCCACGT	119
M9-2	TCATACATCCGTTAAAGTTGCTGTTAAATCAGCTGTTAACTGGTGACGTGACATCCACGTGGACAATGATTGGGCACCGCGTGTCAATACGGACCCACGT	119
M9-3	TCATACATCCGTTAAAGTTGCTGTTAAATCAGCTGTTAACTGGTGACGTGACATCCACGTGGACAATGATTGGGCACCGCGTGTCAATACGGACCCACGT	119
M9-4	TCATACGTCCGTTAAAGTTGCTGTTAAATCAGCTGTTAACTGGTGACGTGACATCCACGTGGACAATGATTGGGCACCGCGTGTCAATACGGACCCACGT	119
M9-5	TCATACATCCGTTAAAGTTGCTGTTAAATCAGCTGTTAACTGGTGACGTGACATCCACGTGGACAATGATAGGGCACCGCGTGTCAATACGGACCCACGT	119
M9-6	TCATACATCGGTTAAAGTTGCTGTTAAATCAGCTGTTAACTGGCGACGTGACATCCACGTGGACAATAATTGGGCACCGCGTGTCAATACGGACCCACGT	119
M9-7	TCATACTTCCGTTAAAGTTGCTGTTAATTCAGCTGTTGACTGGTGACGTGACATCCACGTGGACAATGATTGGGCACCGCGTGTCAATACGGACCCACGT	119
M9-8	TCATACATCCGTTAAAGTTGCTGTTAAATCAGCTGTTAACTGGTGACGTGACATCCACGTGGACAATGATTGGGCACCGCGTGTCAATACGGACCCACGT	119

Reference	GAATATTAAAAAAATTATAAAATAATAATATTTAAAAAAAAAACAACCTTGGGTCATCTCCTACCTCCCTCGATCCTCCCTCACTCCCTTGATCCCTTCC	200
BC-1	GAATATTAAAAAAATTATAAAATAATAATATTTAAAAAAAAAACAACCTTGGGTCATCTCCTACCTCCCTCGATCCTCCCTCACTCCCTTGATCCCTTCC	202
BC-2	GAATATTAAAAAAATTATAAAATAATAATATTTAAAAAAAAAACAACCTTGGGTCATCTCCTACCTCCCTCGATCCTCCCTCACTCCCTTGATCCCTTCC	202
BC-3	GAATATTAAAAAAATTATAAAATAATAATATTTAAAAAAAAAACAACCTTGGGTCATCTCCCACCTCCCTCGATCCTCCCTCACTCCCTTGATCCCTTAC	228
BC-4	GAATATTAAAAAAATTATAAAATAATAATATTTAAAAAAAAAACAACCTTGGGTCATCTCCTACCTCCCTCGATCCTCCCTCACTACCTTGATCCCTTCC	213
BC-5	GAATATTAAAAAAATTATAAAATAATAATATTTAAAAAAAAAACAACCTTGGGTCATCTCCTACCTCCCTCGATCCTCCCTCACTCCCTTGATCCCTTCC	219
BC-6	GAATATTAAAAAAATTATAAAATAATCATATTTAAAAAAAAAACAACCTTGGGTCATCTCCTACCTCCCTCGATCCTCCCTCACTCCCTTGATCCCTTCC	219
BC-7	GAATATTAAAAAAATTATAAAATAATAATATTTAAAAAAAAAACAACCTTGGGTCATCTCCTACCTCCCTCGATCCTCCCTCACTCCCTTGATCCCTTCC	219
BC-8	GAATGTTAAAAAAATTATAAAATAATAATATTTAAAAAAAAAACAACCTTGGGTCATCTCCTACCTCCCTCGATCCTCCCTCACTCCCTTGATCCCTTCC	219
M9-1	GAATATTAAAAAAATTATAAAATAATAATATTTAAAAAAAAAACAACCTTGGGTCATCTCCCACCTCCCTCGATCCTCCCTCACTCCCTTGATCCCTTCC	219
M9-2	GAATATTAAAAAAATTATAAAATAATAATATTTAAAAAAAAAACAACCTTGGGTCATCTCCTACCTCCCTCGATCCTCCCTCACTCCCTTGATCCCTTCC	219
M9-3	GAATATTAAAAAAATTATAAAATAATAATATTTAAAAAAAAAACAACCTTGGGTCATCTCCCACCTCCCTCGATCCTCCCTCACTCCCTTGATCCATTAC	219
M9-4	GAATATTAAAAAAATTATAAAATAATAATATTTAAAAAAAAAACAACCTTGGGTCATCTCCTACCTCCCTCGATCCTCCCTCACTACCTTGATCCCTTCC	219
M9-5	GAATTTTAAAAAAATTATAAAATAATAATATTTAAAAAAAAAACAACCTTGGGTCATCTCCTACCTCCCTCGATCCTCCCTCACTCCCTTGATCCCTTCC	219
M9-6	GAATATTAAAAAAATTATAAAATAATCATATTTAAAAAAAAAACAACCTTGGGTCATCTCCTACCTCCCTCGATCCTCCCTCACTCCCTTGATCCCTTCC	219
M9-7	GAATATTAAAAAAATTATAAAATAATAATATTTAAAAAAAAAACAACGTTGGGTCATCTCCTACCTCCCTCGATCCTCCCTCACTCCCTTGATCCCTTCC	219
M9-8	GAATGTTAAAAAAATTATAAAATAATAATATTTAAAAAAAAAACAACCTTGGGTCATCTCCTACCTCCCTCGATCCTCCCTCACTCCCTTGATCCCTTCC	219

Reference	TCCACCTCCTCCCTATCCTCCACCATCTTCATCTCCCCTCCCTTCTCTCTCCTCAGATCAACCCAATTTAAAATTTAAAATTTAAAAACCTAAAAGCAAA	300
BC-1	TCCACCTCCTCCCTATCCTCCACCATCTTCATCTCCCCTCCCTTCTCTCTCCTCAGATCAACCCAATTTAAAATTTAAAATTTAAAAACCTAAAAGCAAA	302
BC-2	TCCACATCCTCCCTATCCTCCACCATCTTCATCTCCCCTCCCTTCTCTCTCATCAGATCAACCCAATTTAAAATTTAAAATTTAAAAACCTAAAAGCAAA	302
BC-3	TCCACCTCCTCCCTATCCTCCACCATCTTCATCTCCCCTCCCTTCTCTCTCCTCAGATCAACCCAATTTAAAATTTAAAATTTAAAAACCTAAAAGCAAA	328
BC-4	TCCACCTCCTCCCTATCCTCCACCATCTTCATCTCCCCTCCCTTCTCTCTCCTCAGATCAACCCAATTTAAAATTTAAAATTTAAAAACCTAAAAGCAAA	313
BC-5	TCCACTTCCTCCCTATCCTCCACCATCTTCATCTCCCCTCCCTTCTCTCTCCTCCGATCAACCCAATTTAAAATTTAAAATTTAAAAACCTAAAAGCAAA	319
BC-6	TCCACCTCCTCCCTATCCTCCACCATCTTCATCTCCCCTCCCTTCTCTCTCCTCAGATCAACCCAATTTAAAATTTAAAATTTAAAAACCTAAAAGCAAA	319
BC-7	TCCACCTCCTCCCTATCCTCCACCATCTTCATCTCCCCTCCCTTCTCTCTCCTCAGATCAACCCAATTTAAAATTTAAAATTTAAAAACCTAAAAGCAAA	319
BC-8	TCCACCTCCTCCCTATCCTCCACCATCTTCATCTCCCCTCCCTTCTCTCTCCTCAGATCAACCCAATTTAAAATTTAATATTTAAAAACCTAAAAGCAAA	319
M9-1	TCCACCTCCTCCCTATCCTCCACCATCTTCATCTCCGCTCCCTTCTCTCTCCTCAGATCAACCCAATTTAAAATTTAAAATTGAAAAACCTAAAAGCATA	319
M9-2	TCCACATCCTCCCTATCCTCCACCATCTTCATCTCCCCTCCCTACTCTCTCATCAGATCAACCCAATTTAAAATTTAAAATTTAAAACCCTAAAAGCAAA	319
M9-3	TCCACCTCCTCCCTATCCTCCACCATCTTCATCTCCCCTCCCTTCTCTCTCCTCAGATCAACCCAATTTAAAATTTAAAATTTAAAAACCTAAAAGCAAA	319
M9-4	TCCACCTCCTCCCTATCCTCCACCATCTTCATCTCCCCTCCCTTCTCTCTCCTCAGATCAACCCAATTTAAAATTTAAAATTTAAAAACCTAAAAGCAAA	319
M9-5	TCCACTTCCTCCCTATCCTCCACCATCTTCATCTCCCCTCCCTTCTCTCTCCTCCGATCAACCCAATTTAAAATTTAAAATTTAAAAACCTAAAAGCAAA	319
M9-6	TCCACCTCCTCCCTATCCTCCACCATCTTCATCTCCCCTCCCTTCTCTCTCCTCAGATCAACCCAATTTAAAATTTAAAATTTAAAAACCTAAAAGCAAA	319
M9-7	TCCACCTCCTCCCTATCCTCCACCATCTTCATCTCCCCTCCCTTCTCTCTCCTCAGATCAACCCAATTTAAAATTTAAAATTTAAAAACCTAAAAGCAAA	319
M9-8	TCCACCTCCTCCCTATCCTCCACCATCTTCATCTCCCCTCCCTTCTCTCTCCTCAGATCAACCCAATTTAAAATTTAATATTTAAAAACCTAAAAGCAAA	319

Reference	AACCCCAACCTGACGCCCAAATTATCACTTTGAAGGCTTGGAAGGCAGAAGGGTTGCTGGACTTGACCGCCAAAGAAATTGATGAGCTGCTGGGCAACCA	400
BC-1	AACCCCAACCTGACGCCCAAATTATCACTTTGAAGGCTTGGAAGGCAGAAGGGTTGCTGGACTTGACCGCCAAAGAAATTGATGAGCTGCTGGGCAACCA	402
BC-2	AACCCCAACCTGACGCCCAAATTATCACTTTGAAGGCTTGGAAGGCAGAAGGGTTGCTGGACTTGACCGCCAAAGAAATTGATGAGCTGCTGGGCAACCA	402
BC-3	AACCCCAACCTGACGCCCAAATTATCACTTTGAAGGCTTGGAAGGCAGAAGGGTTGCTGGACTTGACCGCCAAAGAAATTGATGAGCTGCTGGGCAACCA	428
BC-4	AACCCCAACCTGACGCCCAAATTATCACTTTGAAGGCTTGGAAGGCAGAAGGGTTGCTGGACTTGACCGCCAAAGAAATTGATGAGCTGCTGGGCAACCA	413
BC-5	AACCCCAACCTGACGCCCAAATTATCACTTTGAAGGCTTGGAAGGCAGAAGGGTTGCTGGACTTGACCGCCAAAGAAATTGATGAGCTGCTGGGCAACCA	419
BC-6	AACCCCAACCTGACGCCCAAATTATCACTTTGAAGGCTTGGAAGGCAGAAGGGTTGCTGGACTTGACCGCCAAAGAAATTGATGAGCTGCTGGGCAACCA	419
BC-7	AACCCCTACCTGACGCCCAAATTATCACTTTGAAGGGTTGGAAGGCAGAAGGGTTGCTGGACTTGACCGCCAAAGAAATTGATGAGCTGCTGGGCAACCA	419
BC-8	AACCCCAACCTGACGCCCAAATTATCACTTTGAAGGCTTGGAAGGCAGAAGGGTTGCTGGACTTGACCGCCAAAGAAATTGATGAGCTGCTGGGCAACCA	419
M9-1	AACCCCAACCTGACGCCCAAATTATCACTTTGAAGGCTTGGAAGGCAGAAGGGTTGCTGGACTTGACCGCCAAAGAAATTGATGAGCTGCTGGGCAACCA	419
M9-2	AACCCCAACCTGACGCCCAAATTATCACTTTGAAGGCTTGGAAGGCAGAAGGGTTGCTGGACTTGACCGCCAAAGAAATTGATGAGCTGCTGGGCAACCA	419
M9-3	AACCCCAACCTGACGCCCAAATTATCACTTTGAAGGCTTGGAAGGCAGAAGGGTTGCTGGACTTGACCGCCAAAGAAATTGATGAGCTGCTGGGCAACCA	419
M9-4	AACCCCAACCTGACGCCCAAATTATCACTTTGAAGGCTTGGAAGGCAGAAGGGTTGCTGGACTTGACCGCCAAAGAAATTGATGAGCTGCTGGGCAACCA	419
M9-5	AACCCCAACCTGACGCCCAAATTATCACTTTGAAGGCTTGGAAGGCAGAAGGGTTGCTGGACTTGACCGCCAAAGAAATTGATGAGCTGCTGGGCAACCA	419
M9-6	AACCCCAACCTGACGCCCAAATTATCACTTTGAAGGCTTGGAAGGCAGAAGGGTTGCTGGACTTGACCGCCAAAGAAATTGATGAGCTGCTGGGCAACCA	419
M9-7	AACCCCTACCTGACGCCCAAATTATCACTTTGAAGGGTTGGAAGGCAGAAGGGTTGCTGGACTTGACCGCCAAAGAAATTGATGAGCTGCTGGGCAACCA	419
M9-8	AACCCCAACCTGACGCCCAAATTATCACTTTGAAGGCTTGGAAGGCAGAAGGGTTGCTGGACTTGACCGCCAAAGAAATTGATGAGCTGCTGGGCAACCA	419

Reference	CGAGGATGTCGTACGCCACCAGAAAAGCAGATCTGCAGCCATGGCTGCTCTTCTAGTTCCTGCTGCTTTAAACCTTCTACATTAGTGTCGTCCTGTGGGT	500
BC-1	CGAGGATGTCGTACGCCACCAGAAAAGCAGATCTGCAGCCATGGCTGCTCTTCTAGTTCCTGCTGCTTTAAACCTTCTACATTAGTGTCGTCCTGTGGGT	502
BC-2	CGAGGATGTCGTACGCCACCAGAAAAGCAGATCTGCAGCCATGGCTGCTCTTCTAGTTCCTGCTGGTTTAAACCTTCTACATTAGTGTCGTCCTGTGGGT	502
BC-3	CGAGGATGTCGTACGCCACCAGAAAAGCAGATCTGCAGCCATGGCTGCTCTTCTAGTTTCTGCTGCTTTAAACCTTCTACATTAGTGTCGTCCTGTGGGT	528
BC-4	CGAGGATGTCGTACGCCACCAGAAAAGCAGATCTGCAGCCATGGCTGCTCTTCTAGTTCCTGCTGCTTTTAACCTTCTACATTAGTGTCGTCCTGTGGGT	513
BC-5	CGAGGATGTCGTACGCCACCAGAAAAGCAGATCTGCAGCCATGGCTGCTCTTCTAGTTCCTGCTGCTTTAAACCTTCTACATTAGTGTCGTCCTGTGGGT	519
BC-6	CGAGGATGTCGTACGCCACCAGAAAAGCAGATCTGCAGCCATGGCTGCTCTTCTAGTTCCTGCTGCTTTAAACCTTCTACATTAGTGTCGTCCTGTGGGT	519
BC-7	CGAGGATGTCGTACGCCACCAGAAAAGCAGATCTGCAGCCATGGCTGCTCTTCTAGTTCCTGCAGCTTTAAACCTTCTACATTAGTGTCGTCCTGTGGGT	519
BC-8	CGAGGATGTCGTACGCCACCAGAAAAGCAGATCTGCAGCCATGGCTGCTCTTCTAGTTCCTGCTGCTTTAAACCTTCTACATTAGTGTCGTCCTGCGGGT	519
M9-1	CGAGGATGTCGTACGCCACCAGAAAAGCAGATCTGCAGCCATGGCTGCTCTTCTAGTTCCTGCTGCTTTAAACCTTCTACATTAGTGTCGTCCTGTGGGT	519
M9-2	CGAGGATGTCGTACGCCACCAGAAAAGCAGATCTGCAGCCATGGCTGCTCTTCTAGTTCCTGCTGGTTTAAACCTTCTACATTAGTGTCGTCCTGTGGGT	519
M9-3	CGAGGATGTCGTACGCCACCAGAAAAGCAGATCTGCAGCCATGGCTGCTCTTCTAGTTTCTGCTGCTTTAAACCTTCTACATTAGTGTCGTCCTGTGGGT	519
M9-4	CGAGGATGTCGTACGCCACCAGAAAAGCAGATCTGCAGCCATGGCTGCTCTTCTAGTTCCTGCTGCTTTTAACCTTCTACATTAGTGTCGTCCTGTGGGT	519
M9-5	CGAGGATGTCGTACGCCACCAGAAAAGCAGATCTGCAGCCATGGCTGCTCTTCTAGTTCCTGCTGCTTTAAACCTTCTACATTAGTGTCGTCCTGTGGGT	519
M9-6	CGAGGATGTCGTACGCCACCAGAAAAGCAGATCTGCAGCCATGGCTGCTCTTCTAGTTCCTGCTGCTTTAAACCTTCTACATTAGTGTCGTCCTGTGGGT	519
M9-7	CGAGGATGTCGTACGCCACCAGAAAAGCAGATCTGCAGCCATGGCTGCTCTTCTAGTTCCTGCAGCTTTAAACCTTCTACATTAGTGTCGTCCTGTGGGT	519
M9-8	CGAGGATGTCGTACGCCACCAGAAAAGCAGATCTGCAGCCATGGCTGCTCTTCTAGTTCCTGCTGCTTTAAACCTTCTACATTAGTGTCGTCCTGCGGGT	519

Reference	TCTGACGACCTTTCTCCTCGATGACCCTGAAATTCCGATGGGGAGGTGGAGGGAGGGATCAGAGGAGTGAGGGTGCAGAGGAGAGAAGGGAGGGAGGGTC	600
BC-1	TCTGACGACGTTTCTCCTCGATGACCCTGAAATTCCGATGGGGAGGTGGAGGGAGGGATCAGAGGAGTGAGGGTGCAGAGGAGAGAAGGGAGGGAGGGTC	602
BC-2	TCTGACGACGTTTCTCCTCGATGACCCTGAAATTCCGATGGGGAGGTGGAGGGAGGGATCAGAGGAGTGAGGGTGCAGAGGAGAGAAGGGAGGGAGGGTC	602
BC-3	TCTGACGACGTTTCTCCTCGATGACCCTGAAATTCCGATGGGGAGGTGGAGGGAGGGATCAGAGGAGTGAGGGTGCAGAGGAGAGAAGGGAGGGAGGGTC	628
BC-4	TCTGACGACGTTTCTCCTCGATGACCCTGAAATTCCGATGGGGAGGTGGAGGGAGGGATCAGAGGAGTGAGGGTGCAGAGGAGAGAAGGGAGGGAGGGTC	613
BC-5	TCTGACGACCTTTCTCCTCGATGACCCTGAAATTCCGATGGGGAGGTGGAGGGAGGGATCAGAGGAGTGAGGGTGCAGAGGAGAGAAGGGAGGGAGGGTC	619
BC-6	TCTGACGACCTTTCTCCTCGATGACCCTGAAATTCCGATGGGGAGGTGGAGGGAGGGATCAGAGGAGTGAGGGTGCAGAGGAGAGAAGGGAGGGAGGGTC	619
BC-7	TCTGACGACCTTTCTCCTCGATGACCCTGAAATTCCGATGGGGAGGTGGAGGGAGGGATCAGAGGAGTGAGGGTGCAGAGGAGAGAAGGGAGGGAGGGTC	619
BC-8	TCTGACGACCTTTCTCCTCGATGACCCTGAAATTCCGATGGGGAGGTGGAGGGAGGGATCAGAGGAGTGAGGGTGCAGAGGAGAGAAGGGAGGGAGGGTC	619
M9-1	TCTGACGACGTTTCTCCTCGATGACCCTGAAATTCCGATGGGGAGGTGGAGGGAGGGATCAGAGGAGTGAGGGTGCAGAGGAGAGAAGGGAGGGAGGGTC	619
M9-2	TCTGACGACGTTTCTCCTCGATGACCCTGAAATTCCGATGGGGAGGTGGAGGGAGGGATCAGAGGAGTGAGGGTGCAGAGGAGAGAAGGGAGGGAGGGTC	619
M9-3	TCTGACGACGTTTCTGCTCGATGACCCTGAAATTCCGATGGGGAGGTGGAGGGAGGGATCAGAGGAGTGAGGGTGCAGAGGAGAGAAGGGAGGGAGGGTC	619
M9-4	TCTGACGACGTTTCTCCTCGATGACCCTGAAATTCCGATGGGGAGGTGGAGGGAGGGATCAGAGGAGTGAGGGTGCAGAGGAGAGAAGGGAGGGAGGGTC	619
M9-5	TCTGACGACCTTTCTCCTCGATGACCCTGAAATTCCGATGGGGAGGTGGAGGGAGGGATCAGAGGAGTGAGGGTGCAGAGGAGAGAAGGGAGGGAGGGTC	619
M9-6	TCTGACGACCTTTCTCCTCGATGACCCTGAAATTCCGATGGGGAGGTGGAGGGAGGGATCAGAGGAGTGAGGGTGCAGAGGAGAGAAGGGAGGGAGGGTC	619
M9-7	TCTGACGACCTTTCTCCTCGATGACCCTGAAATTCCGATGGGGAGGTGGAGGGAGGGATCAGAGGAGTGAGGGTGCAGAGGAGAGAAGGGAGGGAGGGTC	619
M9-8	TCTGACGACCTTTCTCCTCGATGACCCTGAAATTCCGATGGGGAGGTGGAGGGAGGGATCAGAGGAGTGAGGGTGCAGAGGAGAGAAGGGAGGGAGGGTC	619

Reference	GGGGGAGGTATAAGATAACCGAAGTTTTTCTTTTTTGTTTTTAATAATTTTTAATTTTTTTTTAATATTCACGTGGACACATATTGACACGTGATGTCTA	700
BC-1	GGGGGAGGTATAAGATAACCGAAGTTTTTCTTTTTTGTTTTTAATAATTTTTAATTTTTTTTTAATATTCACGTGGACACATATGGACACGTGATGTCTA	702
BC-2	GGGGGAGGTATAAGATAACCGAAGTTTTTCTTTTTTGTTTTTAATAATTTTTAATTTTTTTTTAATATTCACGTGGACACATATGGACACGTGATGTCTA	702
BC-3	GGGGGAGGTATAAGATAACCGAAGTTTTTCTTTTTTGTTTTTAATAATTTTTAATTTTTTTTTAATATTCACGTGGACACATATGGACACGTGATGTCTA	728
BC-4	GGGGGAGGTATAAGATAACCGAAGTTTTTCTTTTTTGTTTTTAATAATTTTTAATTTTTTTTTAATATTCACGTGGACACATATGGACACGTGATGTCTA	713
BC-5	GGGGGAGGTATAAGATAACCGAAGTTTTTCTTTTTTGTTTTTAATAATTTTTAATTTTTTTTTAATATTCACGTGGACACATATTGACACGTGATGTCTA	719
BC-6	GGGGGAGGTATAAGATAACCGAAGTTTTTCTTTTTTGTTTTTAATAATTTTTAATTTTTTTTTAATATTCACGTGGACACATATTGACACGTGATGTCTA	719
BC-7	GGGGGAGGTATAAGATAACCGAAGTTTTTCTTTTTTGTTTTTAATAATTTTTAATTTTTTTTTAATATTCACGTGGACACATATTGACACGTGATGTCTA	719
BC-8	GGGGGAGGTATAAGATAACCGAAGTTTTTCTTTTTTGTTTTTAATAATTTTTAATTTTTTTTTAATATTCACGTAGACACATATTGACACGTGATGTCTA	719
M9-1	GGGGGAGGTATAAGATAACCGAAGTTTTTCTTTTTTGTTTTTAATAATTTTTAATTTTTTTTTAATATTCACGTGGACACATATGGACACGTGATGTCTA	719
M9-2	GGGGGAGGTATGAGATAACCGAAGTTTTTCTTTTTTGTTTTTAATAATTTTTAATTTTTTTTTAATATTCACGTGGACACATATGGACACGTGATGTCTA	719
M9-3	GGGGGAGGTATAAGATAACCGAAGTTTTTCTTTTTTGTTTTTAATAATTTTTAATTTTTTTTTAATATTCACGTGGACACATATGGACACGTGATGTCTA	719
M9-4	GGGGGAGGTATAAGATAACCGAAGTTTTTCTTTTTTGTTTTTAATAATTTTTAATTTTTTTTTAATATTCACGTGGACACATATGGACACGTGATGTCTA	719
M9-5	GGGGGAGGTACAAGATAACCGAAGTTTTTCTTTTTTGTTTTTAATAATTTTTAATTTTTTTTTAATATTCACGTGGACACATATTGACACGTGATGTCTA	719
M9-6	GGGGGAGGTATAAGATAACCGAAGTTTTTCTTTTTTGTTTTTAATAATTTTTAATTTTTTTTTAATATTCACGTGGACACATATTGACACGTGATGTCTA	719
M9-7	GGGGGAGGTATAAGATAACCGAAGTTTTTCTTTTTTGTTTTTAATAATTTTTAATTTTTTTTTAATATTCACGTGGACACATATTGACACGTGATGTCTA	719
M9-8	GGGGGAGGTATAAGATAACCGAAGTTTTTCTTTTTTGTTTTTAATAATTTTTAATTTTTTTTTAATATTCACGTAGACACATATTGACACGTGATGTCTA	719

Reference	TTCATTGTCTACATGAACGTCACGTCACTAGTTAATGGCTGATTTAACAGCAATTTTAATAGATGTATGAAAACGTCCCAAAATTACTACTTCAGGTACC	800
BC-1	TTCATTGTCTACATGAACGTCACGTCACTAGTTAATGGCTGATTTAACAGCAATTTTAATAGATGTATGAAAACGTCCCAAAATTACGACTTCAGGTACC	802
BC-2	TTCATTGTCTACATGAACGTCACGTCACTAGTTAATGGCTGATTTAACAGCAATTTTAATAGATGTATGAAAACGTCCCAAAATTACGACTTCAGGTACC	802
BC-3	TTCATTGTCTACATGAACGTCACGTCACTAGTTAATGGCTGATTTAACAGCAATTTTAATAGATGTATGAAAACGTCCCAAAATTACGACTTCAGGTACC	828
BC-4	TTCATTGTCTACATGAACGTCACGTCACTAGTTAATGGCTGATTTAACAGCAATTTTAATAGATGTATGAAAACGTCCCAAAATTACGACTTCAGGTACC	813
BC-5	TTCATTGTCTACATGAACGTCACGTCACTAGTTAGTGGCTGATTTAACAGCAATTTTAATAGATGTATGAAAACGTCCCAAAATTACGACTTCAGGTACC	819
BC-6	TTCATTGTCTACATGAACGTCACGTCACTAGTTAATGGCTGATTTAACAGCAATTTTAATAGATGTATGAAAACGTCCCAAAATTACGACTTCAGGTACC	819
BC-7	TTCATTGTCTACATGAACGTCACGTCACTAGTTAATGGCTGATTTAACAGCAATTTTAATAGATGTATGAAAACGTCCCAAAATTACGACTTCAGGTACC	819
BC-8	TTCATTGTCTACATGAACGTCACGTCACTAGTTAATGGCTGATTTAACAGCAATTTTAATAGATGTATGAAAACGTCCCAAAATTACGACTTCAGGTACC	819
M9-1	TTCATTGTCTACATGAACGTCACGTCACTAGTTAATGGCTGATTTAACAGCAATTTTAATAGATGTATGAAAACGTCCCAAAATTACTACTTCAGGTACC	819
M9-2	TTCATTGTCTACATGAACGTCACGTCACTAGTTAATGGCTGATTTAACAGCAATTTTAATAGATGTATGAAAACGTCCCAAAATTACTACTTCAGGTACC	819
M9-3	TTCATTGTCTACATGAACGTCACGTCACTAGTTAATGGCTGATTTATCAGCAATTTTAATAGATGTATGAAACCGTCCCAAAATTACTACTTCAGGTACC	819
M9-4	TTCATTCTCTACATGAACGTCACGTCACTAGTTAATGGCTGATTTAACAGCAATTTTAATAGATGTATGAAAACGTCCCAAAATTACTACTTCAGGTACC	819
M9-5	TTCATTGTCTACATGAACGTCACGTCACTAGTTAGTGGCTGATTTAACAGCAATTTTAATAGATGTATGAAAACGTCCCAAAATTACTACTTCAGGTACC	819
M9-6	TTCATTGTCTACATGAACGTCACGTCACTAGTTAATGGCTGATTTAACAGCAATTTTAATAGATGTATGAAAACGTCCCAAAATTACTACTTCAGGTACC	819
M9-7	TTCATTGTCTACATGAACGTCACGTCACTAGTTAATGGCTGATTTAACAGCAATTTTAATAGATGTATGAAAACGTCCCAAAATTACTACTTCAGGTACC	819
M9-8	TTCATTGTCTACATGAACGTCACGTCACTAGTTAATGGCTGATTTAACAGCAATTTTAATAGATGTATGAAAACGTCCCAAAATTACTACTTCAGGTACC	819

Reference	ACTCTGGGATGAAAAAA.CTTTATGTATCAAATGTTGAAAACTAATAAACTTCAAGGTAGTAAACTGAAATTAACCCTAAATAAAATTTCCCAAATAAAA	899
BC-1	ACTCTGGTATGAAAAAAGTTTTATGTATCAAATGTTGAAAACTAACAAACTTCAAGGTAGTAAATTGATATTAATCCTAAATAAAATTTCCCAAATGGAA	902
BC-2	ACTCTGGGATGAAAAAAGTTTTATGTATCAAATGTTGAAAACTAACAAACTTCAAGGTAGTAAATTGATATTAATCCTAAATAAAATTTCCCAAATGGAA	902
BC-3	ACTCTGGTATGAAAAAAGTTTTATGTATCAAATGTTGAAAACTAACAAACTTCAAGGTAGTAAATTGATATTAATCCTAAATAAAATTTCCCAAATGGAA	928
BC-4	ACTCTGGTATGAAAAAAGTTTTATGTATCAAATGTTGAAAACTAACAAACTTCAAGGTAGTAAATTGATATTAATCCTAAATAAAATTTCCCAAATGGAA	913
BC-5	ACTCTGGTATGAAAAAAGTTTTATGTATCAAATGTTGAAAACTAACAAACTTCAAGGTAGTAAATTGATATTAATCCTAAATAAAATTTCCCAAATGGAA	919
BC-6	ACTCTGGTATGAAAAAAGTTTTATGTATCAAATGTTGAAAACTAACAAACTTCAAGGTAGTAAATTGATATTAATCCTAAATAAAATTTCCCAAATGGAA	919
BC-7	ACTCTGGTATGAAAAAAGTTTTATGTATCAAATGTTGAAAACTAACAAACTTCAAGGTAGTAAATTGATATTAATCCTAAATAAAATTTCCCAAATGGAA	919
BC-8	ACTCTGGTATGAAAAAAGTTTTATGTATCAAATGTTGAAAACTAACAAACTTCAAGGTAGTAAATTGATATTAATCCTAAATAAAATTTCCCAAATGGAA	919
M9-1	ACTCTGGGATGAAAAAA.CTTTATGTATCAAATGTTGAAAACTAATAAACTTCAAGGTAGTAAACTGAAATTAACCCTAAATAAAATTTCCCAAATAAAA	918
M9-2	ACTCTGGGATGAAAAAA.CTTTATGTATCAAATGTTGAAAACTAATAAACTTCAAGGTAGTAAACTGAAATTAACCCTAAATAAAATTTCCCAAATAAAA	918
M9-3	ACTCTGGGATGAAAAAA.CTTTATGTATCAAATGTTGAAAACTAATAAACTTCAAGGTAGTAAACTGAAATTAACCCTAAATAAAATTTCCCAAATAAAA	918
M9-4	ACTCTGGGATGAAAAAA.CTTTATGTATCAAATGTTGAAAACTAATAAACTTCAAGGTAGTAAACTGAAATTAACCCTAAATAAAATTTCCCAAATAAAA	918
M9-5	ACTCTGGGATGAAAAAA.CTTTATGTATCAAATGTTGAAAACTAATAAACTTCAAGGTAGTAAACTGAAATTAACCCTAAATAAAATTTCCCAAATAAAA	918
M9-6	ACTCTGGGATGAAAAAA.CTTTATGTATCAAATGTTGAAAACTAATAAACTTCAAGGTAGTAAACTGAAATTAACCCTAAATAAAATTTCCCAAATAAAA	918
M9-7	ACTCTGGGATGAAAAAA.CTTTATGTATCAAATGTTGAAAACTAATAAACTTCAAGGTAGTAAACTGAAATTAACCCTAAATAAAATTTCCCAAATAAAA	918
M9-8	ACTCTGGGATGAAAAAA.CTTTATGTATCAAATGTTGAAAACTAATAAACTTCAAGGTAGTAAACTGAAATTAACCCTAAATAAAATTTCCCAAATAAAA	918

Reference	AGTAGATAGTTATCCGCATTAGAGTTATGCGGTCGTACGCTTTCTCTTCACGTCTTTCAAGTGCTTCTATTCTCACTAATGGTGCAAAAGGGAAAATGGG	999
BC-1	AGTAGTTAGTTATCCGCATTAGAGTTATGCGGTCGTACGCTTTCTCTTCACGTCTTTCAAGTGCTTCTATTCTCACTAATGGTGCAAAAGGGAAAATGGG	1002
BC-2	AGTAGTTAGTTATCCGCATTAGAGTTATGCGGTCGTACGCTTTCTCTTCACGTCTTTCAAGTGCTTCTATTCTCACTAATGGTGCAAAAGGGAAAATGGG	1002
BC-3	AGTAGTTAGTTATCCGCATTAGAGTTATGCGGTCGTACGCTTTCTCTTCACGTCTTTCAAGTGCTTCTATTCTCACTAATGGTGCAAAAGGGAAAATGGG	1028
BC-4	AGTAGTTAGTTATCCGCATTAGAGTTATGCGGTCGTACGCTTTCTCTTCACGTCTTTCAAGTGCTTCTATTCTCACTAATGGTGCAAAAGGGAAAATGGG	1013
BC-5	AGTAGTTAGTTATCCGCATTAGAGTTATGCGGTCGTACGCTTTCTCTTCACGTCTTTCAAGTGCTTCTATTCTCACTAATGGTGCAAAAGGGAAAATGGG	1019
BC-6	AGTAGTTAGTTATCCGCATTAGAGTTATGCGGTCGTACGCTTTCTCTTCACGTCTTTCAAGTGCTTCTATTCTCACTAATGGTGCAAAAGGGAAAATGGG	1019
BC-7	AGTAGTTAGTTATCCGCATTAGAGTTATGCGGTCGTACGCTTTCTCTTCACGTCTTTCAAGTGCTTCTATTCTCACTAATGGTGCAAAAGGGAAAATGGG	1019
BC-8	AGTAGTTAGTTATCCGCATTAGAGTTATGCGGTCGTACGCTTTCTCTTCACGTCTTTCAAGTGCTTCTATTCTCACTAATGGTGCAAAAGGGAAAATGGG	1019
M9-1	AGTAGATAGTTATCCGCATTAGAGTTATGCGGTCGTACGCTTTCTCTTCACGTCTTTCAAGTGCTTCTATTCTCACTAATGGTGCAAAAGGGAAAATGGG	1018
M9-2	AGTAGATAGTTATCCGCATTAGAGTTATGCGGTCGTACGCTTTCTCTTCACGTCTTTCAAGTGCTTCTATTCTCACTAATGGTGCAAAAGGGAAAATGGG	1018
M9-3	AGTAGATAGTTATCCGCATTAGAGTTATGCGGTCGTACGCTTTCTCTTCACGTCTTTCAAGTGCTTCTATTCTCACTAATGGTGCAAAAGGGAAAATGGG	1018
M9-4	AGTAGATAGTTATCCGCATTAGAGTTATGCGGTCGTACGCTTTCTCTTCACGTCTTTCAAGTGCTTCTATTCTCACTAATGGTGCAAAAGGGAAAATGGG	1018
M9-5	AGTAGATAGTTATCCGCATTAGAGTTATGCGGTCGTACGCTTTCTCTTCACGTCTTTCAAGTGCTTCTATTCTCACTAATGGTGCAAAAGGGAAAATGGG	1018
M9-6	AGTAGATAGTTATCCGCATTAGAGTTATGCGGTCGTACGCTTTCTCTTCACGTCTTTCAAGTGCTTCTATTCTCACTAATGGTGCAAAAGGGAAAATGGG	1018
M9-7	AGTAGATAGTTATCCGCATTAGAGTTATGCGGTCGTACGCTTTCTCTTCACGTCTTTCAAGTGCTTCTATTCTCACTAATGGTGCAAAAGGGAAAATGGG	1018
M9-8	AGTAGATAGTTATCCGCATTAGAGTTATGCGGTCGTACGCTTTCTCTTCACGTCTTTCAAGTGCTTCTATTCTCACTAATGGTGCAAAAGGGAAAATGGG	1018

```
Reference   ACTGTCCATATGCTTACGCGTTAGAAGATGCAGGGACCATGTGTTGCTAGCACTAAAACATACCATACACACGCATTGCAGAAATTAAGAGAGGGTAAAA   1099
BC-1        ACTGTCCATATGCTTACGCGTTAGAAGATGCAGGGACCATGTGTTGCTAGCACTAAA.CATACCATACACACGCATTGCAGAAATTAAGAGAGGGGAAAA   1101
BC-2        ACTGTCCATATGCTTACGCGTTAGAAGATGCAGGGACCATGTGTTGCTAGCACTAAA.CATACCATACACACGCATTGCAGAAATTAAGAGAGGGGAAAA   1101
BC-3        ACTGTCCATATGCTTACGCGTTAGAAGATGCAGGGACCATGTGTTGCTAGCACTAAA.CATACCATACACACGCATTGCAGAAATTAAGAGAGGGGAAAA   1127
BC-4        ACTGTCCATATGCTTACGCGTTAGAAGATGCAGGGACCATGTGTTGCTAGCACTAAA.CATACCATACACACGCATTGCAGAAATTAAGAGAGGGGAAAA   1112
BC-5        ACTGTCCATATGCTTACGCGTTAGAAGATGCAGGGACCATGTGTTGCTAGCACTAAA.CATACCATACACACGCATTGCAGAAATTAAGAGAGGGGAAAA   1118
BC-6        ACTGTCCATATGCTTACGCGTTAGAAGATGCAGGGACCATGTGTTGCTAGCACTAAA.CATACCATACACACGCATTGCAGAAATTAAGAGAGGGGAAAA   1118
BC-7        ACTGTCCATATGCTTACGCGTTAGAAGATGCAGGGACCATGTGTTGCTAGCACTAAA.CATACCATACACACGCATTGCAGAAATTAAGAGAGGGGAAAA   1118
BC-8        ACTGTCCATATGCTTACGCGTTAGAAGATGCAGGGACCATGTGTTGCTAGCACTAAA.CATACCATACACACGCATTGCAGAAATTAAGAGAGGGGAAAA   1118
M9-1        ACTGTCCATATGCTTACGCGTTAGAAGATGCAGGGACCATGTGTTGCTAGCACTAAAACATACCATACACACGCATTGCAGAAATTAAGAGAGGGTAAAA   1118
M9-2        ACTGTCCATATGCTTACGCGTTAGAAGATGCAGGGACCATGTGTTGCTAGCACTAAAACATACCATACACACGCATTGCAGAAATTAAGAGAGGGTAAAA   1118
M9-3        ACTGTCCATATGCTTACGCGTTAGAAGATGCAGGGACCATGTGTTGCTAGCACTAAAACATACCATACACACGCATTGCAGAAATTAAGAGAGGGTAAAA   1118
M9-4        ACTGTCCATATGCTTACGCGTTAGAAGATGCAGGGACCATGTGTTGCTAGCACTAAAACATACCATACACACGCATTGCAGAAATTAAGAGAGGGTAAAA   1118
M9-5        ACTGTCCATATGCTTACGCGTTAGAAGATGCAGGGACCATGTGTTGCTAGCACTAAAACATACCATACACACGCATTGCAGAAATTAAGAGAGGGTAAAA   1118
M9-6        ACTGTCCATATGCTTACGCGTTAGAAGATGCAGGGACCATGTGTTGCTAGCACTAAAACATACCATACACACGCATTGCAGAAATTAAGAGAGGGTAAAA   1118
M9-7        ACTGTCCATATGCTTACGCGTTAGAAGATGCAGGGACCATGTGTTGCTAGCACTAAAACATACCATACACACGCATTGCAGAAATTAAGAGAGGGTAAAA   1118
M9-8        ACTGTCCATATGCTTACGCGTTAGAAGATGCAGGGACCATGTGTTGCTAGCACTAAAACATACCATACACACGCATTGCAGAAATTAAGAGAGGGTAAAA   1118

Reference   TATGCATTTGTTTTATTTTAGTTTGTATAAAACTTACTTCTTATGTATTATCTAAAGGACAATGTCAGGATGATCAAATTTCTAAGTTAAATATGCAAAA   1199
BC-1        TATGCATTTGTTTTATTTTAGTTTGTATAAAACTTACTTTTTATGTATTATCTAAAGGACAATGTCAGGATGATTAAATTTCTAAGCTAAATATGCAAAA   1201
BC-2        TATGCATTTGTTTTATTTTAGTTTGTATAAAACTTACTTTTTATGTATTATCTAAAGGACAATGTCAGGATGATTAAATTTCTAAGCTAAATATGCAAAA   1201
BC-3        TATGCATTTGTTTTATTTTAGTTTGTATAAAACTTACTTTTTATGTATTATCTAAAGGACAATGTCAGGATGATTAAATTTCTAAGCTAAATATGCAAAA   1227
BC-4        TATGCATTTGTTTTATTTTAGTTTGTATAAAACTTACTTTTTATGTATTATCTAAAGGACAATGTCAGGATGATTAAATTTCTAAGCTAAATATGCAAAA   1212
BC-5        TATGCATTTGTTTTATTTTAGTTTGTATAAAACTTACTTTTTATGTATTATCTAAAGGACAATGTCAGGATGATTAAATTTCTAAGCTAAATATGCAAAA   1218
BC-6        TATGCATTTGTTTTATTTTAGTTTGTATAAAACTTACTTTTTATGTATTATCTAAAGGACAATGTCAGGATGATTAAATTTCTAAGCTAAATATGCAAAA   1218
BC-7        TATGCATTTGTTTTATTTTAGTTTGTATAAAACTTACTTTTTATGTATTATCTAAAGGACAATGTCAGGATGATTAAATTTCTAAGCTAAATATGCAAAA   1218
BC-8        TATGCATTTGTTTTATTTTAGTTTGTATAAAACTTACTTTTTATGTATTATCTAAAGGACAATGTCAGGATGATTAAATTTCTAAGCTAAATATGCAAAA   1218
M9-1        TATGCATTTGTTTTATTTTAGTTTGTATAAAACTTACTTCTTATGTATTATCTAAAGGACAATGTCAGGATGATCAAATTTCTAAGTTAAATATGCAAAA   1218
M9-2        TATGCATTTGTTTTATTTTAGTTTGTATAAAACTTACTTCTTATGTATTATCTAAAGGACAATGTCAGGATGATCAAATTTCTAAGTTAAATATGCAAAA   1218
M9-3        TATGCATTTGTTTTATTTTAGTTTGTATAAAACTTACTTCTTATGTATTATCTAAAGGACAATGTCAGGATGATCAAATTTCTAAGTTAAATATGCAAAA   1218
M9-4        TATGCATTTGTTTTATTTTAGTTTGTATAAAACTTACTTCTTATGTATTATCTAAAGGACAATGTCAGGATGATCAAATTTCTAAGTTAAATATGCAAAA   1218
M9-5        TATGCATTTGTTTTATTTTAGTTTGTATAAAACTTACTTCTTATGTATTATCTAAAGGACAATGTCAGGATGATCAAATTTCTAAGTTAAATATGCAAAA   1218
M9-6        TATGCATTTGTTTTATTTTAGTTTGTATAAAACTTACTTCTTATGTATTATCTAAAGGACAATGTCAGGATGATCAAACTTCTAAGTTAAATATGCAAAA   1218
M9-7        TATGCATTTGTTTTATTTTAGTTTGTATAAAACTTACTTCTTATGTATTATCTAAAGGACAATGTCAGGATGATCAAATTTCTAAGTTAAATATGCAAAA   1218
M9-8        TATGCATTTGTTTTATTTTAGTTTGTATAAAACTTACTTCTTATGTATTATCTAAAGGACAATGTCAGGATGATCAAATTTCTAAGTTAAATATGCAAAA   1218

Reference   TGTATCACTAATAAGAAACAAAAGTTAGTAATTAGAATAAGTCTCTTGTATTGGCGTATGCGAAAAAGTTAATAACTAGACAATTTAAAACAGAAGATAG   1299
BC-1        TGTATCATTAATAAGAAACAAAAGTTAGTAATTAGAATAAGTCTCTTGTATTGGCGCATGCGAAAAAGTTAGTAACTAGACAATTTAAAACAAAAGATAG   1301
BC-2        TGTGTCATTAATAAGAAACAAAAGTTAGTAATTAGAATAAGTCTCTTGTATTGGCGCATGCGAAAAAGTTAGTAACTAGACAATTTAAAACAAAAGATAG   1301
BC-3        TGTACCATTAATAAGAAACAAAAGTTAGTAATTAGAATAAGTCTCTTGTATTGGCGCATGCGAAAAAGTTAGTAACTAGACAATTTAAAACAAAAGATAG   1327
BC-4        TGTATCATTAATAAGAAACAGAAGTTAGTAATTAGAATAAGTCTCTTGTATTGGCGCATGCGAAAAAGTTAGTAACTAGACAATTTAAAACAAAAGATAG   1312
BC-5        TGTATCATTAATAAGAAACAAAAGTTAGTAATTAGAATAAGTCTCTTGTATTGGCGCATGCGAAAAAGTTAGTAACTAGACAATTTAAAACAAAAGATAG   1318
BC-6        TGTATCATTAATAAGAAACAAAAGTTAGTAATTAGAATAAGTCTCTTGTATTGGCGCATGCGAAGAAGTTAGTAACTAGACAATTTAAAACAAAAGATAG   1318
BC-7        TGTATCATTAATAAGAAACAAAAGTTAGTAATTAGAATAAGTCTCTTGTATTGGCGCATGCGAAGAAGTTAGTAACTAGACAATTTAAAACAAAAGATAG   1318
BC-8        TGTATCATTAATAAGAAACAAAAGTTAGTAATTAGAATAAGTCTCTTGTATTGGCGCATGCGAAGAAGTTAGTAACTAGACAATTTAAAACAAAAGATAG   1318
M9-1        TGTATCACTAATAAGAAACAAAAGTTAGTAATTAGAATAAGTCTCTTGTATTGGCGTATGCGAAAAAGTTAATAACTAGACAATTTAAAACAGAAGATAG   1318
M9-2        TGTATCACTAATAAGAAACAAAAGTTAGTAATTAGAATAAGTCTCTTGTATTGGCGTATGCGAAAAAGTTAATAACTAGACAATTTAAAACAGAAGATAG   1318
M9-3        TGTATCACTAATAAGAAACAAAAGTTAGTAATTAGAATAAGTCTCTTGTATTGGCGTATGCGAAAAAGTTAATAACTAGACAATTTAAAACAGAAGATAG   1318
M9-4        TGTATCACTAATAAGAAACAAAAGTTAGTAATTAGAATAAGTCTCTTGTATTGGCGTATGCGAAAAAGTTAATAACTAGACAATTTAAAACAGAAGATAG   1318
M9-5        TGTATCACTAATAAGAAACAAAAGTTAGTAATTAGAATAAGTCTCTTGTATTGGCGTATGCGAAAAAGTTAATAACTAGACAATTTAAAACAGAAGATAG   1318
M9-6        TGTATCACTAATAAGAAACAAAAGTTAGTAATTAGAATAAGTCTCTTGTATTGGCGTATGCGAAAAAGTTAATAACTAGACAATTTAAAACAGAAGATAG   1318
M9-7        TGTATCACTAATAAGAAACAAAAGTTAGTAATTAGAATAAGTCTCTTGTATTGGCGTATGCGAAAAAGTTAATAACTAGACAATTTAAAACAGAAGATAG   1318
M9-8        TGTATCACTAATAAGAAACAAAAGTTAGTAATTAGAATAAGTCTCTTGTATTGGCGTATGCGAAAAAGTTAATAACTAGACAATTTAAAACAGAAGATAG   1318

Reference   TTGAATTGTAATGTAATTATTTGAATAACATACTTATTTTTTTATTAATGATATATCATTTAAT.GGAATAAGAATTAAGATACCTCTGCAACCAAAAAAGC   1398
BC-1        TTGAATTGTAATGTAATTATTTGAATAACATACTTATTTTTTTATTTTTTATTGATAATATACCAGGGATAAGAATTAAGATACCTCTGCGACCAAAAAAGC   1401
BC-2        TTGAATTGTAATGTAATTATTTGAATAACATACTTATTTTTTTATTTTTTATTGATAATATACCAGGGATAAGAATTAAGATACCTCTGCGACCAAAAAAGC   1401
BC-3        TTGAATTGTAATGTAATTATTTGAATAACATACTTATTTTTTTATTTTTTATTGATAATATACCAGGGATAAGAATTAAGATACCTCTGCGACCAAAAAAGC   1427
BC-4        TTGAATTGTAATGTAATTATTTGAATAACATACTTATTTTTTTATTTTTTATTGATAATATACCAGGGATAAGAATTAAGATACCTCTGCGACCAAAAAAGC   1412
BC-5        TTGAATTGTAATGTAATTATTTGAATAACATACTTATTTTTTTATTTTTTATTGATAATATACCAGGGATAAGAATTAAGATACCTCTGCGACCAAAAAAGC   1418
BC-6        TTGAATTGTAATGTAATTATTTGAATAACATACTTATTTTTTTATTTTTTATTGATAATATACCAGGGATAAGAATTAAGATACCTCTGCGACCAAAAAAGC   1418
BC-7        TTGAATTGTAATGTAATTATTTGAATAACATACTTATTTTTTTATTTTTTATTGATAATATACCAGGGATAAGAATTAAGATACCTCTGCGACCAAAAAAGC   1418
BC-8        TTGAATTGTAATGTAATTATTTGAATAACATACTTATTTTTTTATTTTTTATTGATAATATACCAGGGATAAGAATTAAGATACCTCTGCGACCAAAAAAGC   1418
M9-1        TTGAATTGTAATGTAATTATTTGAATAACATACTTATTTTTTTATTAATGATATATCATTTAAT.GGAATAAGAATTAAGATACCTCTGCAACCAAAAAAGC   1417
M9-2        TTGAATTGTAATGTAATTATTTGAATAACATACTTATTTTTTTATTAATGATATATCATTTAAT.GGAATAAGAATTAAGATACCTCTGCAACCAAAAAAGC   1417
M9-3        TTGAATTGTAATGTAATTACTGAATAACATACTTATTTTTTTATTAACGATATATCATTTAAT.GGAATAAGAATTAAGATACCTCTGCAACCAAAAAAGC   1417
M9-4        TTGAATTGTAATGTAATTATTTGAATAACATACTTATTTTTTTATTAATGATATATCATTTAAT.GGAATAAGAATTAAGATACCTCTGCAACCAAAAAAGC   1417
M9-5        TTGAATTGTAATGTAATTATTTGAATAACATACTTATTTTTTTATTAATGATATATCATTTAAT.GGAATAAGAATTAAGATACCTCTGCAACCAAAAAAGC   1417
M9-6        TTGAATTGTAATGTAATTATTTGAATAACATACTTATTTTTTTATTAATGATATATCATTTAAT.GGAATAAGAATTAAGATACCTCTGCAACCAAAAAAGC   1417
M9-7        TTGAATTGTAATGTAATTATTTGAATAACATACTTATTTTTTTATTAATGATATATCATTTAAT.GGAATAAGAATTAAGATACCTCTGCAACCAAAAAAGC   1417
M9-8        TTGAATTGTAATGTAATTATTTGAATAACATACTTATTTTTTTATTAATGATATATCATTTAAT.GGAATAAGAATTAAGATACCTCTGCAACCAAAAAAGC   1417

Reference   ATCACTGAGGGTCGAAAAAGATACTCAAATGGTAGAAGAAAGCGTTACAAAAATTGGACCGCCCAGCGGGCCACGCCCACATGCTTAGATGCCCACGCTA   1498
BC-1        ATCACCGAGGGTCGAAAAAGATACTCAAATGGTAGAAGAAAGCGTTACAAAAATTGGACCGCCCAGCGGGCCACCCCCACATGCTTAGATGCCCACGCTA   1501
BC-2        ATCACCGAGGGTCGAAAAAGATACTCAAATGGTAGAAGAAAGCGTTACAAAAATTGGACCGCCCAGCGGGCCACCCCCACATGCTTAGATGCCCACGCTA   1501
BC-3        ATCACCGAGGGTCGAAAAAGATACTCAAATGGTAGAAGAAAGCGTTACAAAAATTGGACCGCCCAGCGGGCCACCCTCACATGCTTAGATGCCCACGCTA   1527
BC-4        ATCACCGAGGGTCGAAAAAGATACTCAAATGGTAGAAGAAAGCGTTACAAAAATTGGACCGCCCAGCGGGCCACCCCCACATGCTTAGATGCCCACGCTA   1512
BC-5        ATCACCGAGGGTCGAAAAAGATACTCAAATGGTAGAAGAAAGCGTTACAAAAATTGGACCGCCCAGCGGGCCACCCCCACATGCTTAGATGCCCACGCTA   1518
BC-6        ATCACCGAGGGTCGAAAAAGATACTCAAATGGTAGAAGAAAGCGTTACAAAAATTGGACCGCCCAGCGGGCCACCCCCACATGCTTAGATGCCCACGCTA   1518
BC-7        ATCACCGAGGGTCGAAAAAGATACTCAAATGGTAGAAGAAAGCGTTACAAAAATTGGACCGCCCAGCGGGCCACCCCCACATGCTTAGATGCCCACGCTA   1518
BC-8        ATCACCGAGGGTCGAAAAAGATACTCAAATGGTAGAAGAAAGCGTTACAAAAATTGGACCGCCCAGCGGGCCACCCCCACATGCTTAGATGCCCACGCTA   1518
M9-1        ATCACTGAGGGTCGAAAAAGATACTCAAATGGTAGAAGAAAGCGTTACAAAAATTGGACCGCCCAGCGGGCCACGCCCACATGCTTAGATGCCCACGCTA   1517
M9-2        ATCACTGAGGGTCGAAAAAGATACTCAAATGGTAGAAGAAAGCGTTACAAAAATTGGACCGCCCAGCGGGCCACGCCCACATGCTTAGATGCCCACGCTA   1517
M9-3        ATCACTGAGGGTCGAAAAAGATACTCAAATGGTAGAAGAAAGCGTTACAAAAATTGGACCGCCCAGCGGGCCACGCCCACATGCTTAGATGCCCACGCTA   1517
M9-4        ATCACTGAGGGTCGAAAAAGATACTCAAATGGTAGAAGAAAGCGTTACAAAAATTGGACCGCCCAGCGGGCCACGCCCACATGCTTAGATGCCCACGCTA   1517
M9-5        ATCACTGAGGGTCGAAAAAGATACTCAAATGGTAGAAGAAAGCGTTACAAAAATTGGACCGCCCAGCGGGCCACGCCCACATGCTTAGATGCCCACGCTA   1517
M9-6        ATCACTGAGGGTCGAAAAAGATACTCAAATGGTAGAAGAAAGCGTTACAAAAATTGGACCGCCCAGCGGGCCACGCCCACATGCTTAGATGCCCACGCTA   1517
M9-7        ATCACTGAGGGTCGAAAAAGATACTCAAATGGTAGAAGAAAGCGTTACAAAAATTGGACCGCCCAGCGGGCCACGCCCACATGCTTAGATGCCCACGCTA   1517
```

Reference	AC	1500
BC-1	AC	1503
BC-2	AC	1503
BC-3	AC	1529
BC-4	AC	1514
BC-5	AC	1520
BC-6	AC	1520
BC-7	AC	1520
BC-8	AC	1520
M9-1	AC	1519
M9-2	AC	1519
M9-3	AC	1519
M9-4	AC	1519
M9-5	AC	1519
M9-6	AC	1519
M9-7	AC	1519
M9-8	AC	1519
