# Supplementary figures and images for: Genomics-assisted prediction of salt and alkali tolerances and functional marker development in apple rootstocks
Source: BMC Genomics. 2020 Aug 10;21:550. doi: 10.1186/s12864-020-06961-9 (PMC7430842; doi:10.1186/s12864-020-06961-9)

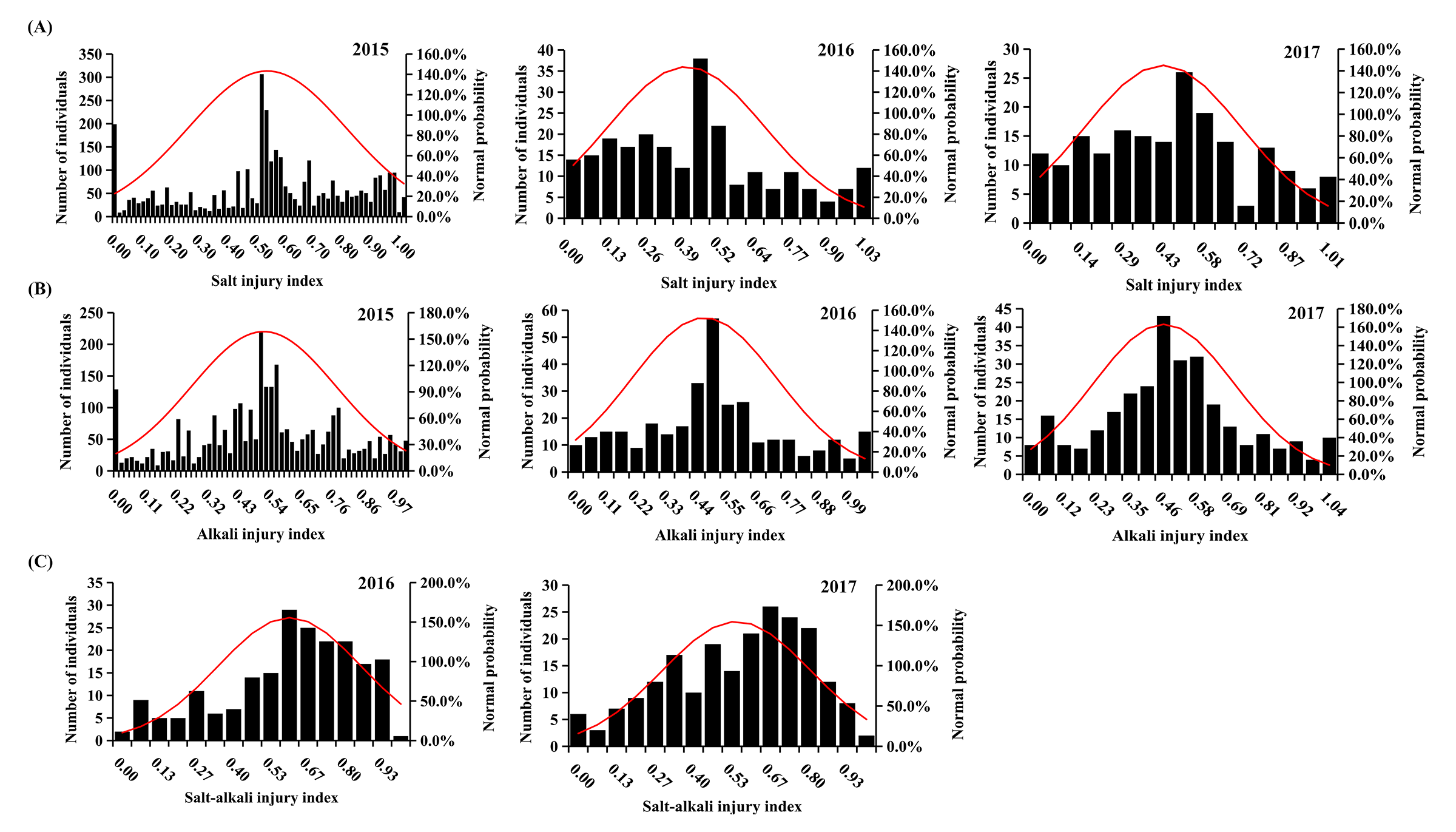

Supplement: Supplementary file 6 — Additional file 6: Fig. S1. Frequency distributions of salt (A), alkali (B), salt-alkali (C) injury indices in apple rootstock F1 hybrids of Malus robusta Rehd. ‘Baleng Crab’ × M. pumila Mill. ‘M9’ in 2015–2017. [file 12864_2020_6961_MOESM6_ESM.tif]

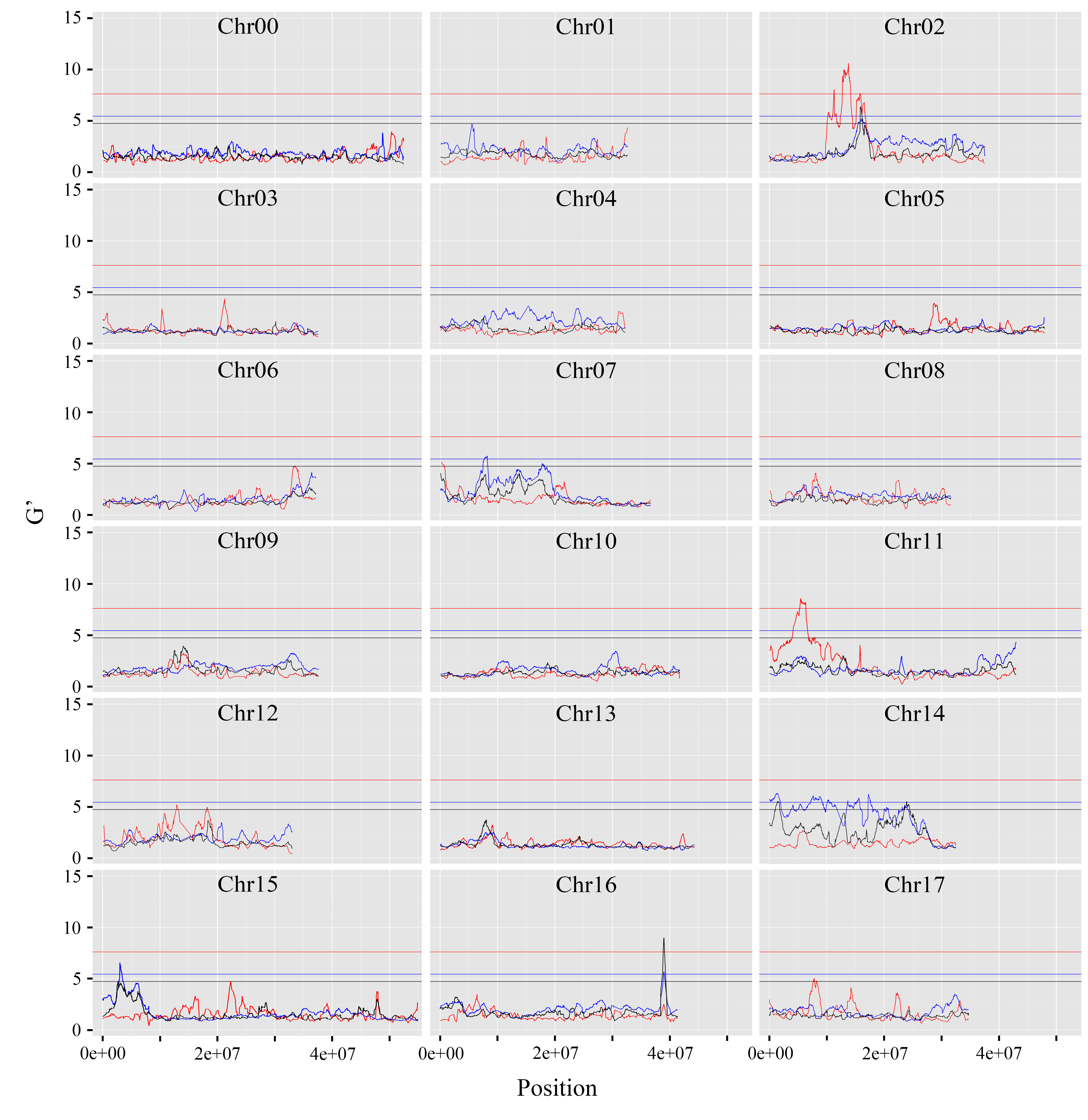

Supplement: Supplementary file 7 — Additional file 7: Fig. S2. Diagrams showing quantitative trait loci (QTLs) for salt injury index identified using bulked segregant analysis by sequencing in F1 hybrids of apple rootstocks Malus robusta Rehd. ‘Baleng Crab (BC)’ × M. pumila Mill. ‘M9’. Y-axis represents the G’ value, X-axis represents chromosome physical position. The red curved lines: ‘M9’, blue curved lines: ‘BC’, black curved lines: ‘BC’ & ‘M9’. The horizontal lines with colors indicate the corresponding statistic significant threshold of G’ value. [file 12864_2020_6961_MOESM7_ESM.tif]

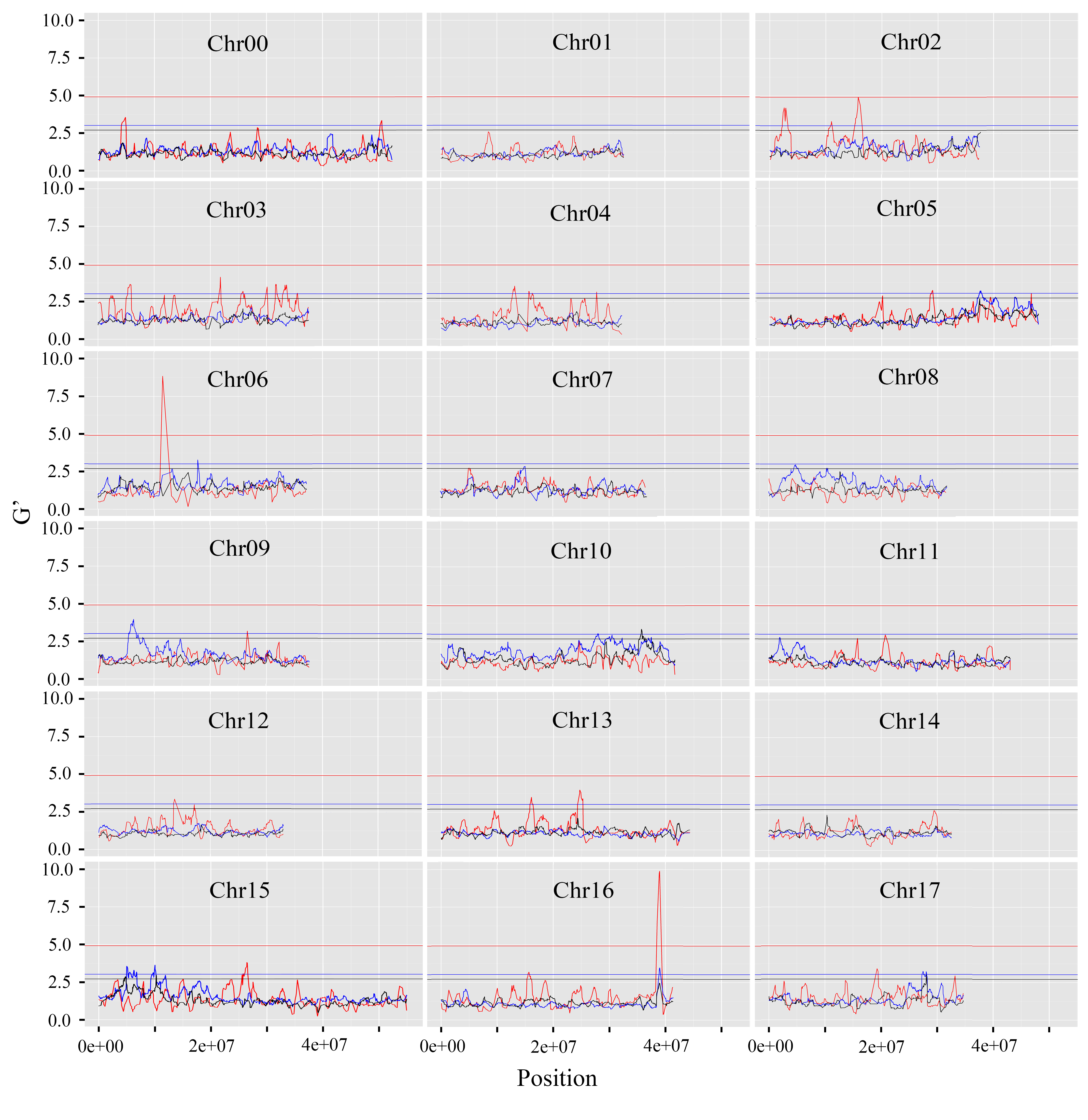

Supplement: Supplementary file 8 — Additional file 8: Fig. S3. Diagrams showing quantitative trait loci (QTLs) for alkali injury index identified using bulked segregant analysis by sequencing in F1 hybrids of apple rootstocks Malus robusta Rehd. ‘Baleng Crab (BC)’ × M. pumila Mill. ‘M9’. Y-axis represents G’ value, X-axis represents chromosome physical position. The red curved lines: ‘M9’, blue curved lines: ‘BC’, black curved lines: ‘BC’ & ‘M9’. The horizontal lines with colors indicate the corresponding statistic significant threshold of G’ value. [file 12864_2020_6961_MOESM8_ESM.tif]

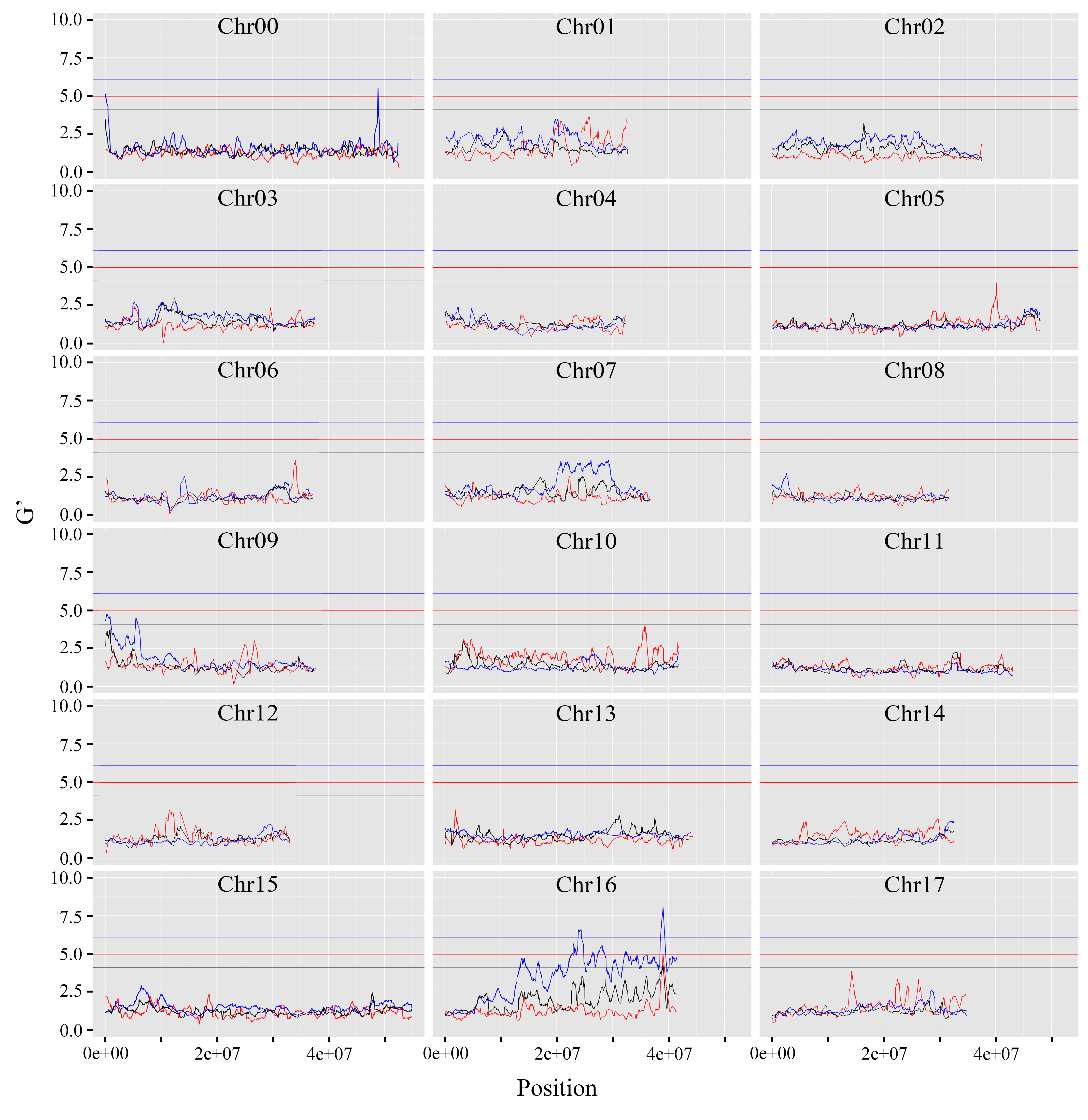

Supplement: Supplementary file 9 — Additional file 9: Fig. S4. Diagrams showing quantitative trait loci (QTLs) for salt-alkali injury index identified using bulked segregant analysis by sequencing in F1 hybrids of apple rootstocks Malus robusta Rehd. ‘Baleng Crab (BC)’ × M. pumila Mill. ‘M9’. Y-axis represents G’ value, X-axis represents chromosome physical position. The red curved lines: ‘M9’, blue curved lines: ‘BC’, black curved lines: ‘BC’ & ‘M9’. The horizontal lines with colors indicate the corresponding statistic significant threshold of G’ value. [file 12864_2020_6961_MOESM9_ESM.tif]

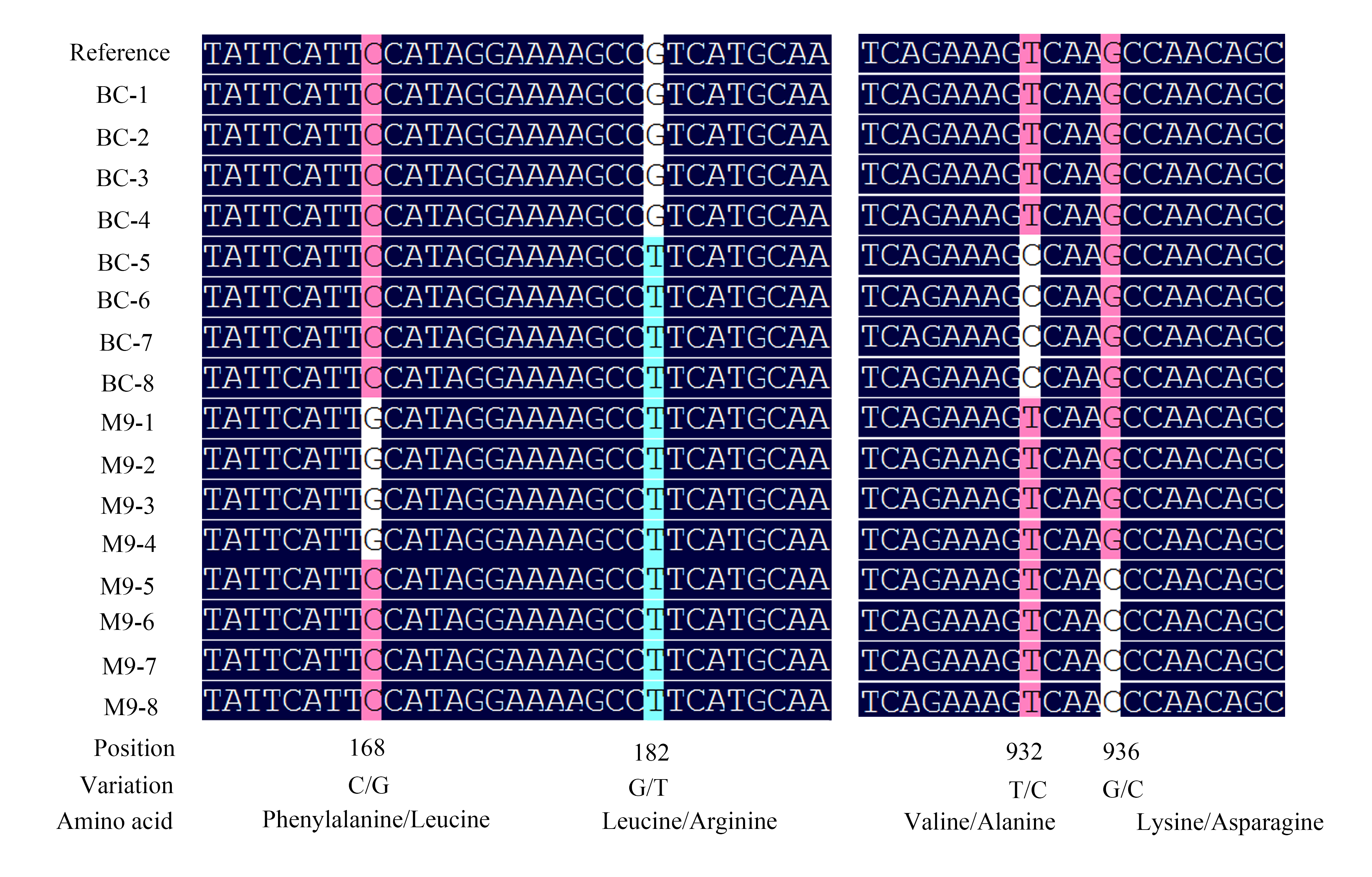

Supplement: Supplementary file 10 — Additional file 10: Fig. S5. Sanger sequencing confirmed allelic variations in the coding region of MdRGLG3 between Malus robusta Rehd. ‘Baleng Crab (BC)’ × M. pumila Mill. ‘M9’. [file 12864_2020_6961_MOESM10_ESM.tif]

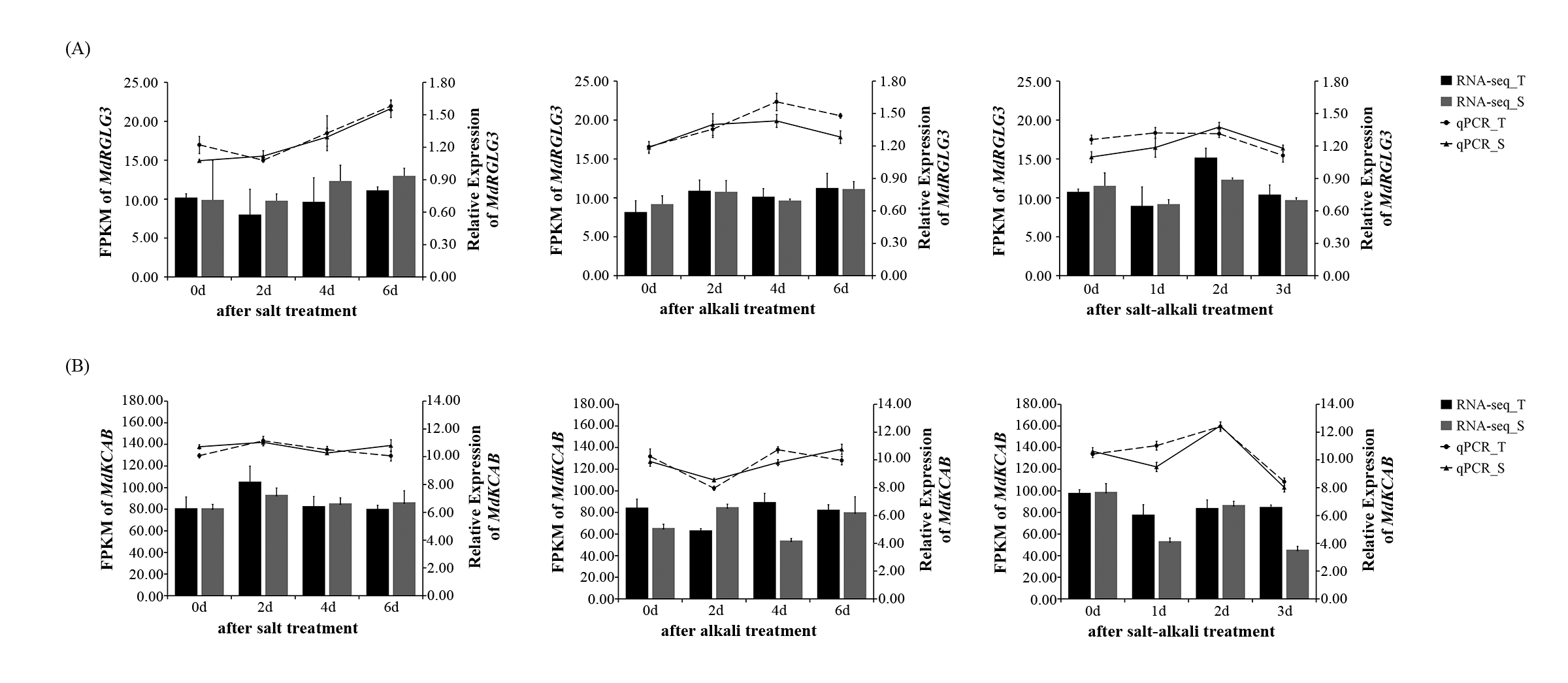

Supplement: Supplementary file 11 — Additional file 11: Fig. S6. Dynamic changes in expression of MdRGLG3 (A) and MdKCAB (B) in salt, alkali and salt-alkali tolerant (T) or sensitive (S) hybrids of apple rootstocks Malus robusta Rehd. ‘Baleng Crab’ × M. pumila Mill. ‘M9’. The gene expressions were shown in fragments per kilobase per million (FPKM) by RNA-seq and relative expression by qPCR. [file 12864_2020_6961_MOESM11_ESM.tif]

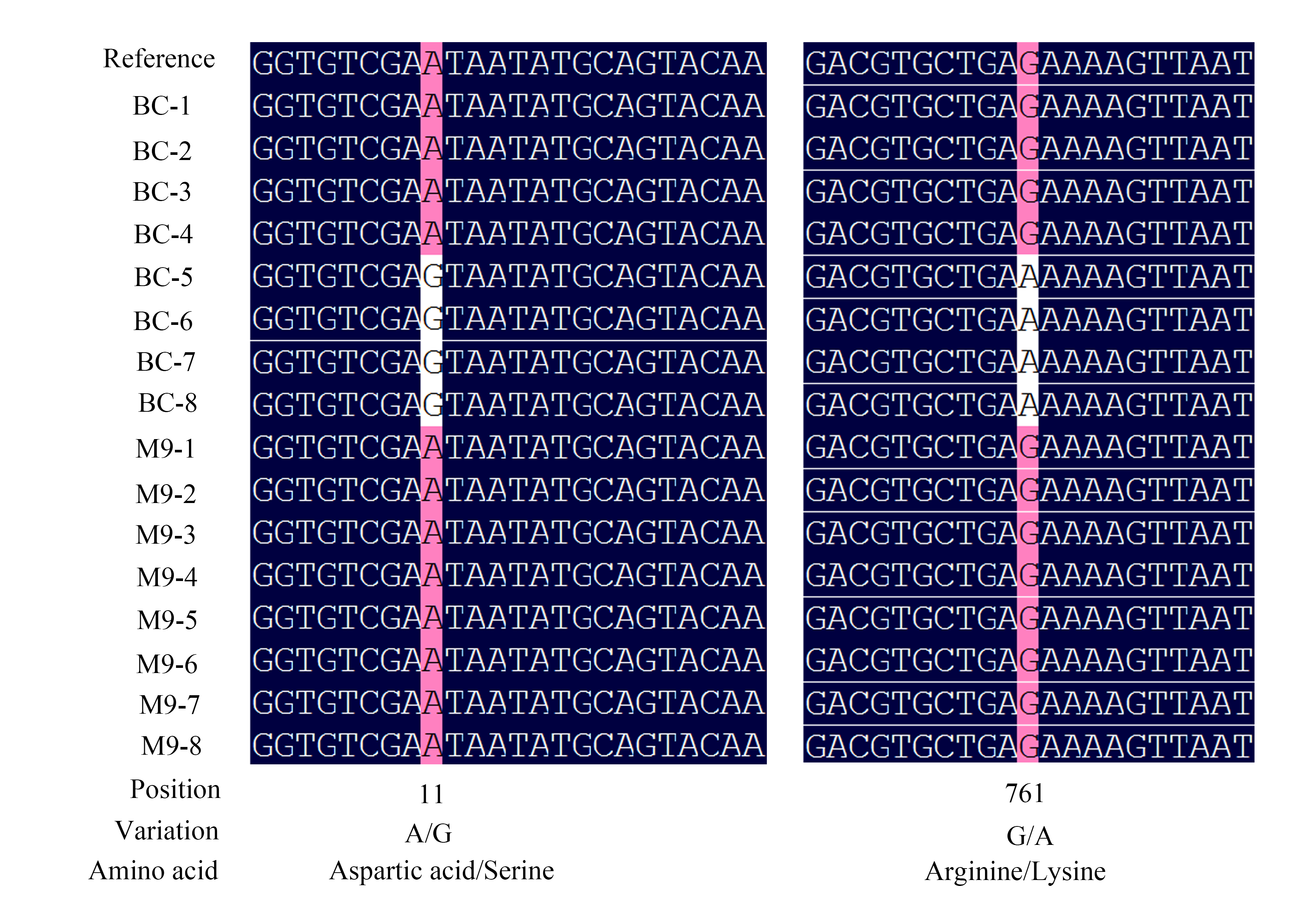

Supplement: Supplementary file 12 — Additional file 12: Fig. S7. Sanger sequencing confirmed allelic variations in the coding region of MdKCAB between Malus robusta Rehd. ‘Baleng Crab (BC)’ × M. pumila Mill. ‘M9’. [file 12864_2020_6961_MOESM12_ESM.tif]
